# A machine learning approach utilizing DNA methylation as an accurate classifier of COVID-19 disease severity

**DOI:** 10.1038/s41598-022-22201-4

**Published:** 2022-10-19

**Authors:** Scott Bowler, Georgios Papoutsoglou, Aristides Karanikas, Ioannis Tsamardinos, Michael J. Corley, Lishomwa C. Ndhlovu

**Affiliations:** 1 Division of Infectious Diseases, Department of Medicine, Weill Cornell Medicine, 413 E 69th St, New York, NY 10021 USA; 2 JADBio – Gnosis DA S.A, Science and Technology Park of Crete, 70013 Heraklion, Greece; 3 Department of Computer Science, University of Crete, 70013 Heraklion, Greece

**Keywords:** Machine learning, Infectious diseases

## Abstract

Since the onset of the COVID-19 pandemic, increasing cases with variable outcomes continue globally because of variants and despite vaccines and therapies. There is a need to identify at-risk individuals early that would benefit from timely medical interventions. DNA methylation provides an opportunity to identify an epigenetic signature of individuals at increased risk. We utilized machine learning to identify DNA methylation signatures of COVID-19 disease from data available through NCBI Gene Expression Omnibus. A training cohort of 460 individuals (164 COVID-19-infected and 296 non-infected) and an external validation dataset of 128 individuals (102 COVID-19-infected and 26 non-COVID-associated pneumonia) were reanalyzed. Data was processed using ChAMP and beta values were logit transformed. The JADBio AutoML platform was leveraged to identify a methylation signature associated with severe COVID-19 disease. We identified a random forest classification model from 4 unique methylation sites with the power to discern individuals with severe COVID-19 disease. The average area under the curve of receiver operator characteristic (AUC-ROC) of the model was 0.933 and the average area under the precision-recall curve (AUC-PRC) was 0.965. When applied to our external validation, this model produced an AUC-ROC of 0.898 and an AUC-PRC of 0.864. These results further our understanding of the utility of DNA methylation in COVID-19 disease pathology and serve as a platform to inform future COVID-19 related studies.

## Introduction

Since the first reports of an acute atypical respiratory disease in Wuhan, China in December 2019, the novel coronavirus known as severe acute respiratory syndrome coronavirus-2 (SARS-CoV-2) was soon identified as the cause of the disease known as Coronavirus disease 19 (COVID-19). Having been declared a pandemic by the World Health Organization (WHO), COVID-19 has affected many people worldwide. As of October 20, 2021, more than 240,000,000 cases and 4,900,000 deaths have been reported in over 200 countries^1^. Initial cases from Wuhan, China reported SARS-CoV-2 primarily affected the respiratory system with symptoms including fever, dry cough, dyspnea, as well as, headache, dizziness, generalized weakness, vomiting, and diarrhea^2^. However, complications involving nearly all organ systems including pulmonary^3,4^, cardiac^5^, renal^6^, and neurological^7,8^ systems have since been reported. The mechanism driving these changes are multifactorial. SARS-CoV-2-mediated epigenetic changes have been implicated as a contributor to COVID-19 systemic toxicity and lethality^9^, suggesting DNA methylation (DNAm) signatures may provide a biomarker to predict disease severity and identify therapeutic targets.

DNAm refers to the process by which methyl groups are added to the C-5 position of the cytosine ring of DNA by DNA methyltransferases. This process plays a role in cellular development and aging, gene imprinting, X inactivation, and gene silencing to ensure genomic stability^10,11^. DNAm is a reversible biological process^12^ and methylation patterns have been linked to treatment response in rheumatoid arthritis^13^, neurological disorders^14^, and cancer^15,16^. Furthermore, DNAm has been reported to modify interferon-stimulated gene responses and antigen presentation post MERS-CoV infection^17,18^.

DNAm of angiotensin-converting enzyme 2 (ACE2), type II transmembrane serine protease 2 (TMPRSS2), and a disintegrin and metalloproteinase domain 17 (ADAM17) have been extensively studied largely due to their involvement of SARS-CoV-2 gaining entry into host cells^19,20^. Genome-wide methylation alternations by SARS-CoV-2 infection and their impact upon disease severity and clinical outcomes however remain largely understudied. We were the first to report that hypermethylation of interferon-related genes, hypomethylation of inflammatory genes, and perturbations of the epigenetic clock where characteristics of severe COVID-19^21^. Our findings, along with others^22,23^, provide substantial support that SARS-CoV-2 can hijack the host epigenome through DNAm.

Beta (β) values, ranging between 0 and 1, represent the estimation of methylation intensities between methylated and unmethylated alleles at each CpG site. While DNAm signatures are regarded as cell-type specific, read-level signatures have been identified that allow estimations of cell-type proportions^24^.

Prior research utilizing DNAm data from peripheral blood of SARS-CoV-2-infected and uninfected individuals was modeled using LASSO and Elasticnet to predict long-term hospitalization^17^. In this study, we expand upon their findings by applying an automated machine learning platform, which optimizes over numerous modeling and feature selection algorithms, such as ridge regressions, support vector machines, decision trees, Lasso, and Statistical Equivalent Signatures (SES) to explore DNAm footprints of severe COVID-19 disease from this large publicly available data. We identified a DNAm signature consisting of 4 methylation sites which accurately distinguished severe COVID-19 disease from healthy individuals. Our signature was then validated on an external cohort and accurately classified COVID-19-infected individuals from those diagnosed with non-COVID-related pneumonia.

## Methods

### Cohort selection and data processing

Genome-wide DNA methylation of SARS-CoV-2-infected and -uninfected patients using Illumina Infinium MethylationEPIC profiling array platform from whole blood was publicly available through NCBI Gene Expression Omnibus (GEO). GSE167202^25^ (training cohort) consisting of 525 individuals (164 COVID-19 infected, 296 COVID-19 uninfected, and 65 with other Non-COVID-19 infections) were obtained. Participants were classified by COVID-19 severity score (SS) as follows: 0. Uninfected; 1. Released from department to home; 2. Admitted to in-patient care; 3. Progressed to ICU; and 4. Death. Severe SARS-CoV-2-infected and healthy individuals were dichotomized by SS ≥ 3 or SS = 0, respectively, resulting in a training cohort of 357 individuals. GSE174818^26^ (validation cohort) consisting of 102 COVID-19 infected and 26 with non-COVID-19-related pneumonia was obtained to assess the disease specificity of our model.

Where possible, raw IDAT and clinical metadata files were downloaded from GEO. IDAT files were processed using Chip Analysis Methylation Pipeline (ChAMP)^27^ v3.13 in R 4.1.1 following developer’s recommended pipeline using the arraytype = “EPIC” flag. In short, once loaded, samples are filtered for low quality, low bead count, presence of non-CpG probes, SNP-related probes, multi-hit probes, and probes located on X or Y chromosomes. Quality control, followed by normalization, resulted in over 850,000 methylation sites per sample for analysis. While β values are simpler to interpret, M-values have been shown to be more statistically valid for algorithm-based analysis of methylation levels^28,29^; thus we utilized CpGTools^30^ v0.10.0 in Python 3.8.8 to perform this transformation.

### Machine learning modeling using JADBio

We leveraged an automated machine learning platform, JADBio^31–34^ (v1.3.32), for predictive modeling. JADBio utilizes algorithms appropriate for small-sample, high-dimensional biological data, but can be fully applied on any data represented in a tabular, 2-dimensional matrix format. The methodology of JADBio has been previously published^34,35^. To summarize: the platform selects appropriate algorithms and hyper-parameter values to try for transformation, feature selection, and predictive modeling using an artificial intelligence decision support system; the selection depends on the characteristics of the analysis task at hand. Selected algorithms are then fed into a Configuration Generator which generates the configurations (i.e., machine learning pipelines) to try. Then, the Configuration Evaluator identifies the best performing configuration (i.e., the one leading to the most predictive models on average), using performance estimation protocols such as stratified, repeated cross-validation. The choice of the protocol depends on the characteristics of the dataset. The final model is produced by training on all data using the winning configuration, so no data are lost to estimation. Predictive performance is estimated by the Bootstrap Bias Corrected Cross Validation (BBC-CV) estimation protocol^36^ which removes the optimism stemming from trying multiple configurations, conceptually equivalent to adjusting p-values in hypothesis testing. JADBio outputs a predictive model, the selected features that entered the model (biosignature), an estimate of its out-of-sample predictive performance, and their 95% confidence intervals. A recent extensive evaluation of JADBio capabilities against alternative autoML methods showed that JADBio can reduce (on average) the number of biomarkers by a factor of 4000 (to less than 20) while maintaining competitive predictive performance and accurate out-of-sample performance estimation^34^.

In the analyses in this paper, the outcome variable to predict is discrete (disease status), leading to solving a multi-class classification problem. JADBio utilized standard statistical models (ridge logistic regression), non-standard linear models (linear SVM), and non-linear models (decision trees, random forests, SVMs with non-linear kernels etc.). The feature selection algorithms of JADBio remove irrelevant but also redundant CpG sites. They select sites that are predictive in a multi-variate fashion, i.e., when used in combination. In contrast, standard differential expression analysis identifies sites that are informative when examined in isolation. It does not remove redundant sites which would result in identifying potentially thousands of CpG sites. Hence, JADBio feature selection capabilities result in more focused research on the selected few sites which carry unique predictive information.

Feature importance values are calculated by repeating the performance estimation protocol removing each feature at a time. This approach provides more detailed information compared to the commonly used alternative, the Shapely Additive explanation (SHAP) method, as SHAP values: 1. Do not show how the feature affects performances; 2. Are harder to interpret in absolute terms; and 3. Are outcome-type specific (classification, regression, etc.). Instead, the relative drop in performance we describe has a straightforward interpretation: how much performance is affected when a single feature is removed from the model.

Regarding the overwhelming amount of features from one modality, we argue that both Feature Selection algorithms that JADBio optimizes (LASSO and Statistical Equivalent Signatures [SES]^37^) are not susceptible to the high number of features from any modality. If a feature carries unique information for the target, it will be selected for.

### Statistics

Variables were summarized using descriptive statistics. Median and interquartile range were used for continuous variables, and frequencies and proportions for categorical measures. Mann–Whitney U test analysis evaluated differences between groups. Statistical analysis was performed using SPSS software version 25 (IBM, Armonk, NY). A two-sided p ≤ 0.05 was regarded as statistically significant for all tests. Uniform manifold approximation and projection (UMAP) is a nonlinear dimensionality reduction method effective at visualizing clusters or groups with superior performance compared to t-distributed stochastic neighbor embedding (t-SNE) or principal component analysis (PCA)^38^.

## Results

### Model training and feature selection

The clinical demographics of our training cohort (GSE167202) are summarized in Table 1. The cohort consisted of 460 individuals, predominantly non-Hispanic (65.9%), Caucasian (58.3%) males (59.6%) with a median age of 56 years. Patients were trichotomized into “Healthy Controls”, “Mild COVID-19” (MCV), and “Severe COVID-19” (SCV). Healthy uninfected controls (HC) tested negative for SARS-CoV-2 infection at the time of their hospital visit, while MCV and SCV tested positive but had a COVID Severity Score (SS) ≤ 2 or ≥ 3, respectively.

**Table 1:** Training cohort (GSE167202) demographics.

	Total cohort	Healthy control (HC)	Mild COVID-19 (MCV)	Severe COVID-19 (SCV)	p-value (HC v SCV)
N	460	296	118	46	
Age, years [IQR]	56 [39, 68]	59 [41, 70]	50 [37, 62]	50 [38, 62]	0.05
Gender, male (%)	274 (59.6%)	151 (51.0%)	63 (53.4%)	30 (65.2%)	0.07
**Race, n (%)**					0.001
African American	92 (20.0%)	50 (16.9%)	19 (16.1%)	6 (13.0%)	
Asian	18 (3.9%)	6 (2.0%)	8 (6.8%)	4 (8.7%)	
Caucasian	268 (58.3%)	184 (62.2%)	27 (22.9%)	19 (41.3%)	
Other	82 (17.8%)	56 (18.9%)	64 (54.2%)	17 (37.0%)	
**Ethnicity, n (%)**					
Hispanic or Latinx	157 (34.1%)	57 (19.3%)	68 (57.6%)	20 (43.5%)	< 0.001
COVID Hospitalization, Days [IQR]	NA	NA	3 [0.5, 6]	18 [10, 31]	NA
Mortality, n (%)	21 (4.6%)	7 (2.4%)	2 (1.7%)	10 (21.7%)	< 0.001

p-values were calculated using Mann–Whitney U Test and Chi-squared.

When examining SCV compared to HC, HC were predominantly Caucasian (62.2% vs 41.3%) and older (median age 59 vs 50 years). Consistent with other reports^39^, we observed an increased proportion of Asian (8.7% vs 2.0%) and Other/Multiple (37.0% vs 18.9%) races, as well as an increased Hispanic/Latinx ethnicity (43.5% vs 19.3%) in the SCV cohort compared to HC. Unsurprisingly, an increased rate of mortality (21.7%) was also observed in SCV when compared to HC (2.4%) or MCV (1.7%).

**Figure 1:**
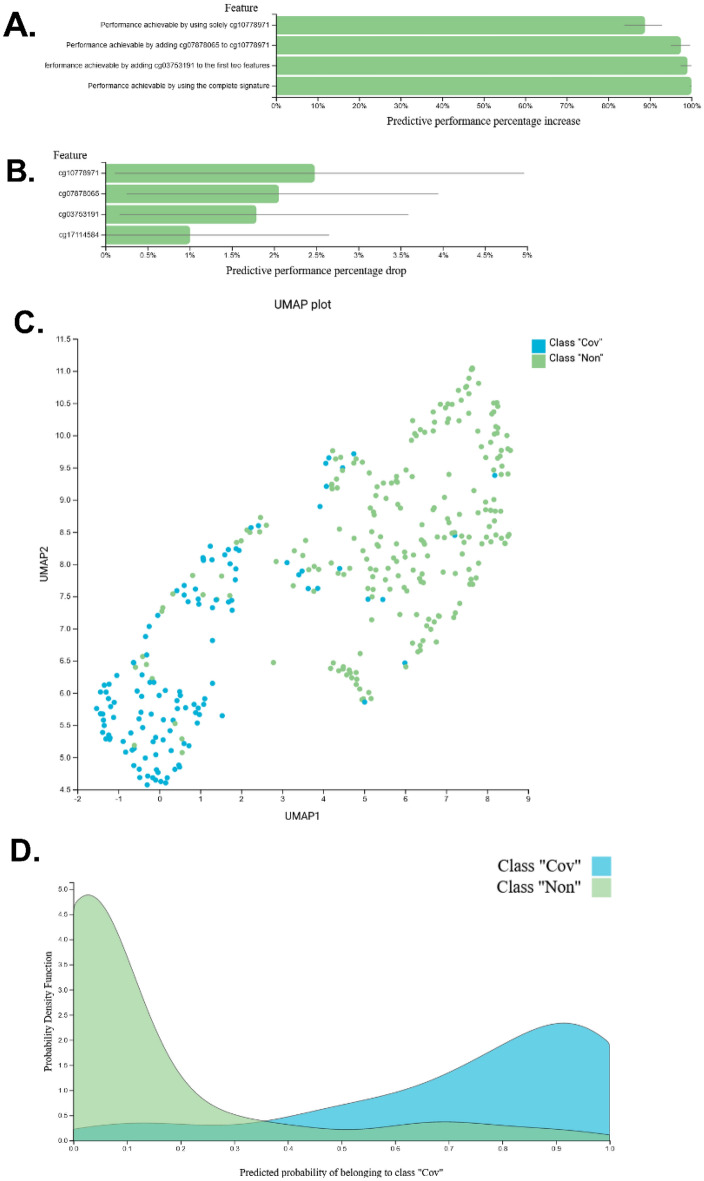
Feature selection and training performance. The selected features and performance characteristics of the training model are summarized. COVID-19 disease = “Cov”; Healthy individuals = “Non”. (**A**) The selected features consist of a single subset signature comprising 4 CpG sites shown by predictive performance increase when included in the model. (**B**) The predictive power losses by the model are reported for each CpG site removed. For some features, there may not be a noticeable loss in predictive power, however these features are included as an effort to make the final model more robust to noise. (**C**) Uniform manifold approximation and projection (UMAP) two-dimensional space projection with LASSO feature-selected CpG methylation M-values. (**D**) Separation of the predictions of the classes achieved by the model is shown in the density plot. These are the out-of-sample predictions made by the model produced by the same configuration as the final model when used for testing (e.g., during cross-validation) and not used to train the model. Well-performing models will display peaks at or close to 1 for COVID-19-infected individuals and 0 for uninfected individuals.

To identify a DNAm signature of severe COVID-19 disease, individuals within the MCV group were removed from our training cohort. JADBio selected a configuration where the feature selection step was performed by the least absolute shrinkage and selection operator (LASSO) feature selection (penalty = 1.5) and the modeling step by a classification random forest with 100 trees and with Deviance splitting criterion (minimum leaf size = 3). Supplementary Document 1 includes a textual summary of all analysis details along with the tested configuration and their performances. LASSO identified 4 unique methylation sites (summarized in Table 2) that lead to optimal predictive performance, namely cg17114584, cg07878065, cg03753191, and cg10778971, presented in order of importance. JADBio is also capable of identifying signatures that lead to models with statistically equivalent performance. However, in this case there were none, hence, these sites cannot be substituted with others and still obtain an equally good predictive performance (Fig. 1A). Some CpG sites may not seem to add predictive performance to the model; however, LASSO includes them as an effort to make the final model more robust to noise. Hence, there may still be some redundancy in the selected signature. By removing an individual site from the model, we observed a reduction in the performance relative to the performance achieved to the complete model (Fig. 1B). Uniform Manifold Approximation and Projection (UMAP)^40^ is a dimensionality reduction method based on manifold learning techniques and has been reported to be superior to PCA^41^. When the selected CpG site M-values were examined with UMAP (Fig. 1C) clustering can be observed. The predictive power of our model was assessed by contrasting the cross-validated predicted probability of belonging to a specific class against the actual class of the samples (Fig. 1D). Well-performing models are expected to provide predictions that are as close to 1 for COVID-19-infected individuals and 0 for uninfected individuals.

**Table 2:** External validation (GSE174181) demographics.

	Total cohort	Non-COVID-19 (Non)	COVID-19 (Cov)	p-value (Cov vs Non)
n	128	26	102	
Age, years [IQR]	63 [51, 75]	65 [53, 75]	62 [50, 74]	0.496
Gender, male (%)	77 (60.2%)	13 (50.0%)	64 (62.7%)	0.238
**Race, n (%)**				0.005
African American	15 (11.7%)	4 (15.4%)	11 (10.8%)	
Asian	2 (2.6%)	0 (0%)	2 (2.0%)	
Caucasian	67 (52.3%)	21 (80.8%)	46 (45.1%)	
Hispanic or Latinx	22 (17.2%)	1 (3.8%)	21 (20.6%)	
Other	22 (17.2%)	0 (0%)	22 (21.6%)	
ICU admittance, n (%)	67 (52.4%)	16 (61.5%)	51 (50.0%)	0.295

p-values were calculated using Mann–Whitney U Test and Chi-squared.

**Figure 2:**
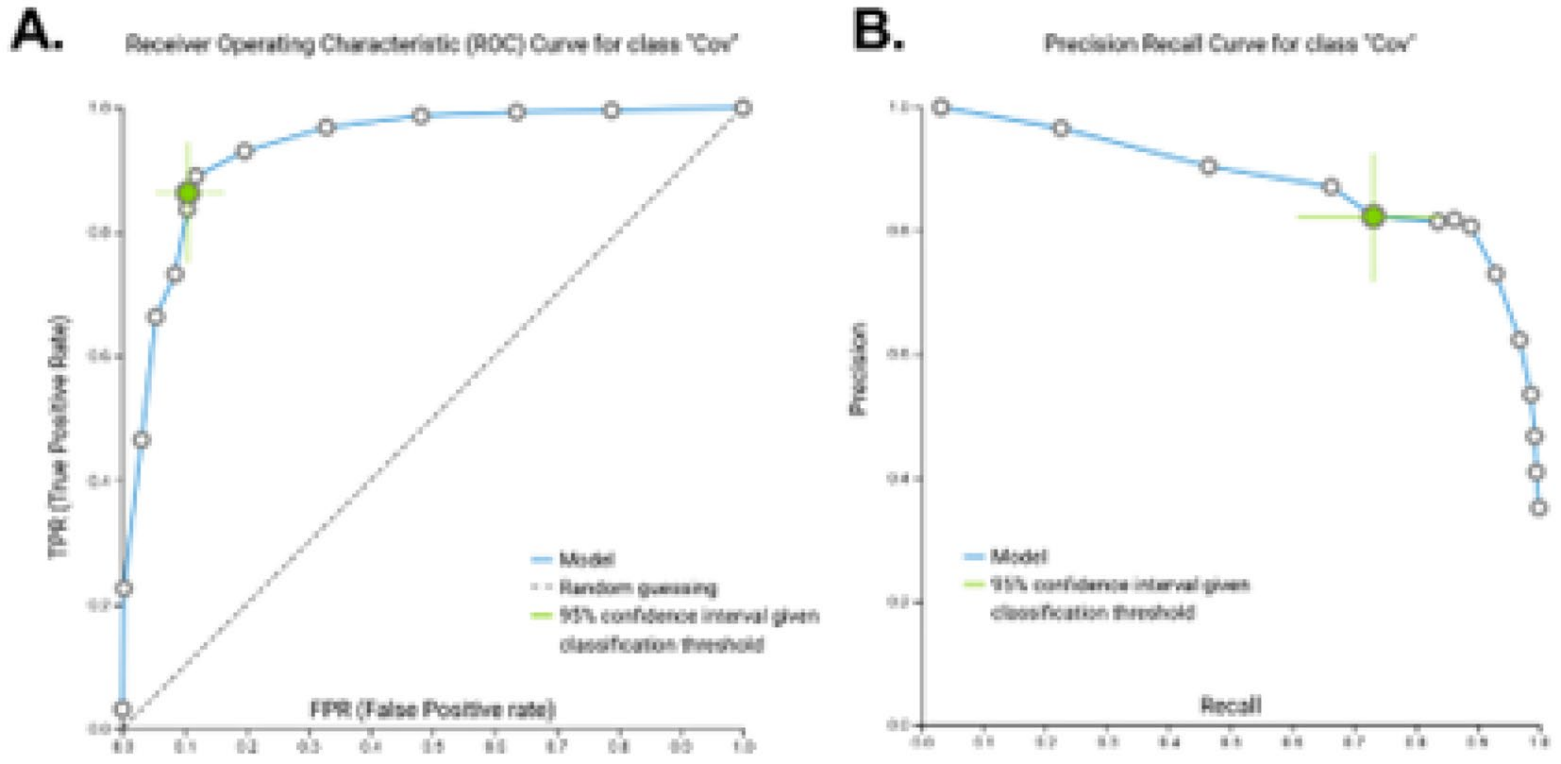
Model performance (training). Receiver-operating characteristic (ROC) and Precision-Recall curves (PRC) for the performance of the artificial intelligence-based classification model to identify patients with severe COVID-19 disease in the training (GSE167202) cohort. *AUC* area under the curve; *“Cov”* COVID-19 disease classification. Colored circle represents the optimized classification threshold to predict COVID-19 disease, while bars extending from the circle represent 95% confidence. (**A,B**) ROC (AUC = 0.933 [0.855, 0.970]) and PRC (AUC = 0.965 [0.932, 0.986]) for model training dataset.

The average area under the curve (AUC) of receiver operator characteristics (ROC) of the model was 0.933 (95% confidence interval [0.885, 0.970]), while the average area under the precision-recall curve (PRC) was 0.965 [0.932, 0.986] (Fig. 2A,B). By average AUC we mean averaging the AUC of each class against all others, over all classes. When tasked with classifying all participants based upon their severity (HC, MCV, SCV), the model produced an AUC of 0.761 [0.718, 0.803]. When demographic (age, sex, race, and ethnicity) and clinical (white blood cells, platelets, monocytes, hemoglobin, hematocrit) variables were included as features, only methylation sites were selected suggesting these other variables do not carry unique information beyond that provided by the DNAm data. Taken together, we believe our model has been well trained to accurately identify individuals with varying degrees of COVID-19 disease.

### External validation of our COVID-19 model

To determine if our model was specific to SARS-CoV-2 infection, we performed an external validation (GSE174818) on 128 adults (26 with non-COVID-19-related pneumonia [Non] and 102 COVID-19-positive [Cov]) who sought medical treatment are summarized in Table 2. Similar to the training cohort, the participants were predominantly male (60.2%) Caucasians (52.3%), although slightly older, with a median age of 63 years, and 67 (52.4%) were admitted to the ICU (16 Non and 51 Cov). There were no observable differences in age, gender, or ICU-admittance between the Cov and Non groups, however racial disparities were observed (p = 0.005). 17 of 26 from out non-COVID-19 group were diagnosed with pneumonia, 7 of which were admitted to the ICU. GSE174818 was selected as an external validation to assess the predictive power of our model to discern COVID-19 disease from non-COVID-19 with similar pathology.

**Figure 3:**
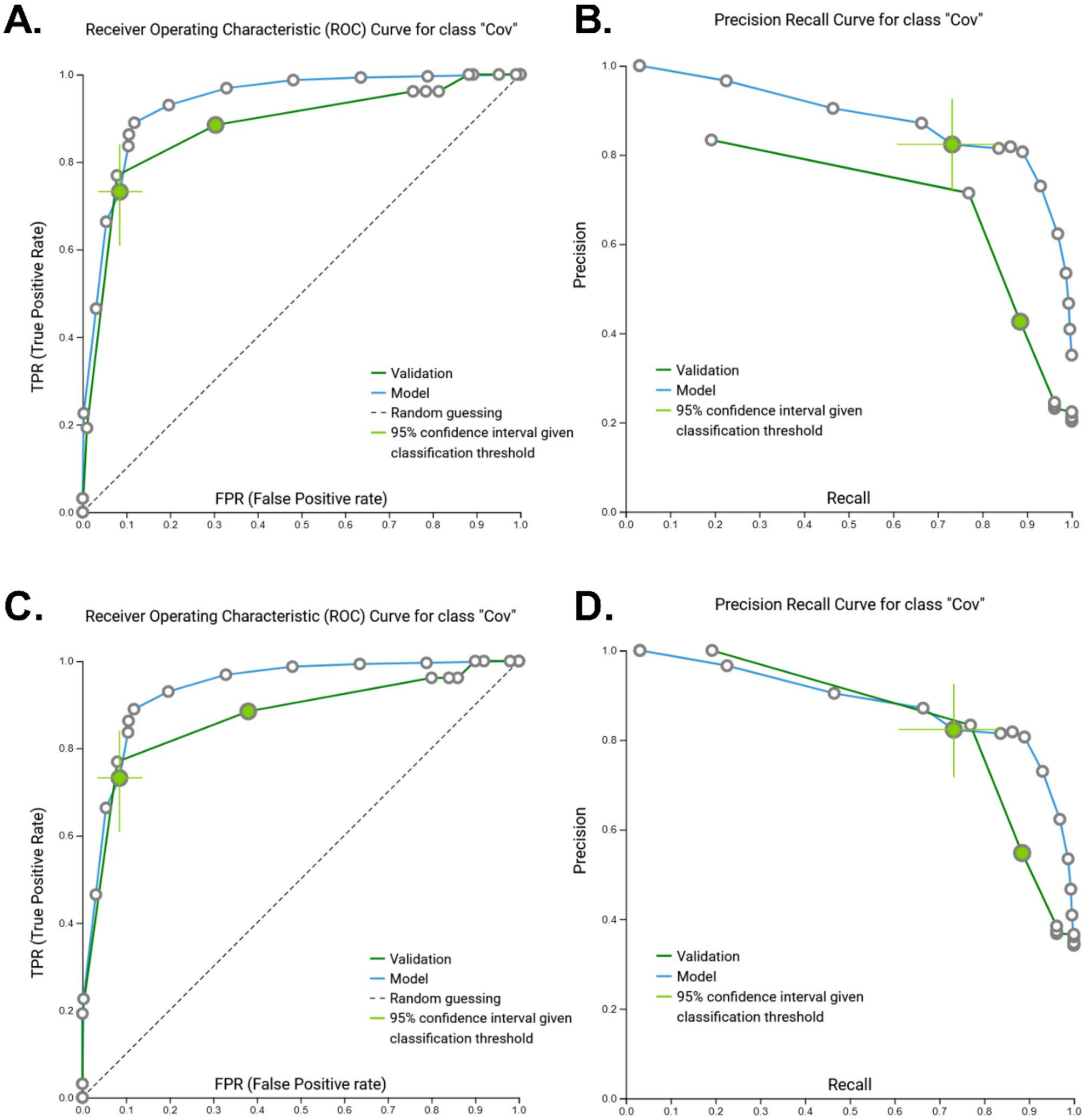
Model performance (external validation). Receiver-operating characteristic (ROC) and Precision-Recall curves (PRC) for the validation of the artificial intelligence-based classification model to identify patients with COVID-19 disease. *AUC* area under the curve; *“Cov”* COVID-19 disease classification. Colored circle represents the optimized classification threshold to predict COVID-19 disease, while bars extending from the circle represent 95% confidence. (**A,B**) AUC-ROC = 0.901 and AUC-PRC = 0.965 for the complete external validation dataset. (**C,D**) AUC-ROC = 0.898 and AUC-PRC = 0.864 when non-ICU COVID-19 patients were removed from the external validation dataset.

When applied to our external validation, this model produced an AUC-ROC of 0.901 with an AUC-PRC of 0.748 (Fig. 3A,B). Despite the model’s high performance classifying mild COVID-19 diseased participants within the training set, we removed non-ICU COVID-19 patients from the validation dataset to explore if the model was more precise at classifying these severe COVID-19 patients. Under these conditions, the model produced a slightly lower AUC-ROC of 0.898 while gaining in precision-recall AUC (AUC-PRC = 0.864, Fig. 3C,D) suggesting that our model is more adequately trained at detecting severe COVID-19 cases than mild. Interestingly, our model was successful at identifying both healthy controls and hospitalized individuals without SARS-CoV-2 infection-associated pneumonia from those suffering from COVID-19 suggesting our model may be unique to this condition.

### Examination of CpG methylation β values

**Figure 4:**
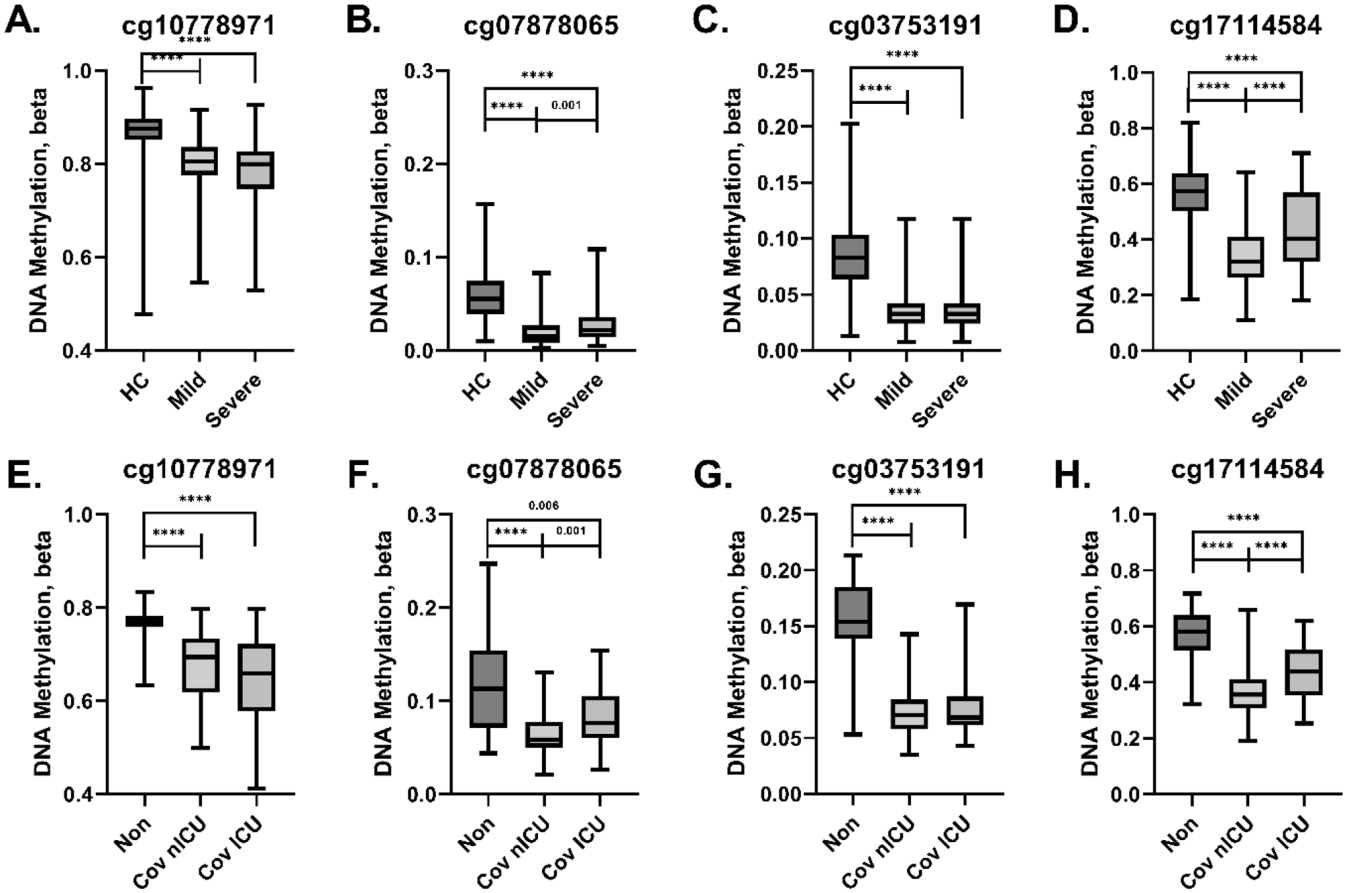
Methylation frequencies observed in training and validation datasets. Box and whisker plots are shown for the frequency of methylation (Beta [β] values) observed for the 4 CpG sites within the training (**A–D**) and validation (**E–H**) cohorts. “Non” indicates non-COVID-19 hospitalized patients; *“Cov nICU”* COVID-19 hospitalized without ICU admittance; and “Cov ICU” indicates COVID-19 hospitalization within the ICU. p-values were obtained by Mann–Whitney U Test. ****Indicates p-values < 0.001.

β values are the calculated frequency of cells represented within a sample which are methylated at a given CpG site. Given the clearer interpretation of β values compared to M values, we elected to compare β values at the four CpG sites selected by our model between the group comprising our training and validation cohorts (Fig. 4).

Within the training cohort, β values were significantly higher in our healthy controls compared to patients with mild and severe COVID-19 (p < 0.001 for all four CpG sites examined, Fig. 4A–D). β values were significantly higher in the severe group compared to the mild group at cg07878065 (p = 0.001, Fig. 4B) and cg17114584 (p < 0.001, Fig. 4D), while no significance was observed at cg10778971 and cg03753191 (Fig. 4A,C, respectively).

Within the validation cohort, β values were significantly higher in the patients hospitalized for non-COVID-19 conditions (“Non”) compared to patients with COVID-19 with or without admittance to the ICU (0.006 ≤ p < 0.001, Fig. 4E–H). Similar to the training cohort, there were significant increases in β values at cg07878065 (p = 0.001) and cg17114584 (p < 0.001) in COVID-19 patients admitted to the ICU (“Cov ICU”) compared to COVID-19 patients who were not admitted to the ICU (“Cov nICU”, Fig. 4F,H). Likewise, no significant differences were observed at cg10778971 and cg03753191 between Cov ICU and Cov nICU (Fig. 4E,G). The trends consistent between these two cohorts suggest an association between the demethylation of these CpG sites and COVID-19 pathology.

## Discussion

To the best of our knowledge, we are the first to report on cg07878065 in the context of SARS-CoV-2 infection and the first to explore the other associated genes via DNAm. In this study, we identified 4 methylation sites (Table 3) which carried the power to accurately classify individuals with severe COVID-19 disease. Except for cg07878065, the associated genes of these selected sites, have been well studied in and out of context of SARS-CoV-2.

**Table 3:** Genome association of CpG sites.

	Location	Gene	Reported exposure	Previous studies	COVID-specific studies
cg10778971	chr14:94,577,101	IFI27	Age, pregnancy-associated hypertension, cancer	^51–53^	^44,54^
cg07878065	chr18:2,641,871	–	Maternal dysglycemia, mixed connective tissue disease	^55,56^	–
cg03753191	chr13:43,566,902	EPSTI1	Age, primary Sjorgren’s syndrome, rheumatoid arthritis	^51,52^	^45,46^
cg17114584	chr11:613,792	IRF7	Age, primary Sjorgren’s syndrome, smoking, multiple sclerosis	^51,57,58^	^59–61^

*IFI27* interferon alpha inducible protein 27, *EPSTI1* epithelial stromal interaction 1, *IRF7* interferon regulatory factor 7.

Interferon Alpha Inducible Protein 27 (IFI27) is a protein encoding gene which plays a role in the innate immune system and interferon gamma signaling^42^ and has been reported as a biomarker of influenza infection^43^. Within the context of SARS-CoV-2 infection, IFI27 RNA transcripts have been identified as a biomarker for infection within peripheral blood^44^ and upper airway samples^32^, has been shown to be the strongest upregulated gene from scRNA-seq data, and transcriptionally can be found upregulated in bronchoalveolar lavage (BAL)^45^. Consistent with these observations, we observed a significant decrease in DNA methylation at IFI27-associated cg10778971 in both our training and validation cohorts (Fig. 4A,E).

Epithelial Stromal Interaction 1 (EPSI1) encodes for a protein which plays a role in M1 macrophage polarization and is required for proper regulation of gene expression during M1 and M2 macrophage differentiation. EPSI1 was found upregulated in BAL and peripheral blood mononuclear cells in patients with COVID-19 by scRNA-seq^45^ and a 6 gene panel which included EPSI1 was shown to be capable of classifying COVID-19 disease by logistic regression^46^. These factors, in combination with monocyte dysfunction reported in severe COVID-19 disease^47^, and the reduced methylation profile observed at cg03753191 in this study (Fig. 4C,G) provide strong support for the inclusion of cg03753191 in our model.

Interferon Regulatory Factor 7 (IRF7) is a multifunctional transcription factor that plays a role in the interferon-induced antiviral innate immune response. Due to the dysregulated type I interferon response during SAR-CoV-2 infection, IRF7 and other members of its family of transcription factors have been widely studied^48^. Studies involving supplemental interferon administration initially were optimistic, however recently interferon β-1a was shown not to be superior to regular treatment alone^49^. Like the other CpG sites examined in our study, we saw decreased methylation at the IRF7-associated site, cg17114584 (Fig. 4D,H), which implies a potential for increased transcription of IRF7. SARS-CoV-2 S protein-induced expression of SOCS3, which can target IRF7 for degradation^50^, may partially explain this disconnect.

This study was limited by the availability of DNAm data derived from Illumina’s MethylationEPIC profiling array on DNA extracted from whole blood. The greater abundance of CpG sites compared to clinical features may have reduced the likelihood of these features being selected during feature selection. Future studies would benefit from subsetting peripheral blood mononuclear cells (PBMCs) to elucidate the epigenetic mechanisms employed by SARS-CoV-2 within specific cell populations. Furthermore, the M-value transformation used within this study may have biased which model was ultimately selected. Recruitment for participants began in March 2020 and within a few months, many hospitals were discharging all but the most severe cases due to capacity limitations. This casts doubt on if severity score 1 and 2 (‘discharged from ER to home’ and ‘admitted but not progressed to ICU’, respectively) are based on disease severity or bed availability. The lack of available matched pre-infection data limits this study’s ability to identify healthy individuals at increased risk of developing severe COVID. “Long COVID” is a recognized but unexplained complication with COVID-19 disease which can last for months following initial resolution of symptoms. DNAm profiling may serve as a valuable tool in uncovering the underlying mechanisms leading to this condition, understanding its unique pathogenesis, and provide potential approaches to treatment and prevention.

In conclusion, our analysis revealed a 4 CpG methylation signature with the power to classify individuals with COVID-19 disease by leveraging JADBio AutoML platform. These findings enhance our understanding of COVID-19 disease at the methylation level and may serve to provide guidance for future COVID-19-related studies.

## Supplementary Information

Supplementary Information.

## Data Availability

The data used within this study is available through NCBI GEO database with the Accession Numbers GSE167202 and GSE174818.
