# Supplementary material for: A machine learning approach utilizing DNA methylation as an accurate classifier of COVID-19 disease severity
Source: Sci Rep. 2022 Oct 19;12:17480. doi: 10.1038/s41598-022-22201-4 (PMC9580434; doi:10.1038/s41598-022-22201-4)
Supplement: Supplementary file 1 — Supplementary Information. [file 41598_2022_22201_MOESM1_ESM.pdf]

JADBio Description of Performed Analysis

Visit analysis

Setup

JADBio version **1.4.0** ran on dataset **GSE167202.MVals.Sev.Non.Train** with **357** samples and **846291** features to create a predictive model for outcome named **Group**. The outcome was discrete leading to a **classification** modeling.

The preferences of the analysis were set to **true** for feature selection and **false** for full feature models tried.
The **AUC** metric was used to optimize for the best model.
The maximum number of features to select was set to **5**.
The effort to spend on tuning the algorithms were set to **Typical**.
The number of CPU cores to use for the analysis was set to **6**.
The execution time was **22:47:54**.

Configuration Space

JADBio's AI decide to try the following algorithms and tuning hyper-parameter values:

Algorithm Type	Algorithm	Hyper-parameter	Set of Values
Preprocessing	Contant Removal		
	Standardization		
Feature Selection	Test-Budgeted Statistically Equivalent Signature (SES)	alpha	0.05, 0.01
		maxk	2, 3
	LASSO	penalties	1.0, 1.5, 0.5
Modeling	Linear Support Vector Machines	costs	10.0, 1.0, 0.1, 0.01
	Polynomial Support Vector Machines	gammas	10.0, 1.0, 0.01, 0.1
		costs	10.0, 1.0, 0.1, 0.01
		degrees	3, 2
	RBF Support Vector Machines	gammas	10.0, 1.0, 0.01, 0.1
		costs	10.0, 1.0, 0.1, 0.01
	Logistic Regression	lambdas	1.0, 0.1, 10.0
	Random Forests	min leaf sizes	3, 2, 4
		vars to split	1.291 sqrt (nvars), 0.816 sqrt (nvars), 1.154 sqrt (nvars), 1.0 sqrt (nvars)
		splits to perform	1.0
		ntrees	100, 500
	Decision Tree	min leaf sizes	2, 4, 3
		vars to split	nvars // 1.0
		splits to perform	1.0
		alphas	0.01, 0.05

Leading to **596** combinations and corresponding configurations (machine learning pipelines) to try. For the full configurations tested see the Appendix.

Configuration Estimation Protocol

JADBio's AI system decided to estimate the out-of-sample performance of the models produced by each configuration using **Repeated 10-fold CV without dropping (max. repeats = 20).** Overall, 596 configurations × 20 repeats × 10 folds = 119200 models were set out to train. Out of those, only 5960 models were eventually trained, as JADBio stopped all configuration evaluations when it deemed that no sufficient progress was made. JADBio **did not use** the Early Dropping criterion (see [1]) to stop computations early on configurations that did not seem promising.

A detailed report of the above is available at Visit analysis

JADBio Results Summary

Overview

A result summary is presented for analysis optimized for Performance. The model is produced by applying the algorithms in sequence (configuration) on the training data:

Preprocessing	Feature Selection	Predictive algorithm
Constant Removal, Standardization	LASSO Feature Selection (penalty=1.5)	Classification Random Forests training 100 trees with Deviance splitting criterion, minimum leaf size = 3, and variables to split = 1.154 sqrt (nvars)

The **Area Under The Curve** is **0.933** with 95% confidence interval being [**0.885,0.970**].
The **Average Precision (a.k.a. Area Under the Precision-Recall curve)** is **0.965** with 95% confidence interval being [**0.932,0.986**].

The Area Under the ROC Curve is shown in the figure below:

Feature Selection

There were **4** features selected out of the **846291** available.

The selected features consist of the following subset called a signature. **There was a single signature identified.** The first signature identified by the system is the set: **cg17114584, cg07878065, cg03753191, cg10778971** in order of importance. The following features cannot be substituted with others and still obtain an equal predictive performance: **cg17114584, cg07878065, cg03753191, cg10778971**.

The performance achieved by adding each feature in sequence to the model relative to the performance of the final model with all selected features is shown below. The features are added in order of importance:

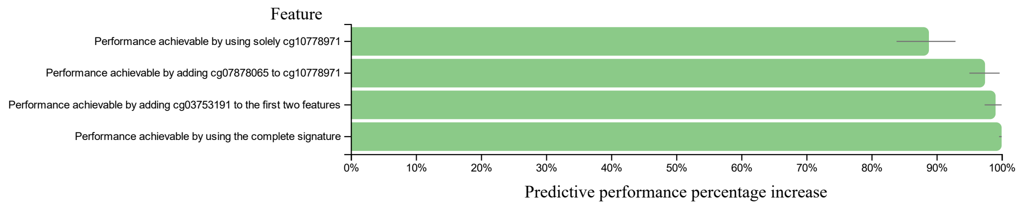

Some features may not seem to add predictive performance to the model; however, the feature selection algorithms include them as an effort to make the final model more robust to noise. The performances achieved by a model that contains all features except one, relative to the performance achieved when the feature is removed is shown below:

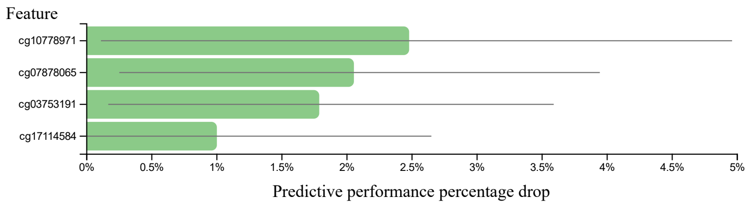

For some features there is no noticeable drop in performance when they are removed because they carry predictive information that is shared by other features selected.

The separation of the predictions of the classes achieved by the model is shown in the box-plots below. These are the out-of-sample predictions made by model produced by the same configuration as the final model when the sample was used for testing (e.g., during cross-validation) and was not used to train the model.

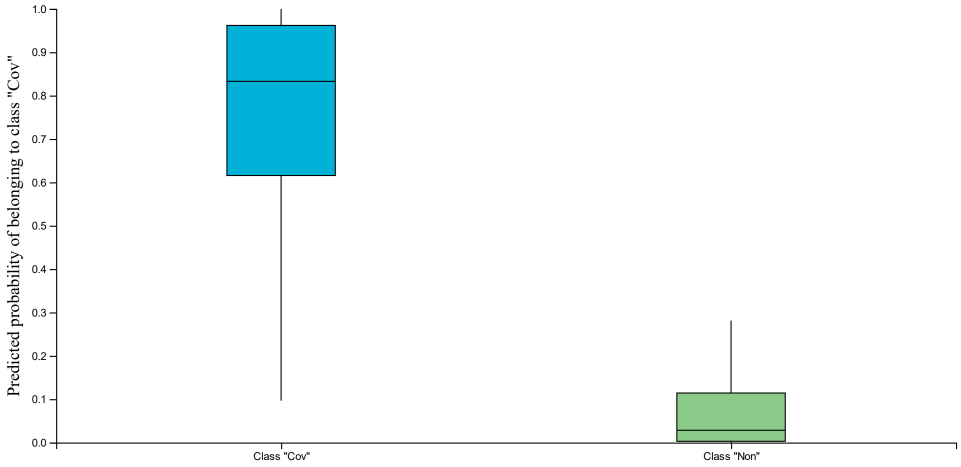

Appendix

Configuration	Preprocessing	Name	Hyperparams	Name	Hyperparams	Performance (unadjusted)	Time (miliseconds)	Dropped
1	Constant Removal, Standardization	Test-Budgeted Statistically Equivalent Signature (SES) algorithm	maxK = 2, alpha = 0.01, budget = 3 * nvars	Support Vector Machines (SVM) of type C-SVC	kernel = 'Polynomial Kernel', cost = 0.01, gamma = 0.01, degree = 3	0.9070825097039933	00:00:41.41012	false
2	Constant Removal, Standardization	LASSO Feature Selection	penalty = 1.0	Support Vector Machines (SVM) of type C-SVC	kernel = 'Radial Basis Function Kernel', cost = 1.0, gamma = 0.01	0.9146638030909131	00:03:40.220412	false
3	Constant Removal, Standardization	LASSO Feature Selection	penalty = 1.5	Support Vector Machines (SVM) of type C-SVC	kernel = 'Polynomial Kernel', cost = 0.1, gamma = 10.0, degree = 2	0.5527649744401664	00:03:39.219719	false
4	Constant Removal, Standardization	Test-Budgeted Statistically Equivalent Signature (SES) algorithm	maxK = 2, alpha = 0.01, budget = 3 * nvars	Support Vector Machines (SVM) of type C-SVC	kernel = 'Polynomial Kernel', cost = 0.1, gamma = 10.0, degree = 3	0.778636538227331	00:00:41.41013	false
5	Constant Removal, Standardization	LASSO Feature Selection	penalty = 1.0	Support Vector Machines (SVM) of type C-SVC	kernel = 'Polynomial Kernel', cost = 0.1, gamma = 0.1, degree = 3	0.6827196318884299	00:03:40.220410	false
6	Constant Removal, Standardization	LASSO Feature Selection	penalty = 1.5	Support Vector Machines (SVM) of type C-SVC	kernel = 'Polynomial Kernel', cost = 1.0, gamma = 0.1, degree = 2	0.6932495963825888	00:03:39.219719	false
7	Constant Removal, Standardization	Test-Budgeted Statistically Equivalent Signature (SES) algorithm	maxK = 3, alpha = 0.01, budget = 3 * nvars	Classification Random Forests with Deviance splitting criterion	ntrees = 100, minimum leaf size = 4	0.8857520711740662	00:00:41.41218	false
8	Constant Removal, Standardization	LASSO Feature Selection	penalty = 1.5	Classification Random Forests with	ntrees = 500, minimum leaf size = 4	0.9332489406018819	00:03:39.219785	false

Configuration	Preprocessing	Name	Hyperparams	Name	Hyperparams	Performance (unadjusted)	Time (miliseconds)	Dropped
				Deviance splitting criterion				
9	Constant Removal, Standardization	LASSO Feature Selection	penalty = 1.5	Ridge Logistic Regression	lambda = 0.1	0.9220065952802525	00:03:39.219719	false
10	Constant Removal, Standardization	Test-Budgeted Statistically Equivalent Signature (SES) algorithm	maxK = 2, alpha = 0.01, budget = 3 * nvars	Classification Random Forests with Deviance splitting criterion	ntrees = 100, minimum leaf size = 4	0.8745347079746056	00:00:40.40991	false
11	Constant Removal, Standardization	Test-Budgeted Statistically Equivalent Signature (SES) algorithm	maxK = 3, alpha = 0.05, budget = 3 * nvars	Support Vector Machines (SVM) of type C-SVC	kernel = 'Polynomial Kernel', cost = 1.0, gamma = 0.01, degree = 2	0.8569511974243432	00:01:04.64125	false
12	Constant Removal, Standardization	Test-Budgeted Statistically Equivalent Signature (SES) algorithm	maxK = 2, alpha = 0.01, budget = 3 * nvars	Classification Random Forests with Deviance splitting criterion	ntrees = 100, minimum leaf size = 2	0.8880449615743733	00:00:40.40999	false
13	Constant Removal, Standardization	LASSO Feature Selection	penalty = 1.0	Classification Random Forests with Deviance splitting criterion	ntrees = 500, minimum leaf size = 2	0.9332309847015732	00:03:40.220491	false
14	Constant Removal, Standardization	LASSO Feature Selection	penalty = 1.5	Classification Random Forests with Deviance splitting criterion	ntrees = 500, minimum leaf size = 4	0.933425376839699	00:03:39.219757	false
15	Constant Removal, Standardization	Test-Budgeted Statistically Equivalent Signature (SES) algorithm	maxK = 3, alpha = 0.01, budget = 3 * nvars	Support Vector Machines (SVM) of type C-SVC	kernel = 'Radial Basis Function Kernel', cost = 10.0, gamma = 0.01	0.7477242848086837	00:00:41.41206	false
16	Constant Removal, Standardization	LASSO Feature Selection	penalty = 1.0	Classification Decision Tree with Deviance splitting criterion	minimum leaf size = 4, alpha = 0.01	0.8924982200237955	00:03:40.220415	false
17	Constant Removal, Standardization	LASSO Feature Selection	penalty = 1.0	Support Vector Machines (SVM) of type C-SVC	kernel = 'Polynomial Kernel', cost = 0.01, gamma = 1.0, degree = 3	0.8898553847402951	00:03:40.220410	false
18	Constant Removal, Standardization	LASSO Feature Selection	penalty = 1.5	Support Vector Machines (SVM) of type C-SVC	kernel = 'Polynomial Kernel', cost = 10.0, gamma = 10.0, degree = 3	0.8004143128823435	00:03:39.219719	false
19	Constant Removal, Standardization	Test-Budgeted Statistically Equivalent Signature (SES) algorithm	maxK = 3, alpha = 0.05, budget = 3 * nvars	Support Vector Machines (SVM) of type C-SVC	kernel = 'Radial Basis Function Kernel', cost = 1.0, gamma = 10.0	0.8150069949942071	00:01:04.64128	false
20	Constant Removal, Standardization	LASSO Feature Selection	penalty = 1.5	Support Vector Machines (SVM) of type	kernel = 'Polynomial Kernel', cost = 10.0, gamma =	0.6932495963825888	00:03:39.219719	false

Configuration	Preprocessing	Name	Hyperparams	Name	Hyperparams	Performance (unadjusted)	Time (miliseconds)	Dropped
				C-SVC	0.1, degree = 2			
21	Constant Removal, Standardization	Test-Budgeted Statistically Equivalent Signature (SES) algorithm	maxK = 2, alpha = 0.05, budget = 3 * nvars	Classification Random Forests with Deviance splitting criterion	ntrees = 500, minimum leaf size = 2	0.8868376224514332	00:00:33.33266	false
22	Constant Removal, Standardization	Test-Budgeted Statistically Equivalent Signature (SES) algorithm	maxK = 3, alpha = 0.01, budget = 3 * nvars	Support Vector Machines (SVM) of type C-SVC	kernel = 'Polynomial Kernel', cost = 0.01, gamma = 0.1, degree = 2	0.8978043837378876	00:00:41.41205	false
23	Constant Removal, Standardization	LASSO Feature Selection	penalty = 1.5	Support Vector Machines (SVM) of type C-SVC	kernel = 'Linear Kernel', cost = 0.1	0.9253202395785518	00:03:39.219719	false
24	Constant Removal, Standardization	Test-Budgeted Statistically Equivalent Signature (SES) algorithm	maxK = 2, alpha = 0.01, budget = 3 * nvars	Support Vector Machines (SVM) of type C-SVC	kernel = 'Polynomial Kernel', cost = 10.0, gamma = 0.1, degree = 3	0.7463830571247452	00:00:41.41012	false
25	Constant Removal, Standardization	Test-Budgeted Statistically Equivalent Signature (SES) algorithm	maxK = 2, alpha = 0.01, budget = 3 * nvars	Support Vector Machines (SVM) of type C-SVC	kernel = 'Polynomial Kernel', cost = 0.01, gamma = 10.0, degree = 3	0.7988720571840777	00:00:41.41013	false
26	Constant Removal, Standardization	Test-Budgeted Statistically Equivalent Signature (SES) algorithm	maxK = 2, alpha = 0.05, budget = 3 * nvars	Classification Random Forests with Deviance splitting criterion	ntrees = 100, minimum leaf size = 2	0.8794128576737275	00:00:33.33198	false
27	Constant Removal, Standardization	LASSO Feature Selection	penalty = 1.0	Classification Decision Tree with Deviance splitting criterion	minimum leaf size = 2, alpha = 0.05	0.8422263833069459	00:03:40.220416	false
28	Constant Removal, Standardization	LASSO Feature Selection	penalty = 0.5	Support Vector Machines (SVM) of type C-SVC	kernel = 'Linear Kernel', cost = 10.0	0.9249650250289635	00:03:40.220184	false
29	Constant Removal, Standardization	Test-Budgeted Statistically Equivalent Signature (SES) algorithm	maxK = 3, alpha = 0.01, budget = 3 * nvars	Support Vector Machines (SVM) of type C-SVC	kernel = 'Polynomial Kernel', cost = 10.0, gamma = 1.0, degree = 2	0.7585610609907285	00:00:41.41205	false
30	Constant Removal, Standardization	LASSO Feature Selection	penalty = 1.5	Support Vector Machines (SVM) of type C-SVC	kernel = 'Polynomial Kernel', cost = 0.1, gamma = 0.01, degree = 3	0.8981599886331344	00:03:39.219719	false
31	Constant Removal, Standardization	Test-Budgeted Statistically Equivalent Signature (SES) algorithm	maxK = 2, alpha = 0.01, budget = 3 * nvars	Classification Random Forests with Deviance splitting criterion	ntrees = 100, minimum leaf size = 3	0.8776988498855506	00:00:41.41015	false
32	Constant Removal,	LASSO Feature	penalty = 1.0	Classification Random	ntrees = 100, minimum leaf	0.93945504623254	00:03:40.220423	false

Configuration	Preprocessing	Name	Hyperparams	Name	Hyperparams	Performance (unadjusted)	Time (miliseconds)	Dropped
	Standardization	Selection		Forests with Deviance splitting criterion	size = 3			
33	Constant Removal, Standardization	Test-Budgeted Statistically Equivalent Signature (SES) algorithm	maxK = 3, alpha = 0.05, budget = 3 * nvars	Support Vector Machines (SVM) of type C-SVC	kernel = 'Radial Basis Function Kernel', cost = 0.1, gamma = 0.1	0.8743883283525227	00:01:04.64127	false
34	Constant Removal, Standardization	LASSO Feature Selection	penalty = 1.5	Classification Random Forests with Deviance splitting criterion	ntrees = 500, minimum leaf size = 2	0.934296628350337	00:03:39.219789	false
35	Constant Removal, Standardization	Test-Budgeted Statistically Equivalent Signature (SES) algorithm	maxK = 2, alpha = 0.01, budget = 3 * nvars	Support Vector Machines (SVM) of type C-SVC	kernel = 'Radial Basis Function Kernel', cost = 0.1, gamma = 0.01	0.8425298770567313	00:00:41.41006	false
36	Constant Removal, Standardization	Test-Budgeted Statistically Equivalent Signature (SES) algorithm	maxK = 3, alpha = 0.01, budget = 3 * nvars	Support Vector Machines (SVM) of type C-SVC	kernel = 'Radial Basis Function Kernel', cost = 1.0, gamma = 0.1	0.862127571206855	00:00:41.41206	false
37	Constant Removal, Standardization	LASSO Feature Selection	penalty = 0.5	Support Vector Machines (SVM) of type C-SVC	kernel = 'Polynomial Kernel', cost = 0.1, gamma = 0.01, degree = 2	0.9129029538236701	00:03:40.220180	false
38	Constant Removal, Standardization	LASSO Feature Selection	penalty = 1.0	Classification Decision Tree with Deviance splitting criterion	minimum leaf size = 2, alpha = 0.01	0.8853435744420399	00:03:40.220416	false
39	Constant Removal, Standardization	Test-Budgeted Statistically Equivalent Signature (SES) algorithm	maxK = 3, alpha = 0.01, budget = 3 * nvars	Support Vector Machines (SVM) of type C-SVC	kernel = 'Radial Basis Function Kernel', cost = 10.0, gamma = 10.0	0.8146517804446193	00:00:41.41208	false
40	Constant Removal, Standardization	LASSO Feature Selection	penalty = 1.5	Support Vector Machines (SVM) of type C-SVC	kernel = 'Polynomial Kernel', cost = 10.0, gamma = 0.01, degree = 3	0.9159218871495085	00:03:39.219719	false
41	Constant Removal, Standardization	LASSO Feature Selection	penalty = 0.5	Support Vector Machines (SVM) of type C-SVC	kernel = 'Polynomial Kernel', cost = 1.0, gamma = 1.0, degree = 2	0.5	00:03:40.220180	false
42	Constant Removal, Standardization	Test-Budgeted Statistically Equivalent Signature (SES) algorithm	maxK = 2, alpha = 0.01, budget = 3 * nvars	Classification Random Forests with Deviance splitting criterion	ntrees = 100, minimum leaf size = 2	0.8794128576737275	00:00:40.40989	false
43	Constant Removal, Standardization	LASSO Feature Selection	penalty = 0.5	Support Vector Machines (SVM) of type C-SVC	kernel = 'Polynomial Kernel', cost = 0.01, gamma = 0.1, degree = 3	0.9277528737247406	00:03:40.220179	false

Configuration	Preprocessing	Name	Hyperparams	Name	Hyperparams	Performance (unadjusted)	Time (miliseconds)	Dropped
44	Constant Removal, Standardization	LASSO Feature Selection	penalty = 0.5	Support Vector Machines (SVM) of type C-SVC	kernel = 'Radial Basis Function Kernel', cost = 0.1, gamma = 10.0	0.8688774283403441	00:03:40.220183	false
45	Constant Removal, Standardization	LASSO Feature Selection	penalty = 1.5	Support Vector Machines (SVM) of type C-SVC	kernel = 'Polynomial Kernel', cost = 1.0, gamma = 0.01, degree = 3	0.9159218871495085	00:03:39.219719	false
46	Constant Removal, Standardization	Test-Budgeted Statistically Equivalent Signature (SES) algorithm	maxK = 3, alpha = 0.05, budget = 3 * nvars	Classification Random Forests with Deviance splitting criterion	ntrees = 500, minimum leaf size = 3	0.8890407333501961	00:01:04.64201	false
47	Constant Removal, Standardization	LASSO Feature Selection	penalty = 1.0	Classification Decision Tree with Deviance splitting criterion	minimum leaf size = 4, alpha = 0.05	0.8577490639511101	00:03:40.220415	false
48	Constant Removal, Standardization	LASSO Feature Selection	penalty = 1.5	Classification Random Forests with Deviance splitting criterion	ntrees = 100, minimum leaf size = 4	0.9384104812493559	00:03:39.219732	false
49	Constant Removal, Standardization	Test-Budgeted Statistically Equivalent Signature (SES) algorithm	maxK = 2, alpha = 0.05, budget = 3 * nvars	Support Vector Machines (SVM) of type C-SVC	kernel = 'Polynomial Kernel', cost = 10.0, gamma = 0.1, degree = 3	0.7463830571247452	00:00:33.33190	false
50	Constant Removal, Standardization	Test-Budgeted Statistically Equivalent Signature (SES) algorithm	maxK = 2, alpha = 0.01, budget = 3 * nvars	Support Vector Machines (SVM) of type C-SVC	kernel = 'Radial Basis Function Kernel', cost = 1.0, gamma = 0.1	0.8611532684422709	00:00:40.40984	false
51	Constant Removal, Standardization	LASSO Feature Selection	penalty = 1.0	Classification Random Forests with Deviance splitting criterion	ntrees = 100, minimum leaf size = 2	0.9337548285758005	00:03:40.220423	false
52	Constant Removal, Standardization	LASSO Feature Selection	penalty = 0.5	Support Vector Machines (SVM) of type C-SVC	kernel = 'Polynomial Kernel', cost = 0.1, gamma = 1.0, degree = 2	0.6924739795583786	00:03:40.220180	false
53	Constant Removal, Standardization	LASSO Feature Selection	penalty = 1.5	Classification Random Forests with Deviance splitting criterion	ntrees = 500, minimum leaf size = 2	0.9346647243066679	00:03:39.219760	false
54	Constant Removal, Standardization	Test-Budgeted Statistically Equivalent Signature (SES) algorithm	maxK = 2, alpha = 0.05, budget = 3 * nvars	Classification Random Forests with Deviance splitting criterion	ntrees = 500, minimum leaf size = 3	0.8864234657073533	00:00:33.33267	false
55	Constant Removal, Standardization	Test-Budgeted Statistically Equivalent Signature	maxK = 3, alpha = 0.01, budget = 3 * nvars	Classification Random Forests with Deviance splitting	ntrees = 500, minimum leaf size = 3	0.8890407333501961	00:00:41.41281	false

Configuration	Preprocessing	Name	Hyperparams	Name	Hyperparams	Performance (unadjusted)	Time (miliseconds)	Dropped
		(SES) algorithm		criterion				
56	Constant Removal, Standardization	Test-Budgeted Statistically Equivalent Signature (SES) algorithm	maxK = 2, alpha = 0.05, budget = 3 * nvars	Support Vector Machines (SVM) of type C-SVC	kernel = 'Radial Basis Function Kernel', cost = 0.01, gamma = 1.0	0.874969162692948	00:00:33.33193	false
57	Constant Removal, Standardization	Test-Budgeted Statistically Equivalent Signature (SES) algorithm	maxK = 3, alpha = 0.01, budget = 3 * nvars	Support Vector Machines (SVM) of type C-SVC	kernel = 'Radial Basis Function Kernel', cost = 0.1, gamma = 0.1	0.8743883283525227	00:00:41.41206	false
58	Constant Removal, Standardization	LASSO Feature Selection	penalty = 0.5	Classification Decision Tree with Deviance splitting criterion	minimum leaf size = 3, alpha = 0.01	0.8953471578151886	00:03:40.220183	false
59	Constant Removal, Standardization	Test-Budgeted Statistically Equivalent Signature (SES) algorithm	maxK = 3, alpha = 0.05, budget = 3 * nvars	Classification Random Forests with Deviance splitting criterion	ntrees = 500, minimum leaf size = 3	0.8859566122993233	00:01:04.64169	false
60	Constant Removal, Standardization	LASSO Feature Selection	penalty = 1.0	Support Vector Machines (SVM) of type C-SVC	kernel = 'Radial Basis Function Kernel', cost = 0.1, gamma = 1.0	0.9203035171705249	00:03:40.220412	false
61	Constant Removal, Standardization	Test-Budgeted Statistically Equivalent Signature (SES) algorithm	maxK = 3, alpha = 0.01, budget = 3 * nvars	Classification Random Forests with Deviance splitting criterion	ntrees = 500, minimum leaf size = 2	0.8897023692420112	00:00:41.41279	false
62	Constant Removal, Standardization	LASSO Feature Selection	penalty = 0.5	Classification Decision Tree with Deviance splitting criterion	minimum leaf size = 3, alpha = 0.05	0.870447008234732	00:03:40.220183	false
63	Constant Removal, Standardization	Test-Budgeted Statistically Equivalent Signature (SES) algorithm	maxK = 2, alpha = 0.05, budget = 3 * nvars	Classification Random Forests with Deviance splitting criterion	ntrees = 100, minimum leaf size = 3	0.8776988498855506	00:00:33.33198	false
64	Constant Removal, Standardization	Test-Budgeted Statistically Equivalent Signature (SES) algorithm	maxK = 2, alpha = 0.05, budget = 3 * nvars	Support Vector Machines (SVM) of type C-SVC	kernel = 'Polynomial Kernel', cost = 10.0, gamma = 1.0, degree = 2	0.7271331609566903	00:00:33.33190	false
65	Constant Removal, Standardization	Test-Budgeted Statistically Equivalent Signature (SES) algorithm	maxK = 2, alpha = 0.05, budget = 3 * nvars	Classification Random Forests with Deviance splitting criterion	ntrees = 100, minimum leaf size = 4	0.8745347079746056	00:00:33.33198	false
66	Constant Removal, Standardization	LASSO Feature Selection	penalty = 0.5	Classification Random Forests with Deviance splitting criterion	ntrees = 500, minimum leaf size = 4	0.9328937260522939	00:03:40.220256	false

Configuration	Preprocessing	Name	Hyperparams	Name	Hyperparams	Performance (unadjusted)	Time (miliseconds)	Dropped
67	Constant Removal, Standardization	Test-Budgeted Statistically Equivalent Signature (SES) algorithm	maxK = 2, alpha = 0.05, budget = 3 * nvars	Support Vector Machines (SVM) of type C-SVC	kernel = 'Polynomial Kernel', cost = 0.01, gamma = 0.1, degree = 2	0.9032828850603786	00:00:33.33190	false
68	Constant Removal, Standardization	LASSO Feature Selection	penalty = 0.5	Support Vector Machines (SVM) of type C-SVC	kernel = 'Polynomial Kernel', cost = 1.0, gamma = 1.0, degree = 3	0.5	00:03:40.220180	false
69	Constant Removal, Standardization	Test-Budgeted Statistically Equivalent Signature (SES) algorithm	maxK = 2, alpha = 0.01, budget = 3 * nvars	Support Vector Machines (SVM) of type C-SVC	kernel = 'Polynomial Kernel', cost = 0.01, gamma = 1.0, degree = 3	0.874481620964997	00:00:40.40983	false
70	Constant Removal, Standardization	LASSO Feature Selection	penalty = 1.0	Classification Random Forests with Deviance splitting criterion	ntrees = 500, minimum leaf size = 4	0.9328937260522939	00:03:40.220476	false
71	Constant Removal, Standardization	Test-Budgeted Statistically Equivalent Signature (SES) algorithm	maxK = 2, alpha = 0.01, budget = 3 * nvars	Support Vector Machines (SVM) of type C-SVC	kernel = 'Radial Basis Function Kernel', cost = 10.0, gamma = 0.01	0.7631484031739786	00:00:40.40984	false
72	Constant Removal, Standardization	Test-Budgeted Statistically Equivalent Signature (SES) algorithm	maxK = 3, alpha = 0.01, budget = 3 * nvars	Support Vector Machines (SVM) of type C-SVC	kernel = 'Polynomial Kernel', cost = 1.0, gamma = 0.01, degree = 2	0.8569511974243432	00:00:41.41205	false
73	Constant Removal, Standardization	Test-Budgeted Statistically Equivalent Signature (SES) algorithm	maxK = 2, alpha = 0.05, budget = 3 * nvars	Support Vector Machines (SVM) of type C-SVC	kernel = 'Polynomial Kernel', cost = 0.01, gamma = 10.0, degree = 3	0.7988720571840777	00:00:33.33191	false
74	Constant Removal, Standardization	LASSO Feature Selection	penalty = 0.5	Support Vector Machines (SVM) of type C-SVC	kernel = 'Polynomial Kernel', cost = 10.0, gamma = 0.01, degree = 2	0.9159140802363309	00:03:40.220180	false
75	Constant Removal, Standardization	Test-Budgeted Statistically Equivalent Signature (SES) algorithm	maxK = 2, alpha = 0.05, budget = 3 * nvars	Support Vector Machines (SVM) of type C-SVC	kernel = 'Radial Basis Function Kernel', cost = 10.0, gamma = 10.0	0.8012933713061591	00:00:33.33194	false
76	Constant Removal, Standardization	LASSO Feature Selection	penalty = 0.5	Support Vector Machines (SVM) of type C-SVC	kernel = 'Radial Basis Function Kernel', cost = 10.0, gamma = 0.1	0.9212192680862755	00:03:40.220181	false
77	Constant Removal, Standardization	Test-Budgeted Statistically Equivalent Signature (SES) algorithm	maxK = 3, alpha = 0.05, budget = 3 * nvars	Support Vector Machines (SVM) of type C-SVC	kernel = 'Radial Basis Function Kernel', cost = 10.0, gamma = 0.01	0.7477242848086837	00:01:04.64126	false

Configuration	Preprocessing	Name	Hyperparams	Name	Hyperparams	Performance (unadjusted)	Time (miliseconds)	Dropped
78	Constant Removal, Standardization	LASSO Feature Selection	penalty = 0.5	Support Vector Machines (SVM) of type C-SVC	kernel = 'Polynomial Kernel', cost = 10.0, gamma = 1.0, degree = 3	0.5	00:03:40.220180	false
79	Constant Removal, Standardization	LASSO Feature Selection	penalty = 1.0	Support Vector Machines (SVM) of type C-SVC	kernel = 'Polynomial Kernel', cost = 0.01, gamma = 0.01, degree = 3	0.9256215864272129	00:03:40.220410	false
80	Constant Removal, Standardization	LASSO Feature Selection	penalty = 1.5	Support Vector Machines (SVM) of type C-SVC	kernel = 'Radial Basis Function Kernel', cost = 0.01, gamma = 10.0	0.8724475297365324	00:03:39.219721	false
81	Constant Removal, Standardization	Test-Budgeted Statistically Equivalent Signature (SES) algorithm	maxK = 2, alpha = 0.05, budget = 3 * nvars	Classification Random Forests with Deviance splitting criterion	ntrees = 100, minimum leaf size = 2	0.8880449615743733	00:00:33.33206	false
82	Constant Removal, Standardization	Test-Budgeted Statistically Equivalent Signature (SES) algorithm	maxK = 3, alpha = 0.05, budget = 3 * nvars	Support Vector Machines (SVM) of type C-SVC	kernel = 'Radial Basis Function Kernel', cost = 10.0, gamma = 0.1	0.862127571206855	00:01:04.64127	false
83	Constant Removal, Standardization	LASSO Feature Selection	penalty = 1.0	Classification Random Forests with Deviance splitting criterion	ntrees = 100, minimum leaf size = 4	0.9307144262387228	00:03:40.220416	false
84	Constant Removal, Standardization	Test-Budgeted Statistically Equivalent Signature (SES) algorithm	maxK = 3, alpha = 0.05, budget = 3 * nvars	Support Vector Machines (SVM) of type C-SVC	kernel = 'Polynomial Kernel', cost = 0.01, gamma = 0.1, degree = 2	0.8978043837378876	00:01:04.64125	false
85	Constant Removal, Standardization	Test-Budgeted Statistically Equivalent Signature (SES) algorithm	maxK = 3, alpha = 0.05, budget = 3 * nvars	Classification Random Forests with Deviance splitting criterion	ntrees = 100, minimum leaf size = 2	0.8895575510025636	00:01:04.64133	false
86	Constant Removal, Standardization	Test-Budgeted Statistically Equivalent Signature (SES) algorithm	maxK = 2, alpha = 0.01, budget = 3 * nvars	Support Vector Machines (SVM) of type C-SVC	kernel = 'Radial Basis Function Kernel', cost = 10.0, gamma = 10.0	0.8012933713061591	00:00:40.40986	false
87	Constant Removal, Standardization	Test-Budgeted Statistically Equivalent Signature (SES) algorithm	maxK = 2, alpha = 0.05, budget = 3 * nvars	Support Vector Machines (SVM) of type C-SVC	kernel = 'Polynomial Kernel', cost = 0.01, gamma = 0.01, degree = 3	0.9070825097039933	00:00:33.33190	false
88	Constant Removal, Standardization	Test-Budgeted Statistically Equivalent Signature (SES) algorithm	maxK = 2, alpha = 0.01, budget = 3 * nvars	Classification Decision Tree with Deviance splitting criterion	minimum leaf size = 2, alpha = 0.05	0.7743702553485162	00:00:40.40983	false

Configuration	Preprocessing	Name	Hyperparams	Name	Hyperparams	Performance (unadjusted)	Time (miliseconds)	Dropped
89	Constant Removal, Standardization	Test-Budgeted Statistically Equivalent Signature (SES) algorithm	maxK = 2, alpha = 0.01, budget = 3 * nvars	Support Vector Machines (SVM) of type C-SVC	kernel = 'Polynomial Kernel', cost = 0.1, gamma = 0.1, degree = 3	0.8117245783486193	00:00:40.40981	false
90	Constant Removal, Standardization	Test-Budgeted Statistically Equivalent Signature (SES) algorithm	maxK = 3, alpha = 0.01, budget = 3 * nvars	Support Vector Machines (SVM) of type C-SVC	kernel = 'Radial Basis Function Kernel', cost = 0.01, gamma = 1.0	0.8762280274428612	00:00:41.41206	false
91	Constant Removal, Standardization	Test-Budgeted Statistically Equivalent Signature (SES) algorithm	maxK = 3, alpha = 0.01, budget = 3 * nvars	Support Vector Machines (SVM) of type C-SVC	kernel = 'Radial Basis Function Kernel', cost = 0.1, gamma = 0.01	0.8493289177432398	00:00:41.41206	false
92	Constant Removal, Standardization	LASSO Feature Selection	penalty = 1.0	Classification Random Forests with Deviance splitting criterion	ntrees = 100, minimum leaf size = 3	0.93945504623254	00:03:40.220422	false
93	Constant Removal, Standardization	LASSO Feature Selection	penalty = 1.0	Support Vector Machines (SVM) of type C-SVC	kernel = 'Radial Basis Function Kernel', cost = 1.0, gamma = 1.0	0.9163231624868453	00:03:40.220412	false
94	Constant Removal, Standardization	LASSO Feature Selection	penalty = 1.5	Support Vector Machines (SVM) of type C-SVC	kernel = 'Polynomial Kernel', cost = 0.1, gamma = 0.1, degree = 2	0.6717985410440653	00:03:39.219719	false
95	Constant Removal, Standardization	LASSO Feature Selection	penalty = 1.0	Support Vector Machines (SVM) of type C-SVC	kernel = 'Polynomial Kernel', cost = 0.1, gamma = 10.0, degree = 3	0.7986382401344039	00:03:40.220410	false
96	Constant Removal, Standardization	LASSO Feature Selection	penalty = 0.5	Classification Random Forests with Deviance splitting criterion	ntrees = 500, minimum leaf size = 3	0.9310438779748242	00:03:40.220252	false
97	Constant Removal, Standardization	LASSO Feature Selection	penalty = 0.5	Classification Random Forests with Deviance splitting criterion	ntrees = 100, minimum leaf size = 4	0.9359239794022403	00:03:40.220195	false
98	Constant Removal, Standardization	Test-Budgeted Statistically Equivalent Signature (SES) algorithm	maxK = 2, alpha = 0.05, budget = 3 * nvars	Support Vector Machines (SVM) of type C-SVC	kernel = 'Polynomial Kernel', cost = 0.1, gamma = 10.0, degree = 3	0.778636538227331	00:00:33.33191	false
99	Constant Removal, Standardization	Test-Budgeted Statistically Equivalent Signature (SES) algorithm	maxK = 2, alpha = 0.01, budget = 3 * nvars	Classification Random Forests with Deviance splitting criterion	ntrees = 100, minimum leaf size = 3	0.8794159804389983	00:00:40.40994	false
100	Constant Removal, Standardization	LASSO Feature Selection	penalty = 1.0	Support Vector Machines	kernel = 'Polynomial Kernel', cost =	0.5	00:03:40.220410	false

Configuration	Preprocessing	Name	Hyperparams	Name	Hyperparams	Performance (unadjusted)	Time (miliseconds)	Dropped
				(SVM) of type C-SVC	1.0, gamma = 0.1, degree = 3			
101	Constant Removal, Standardization	Test-Budgeted Statistically Equivalent Signature (SES) algorithm	maxK = 2, alpha = 0.01, budget = 3 * nvars	Support Vector Machines (SVM) of type C-SVC	kernel = 'Polynomial Kernel', cost = 1.0, gamma = 10.0, degree = 3	0.778636538227331	00:00:40.40983	false
102	Constant Removal, Standardization	Test-Budgeted Statistically Equivalent Signature (SES) algorithm	maxK = 2, alpha = 0.05, budget = 3 * nvars	Classification Random Forests with Deviance splitting criterion	ntrees = 500, minimum leaf size = 3	0.8864234657073533	00:00:33.33265	false
103	Constant Removal, Standardization	Test-Budgeted Statistically Equivalent Signature (SES) algorithm	maxK = 2, alpha = 0.05, budget = 3 * nvars	Support Vector Machines (SVM) of type C-SVC	kernel = 'Polynomial Kernel', cost = 1.0, gamma = 0.01, degree = 2	0.8702748657991624	00:00:33.33190	false
104	Constant Removal, Standardization	Test-Budgeted Statistically Equivalent Signature (SES) algorithm	maxK = 2, alpha = 0.01, budget = 3 * nvars	Classification Decision Tree with Deviance splitting criterion	minimum leaf size = 4, alpha = 0.01	0.8117241880029605	00:00:40.40983	false
105	Constant Removal, Standardization	LASSO Feature Selection	penalty = 1.5	Ridge Logistic Regression	lambda = 10.0	0.92421868412917	00:03:39.219719	false
106	Constant Removal, Standardization	Test-Budgeted Statistically Equivalent Signature (SES) algorithm	maxK = 3, alpha = 0.05, budget = 3 * nvars	Support Vector Machines (SVM) of type C-SVC	kernel = 'Polynomial Kernel', cost = 10.0, gamma = 0.1, degree = 3	0.7800109452922752	00:01:04.64125	false
107	Constant Removal, Standardization	Test-Budgeted Statistically Equivalent Signature (SES) algorithm	maxK = 3, alpha = 0.01, budget = 3 * nvars	Classification Random Forests with Deviance splitting criterion	ntrees = 100, minimum leaf size = 2	0.8914323812022021	00:00:41.41218	false
108	Constant Removal, Standardization	Test-Budgeted Statistically Equivalent Signature (SES) algorithm	maxK = 3, alpha = 0.05, budget = 3 * nvars	Support Vector Machines (SVM) of type C-SVC	kernel = 'Polynomial Kernel', cost = 1.0, gamma = 1.0, degree = 2	0.7585610609907285	00:01:04.64124	false
109	Constant Removal, Standardization	LASSO Feature Selection	penalty = 1.0	Support Vector Machines (SVM) of type C-SVC	kernel = 'Polynomial Kernel', cost = 0.01, gamma = 0.01, degree = 2	0.9253381954788606	00:03:40.220410	false
110	Constant Removal, Standardization	LASSO Feature Selection	penalty = 1.0	Support Vector Machines (SVM) of type C-SVC	kernel = 'Radial Basis Function Kernel', cost = 0.01, gamma = 0.1	0.9278617801635706	00:03:40.220412	false
111	Constant Removal, Standardization	Test-Budgeted Statistically Equivalent Signature (SES) algorithm	maxK = 2, alpha = 0.05, budget = 3 * nvars	Classification Decision Tree with Deviance splitting criterion	minimum leaf size = 2, alpha = 0.01	0.7802923845123334	00:00:33.33194	false

Configuration	Preprocessing	Name	Hyperparams	Name	Hyperparams	Performance (unadjusted)	Time (miliseconds)	Dropped
112	Constant Removal, Standardization	Test-Budgeted Statistically Equivalent Signature (SES) algorithm	maxK = 2, alpha = 0.01, budget = 3 * nvars	Support Vector Machines (SVM) of type C-SVC	kernel = 'Radial Basis Function Kernel', cost = 0.1, gamma = 0.1	0.8659787214774427	00:00:40.40982	false
113	Constant Removal, Standardization	LASSO Feature Selection	penalty = 1.5	Support Vector Machines (SVM) of type C-SVC	kernel = 'Radial Basis Function Kernel', cost = 10.0, gamma = 1.0	0.9156127333876695	00:03:39.219721	false
114	Constant Removal, Standardization	LASSO Feature Selection	penalty = 1.5	Support Vector Machines (SVM) of type C-SVC	kernel = 'Polynomial Kernel', cost = 1.0, gamma = 10.0, degree = 2	0.4761576871551296	00:03:39.219719	false
115	Constant Removal, Standardization	Test-Budgeted Statistically Equivalent Signature (SES) algorithm	maxK = 2, alpha = 0.05, budget = 3 * nvars	Classification Random Forests with Deviance splitting criterion	ntrees = 500, minimum leaf size = 3	0.8792075358571522	00:00:33.33237	false
116	Constant Removal, Standardization	Test-Budgeted Statistically Equivalent Signature (SES) algorithm	maxK = 2, alpha = 0.01, budget = 3 * nvars	Support Vector Machines (SVM) of type C-SVC	kernel = 'Polynomial Kernel', cost = 0.01, gamma = 0.1, degree = 2	0.9032828850603786	00:00:40.40983	false
117	Constant Removal, Standardization	Test-Budgeted Statistically Equivalent Signature (SES) algorithm	maxK = 3, alpha = 0.01, budget = 3 * nvars	Support Vector Machines (SVM) of type C-SVC	kernel = 'Polynomial Kernel', cost = 0.01, gamma = 10.0, degree = 3	0.8264808152915569	00:00:41.41206	false
118	Constant Removal, Standardization	Test-Budgeted Statistically Equivalent Signature (SES) algorithm	maxK = 3, alpha = 0.01, budget = 3 * nvars	Support Vector Machines (SVM) of type C-SVC	kernel = 'Polynomial Kernel', cost = 10.0, gamma = 0.1, degree = 3	0.7800109452922752	00:00:41.41205	false
119	Constant Removal, Standardization	Test-Budgeted Statistically Equivalent Signature (SES) algorithm	maxK = 3, alpha = 0.05, budget = 3 * nvars	Classification Random Forests with Deviance splitting criterion	ntrees = 100, minimum leaf size = 3	0.8824712159111135	00:01:04.64132	false
120	Constant Removal, Standardization	Test-Budgeted Statistically Equivalent Signature (SES) algorithm	maxK = 3, alpha = 0.01, budget = 3 * nvars	Support Vector Machines (SVM) of type C-SVC	kernel = 'Polynomial Kernel', cost = 1.0, gamma = 10.0, degree = 3	0.808102756152628	00:00:41.41206	false
121	Constant Removal, Standardization	Test-Budgeted Statistically Equivalent Signature (SES) algorithm	maxK = 2, alpha = 0.05, budget = 3 * nvars	Support Vector Machines (SVM) of type C-SVC	kernel = 'Radial Basis Function Kernel', cost = 0.1, gamma = 0.1	0.8659787214774427	00:00:33.33192	false
122	Constant Removal, Standardization	Test-Budgeted Statistically Equivalent Signature (SES)	maxK = 3, alpha = 0.01, budget = 3 * nvars	Support Vector Machines (SVM) of type C-SVC	kernel = 'Polynomial Kernel', cost = 0.01, gamma = 1.0, degree = 3	0.861526438892168	00:00:41.41205	false

Configuration	Preprocessing	Name	Hyperparams	Name	Hyperparams	Performance (unadjusted)	Time (miliseconds)	Dropped
		algorithm						
123	Constant Removal, Standardization	Test-Budgeted Statistically Equivalent Signature (SES) algorithm	maxK = 2, alpha = 0.01, budget = 3 * nvars	Classification Decision Tree with Deviance splitting criterion	minimum leaf size = 2, alpha = 0.01	0.7802923845123334	00:00:40.40984	false
124	Constant Removal, Standardization	Test-Budgeted Statistically Equivalent Signature (SES) algorithm	maxK = 2, alpha = 0.05, budget = 3 * nvars	Support Vector Machines (SVM) of type C-SVC	kernel = 'Polynomial Kernel', cost = 1.0, gamma = 1.0, degree = 2	0.7271331609566903	00:00:33.33190	false
125	Constant Removal, Standardization	Test-Budgeted Statistically Equivalent Signature (SES) algorithm	maxK = 3, alpha = 0.05, budget = 3 * nvars	Classification Random Forests with Deviance splitting criterion	ntrees = 100, minimum leaf size = 2	0.8914323812022021	00:01:04.64140	false
126	Constant Removal, Standardization	Test-Budgeted Statistically Equivalent Signature (SES) algorithm	maxK = 3, alpha = 0.01, budget = 3 * nvars	Classification Random Forests with Deviance splitting criterion	ntrees = 100, minimum leaf size = 3	0.8824712159111135	00:00:41.41212	false
127	Constant Removal, Standardization	Test-Budgeted Statistically Equivalent Signature (SES) algorithm	maxK = 2, alpha = 0.05, budget = 3 * nvars	Classification Decision Tree with Deviance splitting criterion	minimum leaf size = 4, alpha = 0.01	0.8117241880029605	00:00:33.33193	false
128	Constant Removal, Standardization	Test-Budgeted Statistically Equivalent Signature (SES) algorithm	maxK = 2, alpha = 0.01, budget = 3 * nvars	Support Vector Machines (SVM) of type C-SVC	kernel = 'Polynomial Kernel', cost = 1.0, gamma = 0.01, degree = 2	0.8702748657991624	00:00:40.40983	false
129	Constant Removal, Standardization	Test-Budgeted Statistically Equivalent Signature (SES) algorithm	maxK = 3, alpha = 0.05, budget = 3 * nvars	Support Vector Machines (SVM) of type C-SVC	kernel = 'Polynomial Kernel', cost = 0.01, gamma = 10.0, degree = 3	0.8264808152915569	00:01:04.64125	false
130	Constant Removal, Standardization	LASSO Feature Selection	penalty = 1.0	Ridge Logistic Regression	lambda = 1.0	0.921970683479635	00:03:40.220410	false
131	Constant Removal, Standardization	LASSO Feature Selection	penalty = 1.5	Support Vector Machines (SVM) of type C-SVC	kernel = 'Polynomial Kernel', cost = 0.01, gamma = 10.0, degree = 2	0.6832458178366106	00:03:39.219719	false
132	Constant Removal, Standardization	Test-Budgeted Statistically Equivalent Signature (SES) algorithm	maxK = 3, alpha = 0.05, budget = 3 * nvars	Support Vector Machines (SVM) of type C-SVC	kernel = 'Radial Basis Function Kernel', cost = 10.0, gamma = 10.0	0.8146517804446193	00:01:04.64128	false
133	Constant Removal, Standardization	Test-Budgeted Statistically Equivalent Signature (SES) algorithm	maxK = 3, alpha = 0.01, budget = 3 * nvars	Classification Random Forests with Deviance splitting criterion	ntrees = 100, minimum leaf size = 2	0.8895575510025636	00:00:41.41212	false

Configuration	Preprocessing	Name	Hyperparams	Name	Hyperparams	Performance (unadjusted)	Time (miliseconds)	Dropped
134	Constant Removal, Standardization	Test-Budgeted Statistically Equivalent Signature (SES) algorithm	maxK = 2, alpha = 0.05, budget = 3 * nvars	Support Vector Machines (SVM) of type C-SVC	kernel = 'Radial Basis Function Kernel', cost = 10.0, gamma = 0.01	0.7631484031739786	00:00:33.33192	false
135	Constant Removal, Standardization	Test-Budgeted Statistically Equivalent Signature (SES) algorithm	maxK = 3, alpha = 0.05, budget = 3 * nvars	Classification Random Forests with Deviance splitting criterion	ntrees = 100, minimum leaf size = 2	0.8914323812022021	00:01:04.64139	false
136	Constant Removal, Standardization	Test-Budgeted Statistically Equivalent Signature (SES) algorithm	maxK = 2, alpha = 0.05, budget = 3 * nvars	Support Vector Machines (SVM) of type C-SVC	kernel = 'Radial Basis Function Kernel', cost = 10.0, gamma = 0.1	0.8611532684422709	00:00:33.33192	false
137	Constant Removal, Standardization	LASSO Feature Selection	penalty = 1.5	Classification Random Forests with Deviance splitting criterion	ntrees = 100, minimum leaf size = 3	0.9408759044308916	00:03:39.219731	false
138	Constant Removal, Standardization	Test-Budgeted Statistically Equivalent Signature (SES) algorithm	maxK = 2, alpha = 0.01, budget = 3 * nvars	Support Vector Machines (SVM) of type C-SVC	kernel = 'Polynomial Kernel', cost = 0.01, gamma = 10.0, degree = 2	0.6585267886418782	00:00:40.40983	false
139	Constant Removal, Standardization	LASSO Feature Selection	penalty = 0.5	Support Vector Machines (SVM) of type C-SVC	kernel = 'Radial Basis Function Kernel', cost = 0.01, gamma = 0.01	0.922478523181848	00:03:40.220182	false
140	Constant Removal, Standardization	LASSO Feature Selection	penalty = 1.0	Classification Random Forests with Deviance splitting criterion	ntrees = 500, minimum leaf size = 3	0.9310438779748242	00:03:40.220479	false
141	Constant Removal, Standardization	LASSO Feature Selection	penalty = 1.0	Support Vector Machines (SVM) of type C-SVC	kernel = 'Linear Kernel', cost = 0.01	0.9253202395785518	00:03:40.220410	false
142	Constant Removal, Standardization	Test-Budgeted Statistically Equivalent Signature (SES) algorithm	maxK = 2, alpha = 0.01, budget = 3 * nvars	Support Vector Machines (SVM) of type C-SVC	kernel = 'Radial Basis Function Kernel', cost = 0.01, gamma = 1.0	0.874969162692948	00:00:40.40983	false
143	Constant Removal, Standardization	Test-Budgeted Statistically Equivalent Signature (SES) algorithm	maxK = 3, alpha = 0.05, budget = 3 * nvars	Classification Random Forests with Deviance splitting criterion	ntrees = 500, minimum leaf size = 3	0.8890407333501961	00:01:04.64202	false
144	Constant Removal, Standardization	Test-Budgeted Statistically Equivalent Signature (SES) algorithm	maxK = 3, alpha = 0.05, budget = 3 * nvars	Support Vector Machines (SVM) of type C-SVC	kernel = 'Radial Basis Function Kernel', cost = 0.01, gamma = 1.0	0.8762280274428612	00:01:04.64126	false

Configuration	Preprocessing	Name	Hyperparams	Name	Hyperparams	Performance (unadjusted)	Time (miliseconds)	Dropped
145	Constant Removal, Standardization	Test-Budgeted Statistically Equivalent Signature (SES) algorithm	maxK = 2, alpha = 0.05, budget = 3 * nvars	Support Vector Machines (SVM) of type C-SVC	kernel = 'Radial Basis Function Kernel', cost = 1.0, gamma = 10.0	0.8009561126568799	00:00:33.33193	false
146	Constant Removal, Standardization	Test-Budgeted Statistically Equivalent Signature (SES) algorithm	maxK = 2, alpha = 0.01, budget = 3 * nvars	Classification Random Forests with Deviance splitting criterion	ntrees = 500, minimum leaf size = 2	0.8868376224514332	00:00:41.41073	false
147	Constant Removal, Standardization	Test-Budgeted Statistically Equivalent Signature (SES) algorithm	maxK = 2, alpha = 0.01, budget = 3 * nvars	Classification Random Forests with Deviance splitting criterion	ntrees = 500, minimum leaf size = 3	0.8864234657073533	00:00:41.41068	false
148	Constant Removal, Standardization	Test-Budgeted Statistically Equivalent Signature (SES) algorithm	maxK = 3, alpha = 0.01, budget = 3 * nvars	Classification Random Forests with Deviance splitting criterion	ntrees = 100, minimum leaf size = 3	0.889528665423806	00:00:41.41218	false
149	Constant Removal, Standardization	Test-Budgeted Statistically Equivalent Signature (SES) algorithm	maxK = 3, alpha = 0.01, budget = 3 * nvars	Classification Decision Tree with Deviance splitting criterion	minimum leaf size = 2, alpha = 0.05	0.7892211511137344	00:00:41.41208	false
150	Constant Removal, Standardization	LASSO Feature Selection	penalty = 0.5	Support Vector Machines (SVM) of type C-SVC	kernel = 'Linear Kernel', cost = 0.1	0.9253202395785518	00:03:40.220180	false
151	Constant Removal, Standardization	LASSO Feature Selection	penalty = 0.5	Classification Random Forests with Deviance splitting criterion	ntrees = 500, minimum leaf size = 4	0.9320045186413474	00:03:40.220222	false
152	Constant Removal, Standardization	Test-Budgeted Statistically Equivalent Signature (SES) algorithm	maxK = 3, alpha = 0.01, budget = 3 * nvars	Support Vector Machines (SVM) of type C-SVC	kernel = 'Radial Basis Function Kernel', cost = 10.0, gamma = 0.1	0.862127571206855	00:00:41.41206	false
153	Constant Removal, Standardization	LASSO Feature Selection	penalty = 0.5	Support Vector Machines (SVM) of type C-SVC	kernel = 'Polynomial Kernel', cost = 10.0, gamma = 1.0, degree = 2	0.5	00:03:40.220180	false
154	Constant Removal, Standardization	LASSO Feature Selection	penalty = 1.0	Ridge Logistic Regression	lambda = 0.1	0.9216513807306647	00:03:40.220410	false
155	Constant Removal, Standardization	LASSO Feature Selection	penalty = 1.5	Support Vector Machines (SVM) of type C-SVC	kernel = 'Polynomial Kernel', cost = 10.0, gamma = 0.01, degree = 2	0.9176901529842706	00:03:39.219719	false
156	Constant Removal, Standardization	LASSO Feature Selection	penalty = 1.5	Support Vector Machines (SVM) of type C-SVC	kernel = 'Linear Kernel', cost = 10.0	0.9249650250289635	00:03:39.219723	false

Configuration	Preprocessing	Name	Hyperparams	Name	Hyperparams	Performance (unadjusted)	Time (miliseconds)	Dropped
157	Constant Removal, Standardization	LASSO Feature Selection	penalty = 0.5	Classification Random Forests with Deviance splitting criterion	ntrees = 500, minimum leaf size = 2	0.9332309847015732	00:03:40.220262	false
158	Constant Removal, Standardization	Test-Budgeted Statistically Equivalent Signature (SES) algorithm	maxK = 3, alpha = 0.05, budget = 3 * nvars	Support Vector Machines (SVM) of type C-SVC	kernel = 'Polynomial Kernel', cost = 1.0, gamma = 1.0, degree = 3	0.7662262786943094	00:01:04.64125	false
159	Constant Removal, Standardization	LASSO Feature Selection	penalty = 1.5	Classification Random Forests with Deviance splitting criterion	ntrees = 500, minimum leaf size = 2	0.934296628350337	00:03:39.219792	false
160	Constant Removal, Standardization	LASSO Feature Selection	penalty = 0.5	Classification Random Forests with Deviance splitting criterion	ntrees = 500, minimum leaf size = 2	0.9332309847015732	00:03:40.220259	false
161	Constant Removal, Standardization	Test-Budgeted Statistically Equivalent Signature (SES) algorithm	maxK = 3, alpha = 0.01, budget = 3 * nvars	Classification Random Forests with Deviance splitting criterion	ntrees = 500, minimum leaf size = 2	0.8897023692420112	00:00:41.41282	false
162	Constant Removal, Standardization	LASSO Feature Selection	penalty = 1.5	Classification Decision Tree with Deviance splitting criterion	minimum leaf size = 4, alpha = 0.01	0.8944519000465293	00:03:39.219722	false
163	Constant Removal, Standardization	Test-Budgeted Statistically Equivalent Signature (SES) algorithm	maxK = 2, alpha = 0.01, budget = 3 * nvars	Support Vector Machines (SVM) of type C-SVC	kernel = 'Radial Basis Function Kernel', cost = 0.01, gamma = 0.1	0.8904654950051369	00:00:40.40982	false
164	Constant Removal, Standardization	Test-Budgeted Statistically Equivalent Signature (SES) algorithm	maxK = 2, alpha = 0.01, budget = 3 * nvars	Support Vector Machines (SVM) of type C-SVC	kernel = 'Polynomial Kernel', cost = 1.0, gamma = 0.1, degree = 3	0.7463830571247452	00:00:40.40981	false
165	Constant Removal, Standardization	Test-Budgeted Statistically Equivalent Signature (SES) algorithm	maxK = 2, alpha = 0.05, budget = 3 * nvars	Support Vector Machines (SVM) of type C-SVC	kernel = 'Polynomial Kernel', cost = 0.1, gamma = 0.1, degree = 3	0.8117245783486193	00:00:33.33190	false
166	Constant Removal, Standardization	Test-Budgeted Statistically Equivalent Signature (SES) algorithm	maxK = 2, alpha = 0.01, budget = 3 * nvars	Ridge Logistic Regression	lambda = 10.0	0.884920634920635	00:00:40.40979	false
167	Constant Removal, Standardization	LASSO Feature Selection	penalty = 1.5	Classification Decision Tree with Deviance splitting criterion	minimum leaf size = 2, alpha = 0.05	0.8440024560548857	00:03:39.219722	false
168	Constant Removal, Standardization	LASSO Feature Selection	penalty = 1.0	Support Vector Machines	kernel = 'Polynomial Kernel', cost =	0.9176979598974483	00:03:40.220410	false

Configuration	Preprocessing	Name	Hyperparams	Name	Hyperparams	Performance (unadjusted)	Time (miliseconds)	Dropped
				(SVM) of type C-SVC	10.0, gamma = 0.01, degree = 3			
169	Constant Removal, Standardization	Test-Budgeted Statistically Equivalent Signature (SES) algorithm	maxK = 2, alpha = 0.05, budget = 3 * nvars	Support Vector Machines (SVM) of type C-SVC	kernel = 'Polynomial Kernel', cost = 0.1, gamma = 0.01, degree = 2	0.8721469635791887	00:00:33.33190	false
170	Constant Removal, Standardization	LASSO Feature Selection	penalty = 0.5	Support Vector Machines (SVM) of type C-SVC	kernel = 'Radial Basis Function Kernel', cost = 10.0, gamma = 0.01	0.9146638030909131	00:03:40.220182	false
171	Constant Removal, Standardization	Test-Budgeted Statistically Equivalent Signature (SES) algorithm	maxK = 2, alpha = 0.05, budget = 3 * nvars	Support Vector Machines (SVM) of type C-SVC	kernel = 'Polynomial Kernel', cost = 0.01, gamma = 10.0, degree = 2	0.6585267886418782	00:00:33.33190	false
172	Constant Removal, Standardization	Test-Budgeted Statistically Equivalent Signature (SES) algorithm	maxK = 3, alpha = 0.05, budget = 3 * nvars	Classification Decision Tree with Deviance splitting criterion	minimum leaf size = 3, alpha = 0.01	0.8101001548891574	00:01:04.64128	false
173	Constant Removal, Standardization	LASSO Feature Selection	penalty = 0.5	Support Vector Machines (SVM) of type C-SVC	kernel = 'Polynomial Kernel', cost = 1.0, gamma = 0.1, degree = 2	0.7087129444866018	00:03:40.220180	false
174	Constant Removal, Standardization	LASSO Feature Selection	penalty = 1.5	Support Vector Machines (SVM) of type C-SVC	kernel = 'Polynomial Kernel', cost = 10.0, gamma = 10.0, degree = 2	0.4761576871551296	00:03:39.219719	false
175	Constant Removal, Standardization	Test-Budgeted Statistically Equivalent Signature (SES) algorithm	maxK = 3, alpha = 0.05, budget = 3 * nvars	Support Vector Machines (SVM) of type C-SVC	kernel = 'Radial Basis Function Kernel', cost = 0.01, gamma = 0.01	0.8800920591201922	00:01:04.64127	false
176	Constant Removal, Standardization	Test-Budgeted Statistically Equivalent Signature (SES) algorithm	maxK = 3, alpha = 0.01, budget = 3 * nvars	Support Vector Machines (SVM) of type C-SVC	kernel = 'Linear Kernel', cost = 0.01	0.8955153967941691	00:00:41.41205	false
177	Constant Removal, Standardization	Test-Budgeted Statistically Equivalent Signature (SES) algorithm	maxK = 3, alpha = 0.01, budget = 3 * nvars	Classification Random Forests with Deviance splitting criterion	ntrees = 500, minimum leaf size = 4	0.8905763531722611	00:00:41.41275	false
178	Constant Removal, Standardization	Test-Budgeted Statistically Equivalent Signature (SES) algorithm	maxK = 3, alpha = 0.01, budget = 3 * nvars	Classification Random Forests with Deviance splitting criterion	ntrees = 500, minimum leaf size = 4	0.8905763531722611	00:00:41.41275	false

Configuration	Preprocessing	Name	Hyperparams	Name	Hyperparams	Performance (unadjusted)	Time (miliseconds)	Dropped
179	Constant Removal, Standardization	LASSO Feature Selection	penalty = 1.5	Classification Decision Tree with Deviance splitting criterion	minimum leaf size = 3, alpha = 0.01	0.8980112669370981	00:03:39.219722	false
180	Constant Removal, Standardization	LASSO Feature Selection	penalty = 0.5	Support Vector Machines (SVM) of type C-SVC	kernel = 'Polynomial Kernel', cost = 10.0, gamma = 10.0, degree = 3	0.7986382401344039	00:03:40.220180	false
181	Constant Removal, Standardization	Test-Budgeted Statistically Equivalent Signature (SES) algorithm	maxK = 3, alpha = 0.05, budget = 3 * nvars	Support Vector Machines (SVM) of type C-SVC	kernel = 'Polynomial Kernel', cost = 10.0, gamma = 10.0, degree = 2	0.6303984804624191	00:01:04.64125	false
182	Constant Removal, Standardization	LASSO Feature Selection	penalty = 0.5	Support Vector Machines (SVM) of type C-SVC	kernel = 'Polynomial Kernel', cost = 0.1, gamma = 10.0, degree = 2	0.5637766254773928	00:03:40.220179	false
183	Constant Removal, Standardization	LASSO Feature Selection	penalty = 1.0	Support Vector Machines (SVM) of type C-SVC	kernel = 'Polynomial Kernel', cost = 10.0, gamma = 0.1, degree = 2	0.7087129444866018	00:03:40.220410	false
184	Constant Removal, Standardization	LASSO Feature Selection	penalty = 0.5	Classification Random Forests with Deviance splitting criterion	ntrees = 500, minimum leaf size = 3	0.9314857492606854	00:03:40.220223	false
185	Constant Removal, Standardization	Test-Budgeted Statistically Equivalent Signature (SES) algorithm	maxK = 3, alpha = 0.05, budget = 3 * nvars	Support Vector Machines (SVM) of type C-SVC	kernel = 'Polynomial Kernel', cost = 0.1, gamma = 10.0, degree = 3	0.808102756152628	00:01:04.64125	false
186	Constant Removal, Standardization	Test-Budgeted Statistically Equivalent Signature (SES) algorithm	maxK = 2, alpha = 0.05, budget = 3 * nvars	Ridge Logistic Regression	lambda = 1.0	0.8786165525295958	00:00:33.33190	false
187	Constant Removal, Standardization	Test-Budgeted Statistically Equivalent Signature (SES) algorithm	maxK = 3, alpha = 0.01, budget = 3 * nvars	Support Vector Machines (SVM) of type C-SVC	kernel = 'Polynomial Kernel', cost = 1.0, gamma = 0.1, degree = 3	0.7800109452922752	00:00:41.41205	false
188	Constant Removal, Standardization	LASSO Feature Selection	penalty = 1.5	Classification Random Forests with Deviance splitting criterion	ntrees = 500, minimum leaf size = 4	0.9332489406018819	00:03:39.219788	false
189	Constant Removal, Standardization	Test-Budgeted Statistically Equivalent Signature (SES) algorithm	maxK = 2, alpha = 0.05, budget = 3 * nvars	Support Vector Machines (SVM) of type C-SVC	kernel = 'Radial Basis Function Kernel', cost = 10.0, gamma = 1.0	0.8845864990366269	00:00:33.33193	false
190	Constant Removal, Standardization	Test-Budgeted Statistically Equivalent	maxK = 3, alpha = 0.05, budget = 3 * nvars	Classification Decision Tree with Deviance splitting	minimum leaf size = 4, alpha = 0.01	0.8234667612864542	00:01:04.64127	false

Configuration	Preprocessing	Name	Hyperparams	Name	Hyperparams	Performance (unadjusted)	Time (miliseconds)	Dropped
		Signature (SES) algorithm		criterion				
191	Constant Removal, Standardization	LASSO Feature Selection	penalty = 1.0	Classification Random Forests with Deviance splitting criterion	ntrees = 100, minimum leaf size = 4	0.9359239794022403	00:03:40.220423	false
192	Constant Removal, Standardization	Test-Budgeted Statistically Equivalent Signature (SES) algorithm	maxK = 2, alpha = 0.05, budget = 3 * nvars	Support Vector Machines (SVM) of type C-SVC	kernel = 'Polynomial Kernel', cost = 0.01, gamma = 0.01, degree = 2	0.8947077716259301	00:00:33.33190	false
193	Constant Removal, Standardization	Test-Budgeted Statistically Equivalent Signature (SES) algorithm	maxK = 3, alpha = 0.01, budget = 3 * nvars	Support Vector Machines (SVM) of type C-SVC	kernel = 'Polynomial Kernel', cost = 10.0, gamma = 0.01, degree = 2	0.8569511974243432	00:00:41.41205	false
194	Constant Removal, Standardization	LASSO Feature Selection	penalty = 1.5	Support Vector Machines (SVM) of type C-SVC	kernel = 'Polynomial Kernel', cost = 1.0, gamma = 1.0, degree = 3	0.5	00:03:39.219719	false
195	Constant Removal, Standardization	Test-Budgeted Statistically Equivalent Signature (SES) algorithm	maxK = 2, alpha = 0.05, budget = 3 * nvars	Support Vector Machines (SVM) of type C-SVC	kernel = 'Linear Kernel', cost = 0.01	0.889633278060388	00:00:33.33190	false
196	Constant Removal, Standardization	LASSO Feature Selection	penalty = 0.5	Support Vector Machines (SVM) of type C-SVC	kernel = 'Radial Basis Function Kernel', cost = 0.1, gamma = 0.1	0.92506105006105	00:03:40.220182	false
197	Constant Removal, Standardization	Test-Budgeted Statistically Equivalent Signature (SES) algorithm	maxK = 3, alpha = 0.01, budget = 3 * nvars	Classification Decision Tree with Deviance splitting criterion	minimum leaf size = 3, alpha = 0.01	0.8101001548891574	00:00:41.41208	false
198	Constant Removal, Standardization	Test-Budgeted Statistically Equivalent Signature (SES) algorithm	maxK = 2, alpha = 0.01, budget = 3 * nvars	Classification Random Forests with Deviance splitting criterion	ntrees = 500, minimum leaf size = 4	0.8850564752099279	00:00:41.41063	false
199	Constant Removal, Standardization	Test-Budgeted Statistically Equivalent Signature (SES) algorithm	maxK = 3, alpha = 0.01, budget = 3 * nvars	Classification Decision Tree with Deviance splitting criterion	minimum leaf size = 4, alpha = 0.01	0.8234667612864542	00:00:41.41207	false
200	Constant Removal, Standardization	Test-Budgeted Statistically Equivalent Signature (SES) algorithm	maxK = 3, alpha = 0.05, budget = 3 * nvars	Support Vector Machines (SVM) of type C-SVC	kernel = 'Polynomial Kernel', cost = 10.0, gamma = 0.01, degree = 2	0.8569511974243432	00:01:04.64124	false

Configuration	Preprocessing	Name	Hyperparams	Name	Hyperparams	Performance (unadjusted)	Time (miliseconds)	Dropped
201	Constant Removal, Standardization	Test-Budgeted Statistically Equivalent Signature (SES) algorithm	maxK = 2, alpha = 0.01, budget = 3 * nvars	Support Vector Machines (SVM) of type C-SVC	kernel = 'Radial Basis Function Kernel', cost = 10.0, gamma = 1.0	0.8845864990366269	00:00:40.40984	false
202	Constant Removal, Standardization	LASSO Feature Selection	penalty = 1.0	Classification Random Forests with Deviance splitting criterion	ntrees = 500, minimum leaf size = 2	0.9325334370091403	00:03:40.220450	false
203	Constant Removal, Standardization	Test-Budgeted Statistically Equivalent Signature (SES) algorithm	maxK = 2, alpha = 0.01, budget = 3 * nvars	Support Vector Machines (SVM) of type C-SVC	kernel = 'Polynomial Kernel', cost = 0.01, gamma = 0.01, degree = 2	0.8947077716259301	00:00:40.40981	false
204	Constant Removal, Standardization	Test-Budgeted Statistically Equivalent Signature (SES) algorithm	maxK = 2, alpha = 0.01, budget = 3 * nvars	Support Vector Machines (SVM) of type C-SVC	kernel = 'Polynomial Kernel', cost = 10.0, gamma = 0.1, degree = 2	0.7732376674192531	00:00:40.40981	false
205	Constant Removal, Standardization	Test-Budgeted Statistically Equivalent Signature (SES) algorithm	maxK = 2, alpha = 0.01, budget = 3 * nvars	Classification Random Forests with Deviance splitting criterion	ntrees = 500, minimum leaf size = 4	0.8850564752099279	00:00:41.41064	false
206	Constant Removal, Standardization	Test-Budgeted Statistically Equivalent Signature (SES) algorithm	maxK = 2, alpha = 0.05, budget = 3 * nvars	Support Vector Machines (SVM) of type C-SVC	kernel = 'Polynomial Kernel', cost = 10.0, gamma = 10.0, degree = 2	0.6222168354521296	00:00:33.33190	false
207	Constant Removal, Standardization	LASSO Feature Selection	penalty = 1.5	Support Vector Machines (SVM) of type C-SVC	kernel = 'Radial Basis Function Kernel', cost = 10.0, gamma = 0.1	0.9194431953383361	00:03:39.219720	false
208	Constant Removal, Standardization	LASSO Feature Selection	penalty = 1.0	Support Vector Machines (SVM) of type C-SVC	kernel = 'Polynomial Kernel', cost = 0.1, gamma = 0.01, degree = 3	0.893186984938903	00:03:40.220410	false
209	Constant Removal, Standardization	Test-Budgeted Statistically Equivalent Signature (SES) algorithm	maxK = 3, alpha = 0.01, budget = 3 * nvars	Ridge Logistic Regression	lambda = 1.0	0.8822838499948474	00:00:41.41205	false
210	Constant Removal, Standardization	LASSO Feature Selection	penalty = 1.0	Support Vector Machines (SVM) of type C-SVC	kernel = 'Polynomial Kernel', cost = 0.1, gamma = 1.0, degree = 3	0.6952345040580334	00:03:40.220410	false
211	Constant Removal, Standardization	Test-Budgeted Statistically Equivalent Signature (SES) algorithm	maxK = 3, alpha = 0.01, budget = 3 * nvars	Support Vector Machines (SVM) of type C-SVC	kernel = 'Polynomial Kernel', cost = 0.1, gamma = 0.01, degree = 2	0.8852028548320109	00:00:41.41205	false

Configuration	Preprocessing	Name	Hyperparams	Name	Hyperparams	Performance (unadjusted)	Time (miliseconds)	Dropped
212	Constant Removal, Standardization	LASSO Feature Selection	penalty = 0.5	Support Vector Machines (SVM) of type C-SVC	kernel = 'Radial Basis Function Kernel', cost = 1.0, gamma = 0.1	0.9212192680862755	00:03:40.220182	false
213	Constant Removal, Standardization	LASSO Feature Selection	penalty = 0.5	Support Vector Machines (SVM) of type C-SVC	kernel = 'Polynomial Kernel', cost = 0.01, gamma = 1.0, degree = 2	0.9046069375353261	00:03:40.220180	false
214	Constant Removal, Standardization	Test-Budgeted Statistically Equivalent Signature (SES) algorithm	maxK = 3, alpha = 0.05, budget = 3 * nvars	Support Vector Machines (SVM) of type C-SVC	kernel = 'Linear Kernel', cost = 10.0	0.8948229235953021	00:01:04.64131	false
215	Constant Removal, Standardization	Test-Budgeted Statistically Equivalent Signature (SES) algorithm	maxK = 2, alpha = 0.01, budget = 3 * nvars	Support Vector Machines (SVM) of type C-SVC	kernel = 'Polynomial Kernel', cost = 0.1, gamma = 10.0, degree = 2	0.6222168354521296	00:00:40.40978	false
216	Constant Removal, Standardization	LASSO Feature Selection	penalty = 0.5	Support Vector Machines (SVM) of type C-SVC	kernel = 'Linear Kernel', cost = 1.0	0.9249650250289635	00:03:40.220181	false
217	Constant Removal, Standardization	Test-Budgeted Statistically Equivalent Signature (SES) algorithm	maxK = 2, alpha = 0.01, budget = 3 * nvars	Classification Random Forests with Deviance splitting criterion	ntrees = 500, minimum leaf size = 3	0.8792075358571522	00:00:41.41033	false
218	Constant Removal, Standardization	LASSO Feature Selection	penalty = 1.0	Classification Random Forests with Deviance splitting criterion	ntrees = 100, minimum leaf size = 4	0.9359239794022403	00:03:40.220422	false
219	Constant Removal, Standardization	LASSO Feature Selection	penalty = 1.0	Support Vector Machines (SVM) of type C-SVC	kernel = 'Radial Basis Function Kernel', cost = 1.0, gamma = 10.0	0.8695160338382846	00:03:40.220413	false
220	Constant Removal, Standardization	LASSO Feature Selection	penalty = 1.5	Classification Random Forests with Deviance splitting criterion	ntrees = 500, minimum leaf size = 4	0.9332489406018819	00:03:39.219785	false
221	Constant Removal, Standardization	LASSO Feature Selection	penalty = 0.5	Support Vector Machines (SVM) of type C-SVC	kernel = 'Polynomial Kernel', cost = 1.0, gamma = 0.01, degree = 3	0.9176979598974483	00:03:40.220180	false
222	Constant Removal, Standardization	LASSO Feature Selection	penalty = 1.0	Support Vector Machines (SVM) of type C-SVC	kernel = 'Radial Basis Function Kernel', cost = 0.01, gamma = 10.0	0.868540169691065	00:03:40.220412	false
223	Constant Removal, Standardization	Test-Budgeted Statistically Equivalent Signature (SES)	maxK = 3, alpha = 0.01, budget = 3 * nvars	Ridge Logistic Regression	lambda = 10.0	0.8888330694596679	00:00:41.41205	false

Configuration	Preprocessing	Name	Hyperparams	Name	Hyperparams	Performance (unadjusted)	Time (miliseconds)	Dropped
		algorithm						
224	Constant Removal, Standardization	LASSO Feature Selection	penalty = 0.5	Support Vector Machines (SVM) of type C-SVC	kernel = 'Polynomial Kernel', cost = 0.1, gamma = 0.1, degree = 2	0.7169107888417352	00:03:40.220180	false
225	Constant Removal, Standardization	LASSO Feature Selection	penalty = 0.5	Support Vector Machines (SVM) of type C-SVC	kernel = 'Radial Basis Function Kernel', cost = 0.1, gamma = 0.01	0.9228516936317448	00:03:40.220181	false
226	Constant Removal, Standardization	Test-Budgeted Statistically Equivalent Signature (SES) algorithm	maxK = 3, alpha = 0.05, budget = 3 * nvars	Support Vector Machines (SVM) of type C-SVC	kernel = 'Polynomial Kernel', cost = 0.1, gamma = 0.01, degree = 2	0.8852028548320109	00:01:04.64125	false
227	Constant Removal, Standardization	Test-Budgeted Statistically Equivalent Signature (SES) algorithm	maxK = 3, alpha = 0.01, budget = 3 * nvars	Support Vector Machines (SVM) of type C-SVC	kernel = 'Radial Basis Function Kernel', cost = 0.01, gamma = 0.01	0.8800920591201922	00:00:41.41206	false
228	Constant Removal, Standardization	Test-Budgeted Statistically Equivalent Signature (SES) algorithm	maxK = 3, alpha = 0.05, budget = 3 * nvars	Classification Random Forests with Deviance splitting criterion	ntrees = 100, minimum leaf size = 4	0.8857520711740662	00:01:04.64138	false
229	Constant Removal, Standardization	LASSO Feature Selection	penalty = 1.5	Classification Random Forests with Deviance splitting criterion	ntrees = 100, minimum leaf size = 4	0.9317800698874866	00:03:39.219725	false
230	Constant Removal, Standardization	LASSO Feature Selection	penalty = 1.0	Classification Random Forests with Deviance splitting criterion	ntrees = 100, minimum leaf size = 3	0.9316902903859425	00:03:40.220417	false
231	Constant Removal, Standardization	LASSO Feature Selection	penalty = 1.5	Support Vector Machines (SVM) of type C-SVC	kernel = 'Polynomial Kernel', cost = 0.1, gamma = 10.0, degree = 3	0.8004143128823435	00:03:39.219719	false
232	Constant Removal, Standardization	LASSO Feature Selection	penalty = 0.5	Ridge Logistic Regression	lambda = 10.0	0.9245738986787582	00:03:40.220179	false
233	Constant Removal, Standardization	Test-Budgeted Statistically Equivalent Signature (SES) algorithm	maxK = 3, alpha = 0.05, budget = 3 * nvars	Classification Random Forests with Deviance splitting criterion	ntrees = 500, minimum leaf size = 4	0.8905763531722611	00:01:04.64196	false
234	Constant Removal, Standardization	Test-Budgeted Statistically Equivalent Signature (SES) algorithm	maxK = 3, alpha = 0.01, budget = 3 * nvars	Classification Random Forests with Deviance splitting criterion	ntrees = 100, minimum leaf size = 4	0.8803559327856002	00:00:41.41212	false

Configuration	Preprocessing	Name	Hyperparams	Name	Hyperparams	Performance (unadjusted)	Time (miliseconds)	Dropped
235	Constant Removal, Standardization	Test-Budgeted Statistically Equivalent Signature (SES) algorithm	maxK = 3, alpha = 0.05, budget = 3 * nvars	Support Vector Machines (SVM) of type C-SVC	kernel = 'Polynomial Kernel', cost = 0.1, gamma = 1.0, degree = 3	0.7821925871797993	00:01:04.64124	false
236	Constant Removal, Standardization	Test-Budgeted Statistically Equivalent Signature (SES) algorithm	maxK = 2, alpha = 0.01, budget = 3 * nvars	Classification Decision Tree with Deviance splitting criterion	minimum leaf size = 3, alpha = 0.01	0.7912470450833622	00:00:40.40983	false
237	Constant Removal, Standardization	LASSO Feature Selection	penalty = 1.5	Support Vector Machines (SVM) of type C-SVC	kernel = 'Radial Basis Function Kernel', cost = 0.01, gamma = 0.1	0.9289274238123344	00:03:39.219720	false
238	Constant Removal, Standardization	Test-Budgeted Statistically Equivalent Signature (SES) algorithm	maxK = 3, alpha = 0.01, budget = 3 * nvars	Support Vector Machines (SVM) of type C-SVC	kernel = 'Polynomial Kernel', cost = 1.0, gamma = 1.0, degree = 3	0.7662262786943094	00:00:41.41205	false
239	Constant Removal, Standardization	Test-Budgeted Statistically Equivalent Signature (SES) algorithm	maxK = 3, alpha = 0.05, budget = 3 * nvars	Support Vector Machines (SVM) of type C-SVC	kernel = 'Linear Kernel', cost = 0.01	0.8955153967941691	00:01:04.64125	false
240	Constant Removal, Standardization	LASSO Feature Selection	penalty = 1.5	Support Vector Machines (SVM) of type C-SVC	kernel = 'Polynomial Kernel', cost = 0.1, gamma = 1.0, degree = 3	0.6796409756767814	00:03:39.219719	false
241	Constant Removal, Standardization	Test-Budgeted Statistically Equivalent Signature (SES) algorithm	maxK = 3, alpha = 0.05, budget = 3 * nvars	Support Vector Machines (SVM) of type C-SVC	kernel = 'Polynomial Kernel', cost = 0.01, gamma = 0.01, degree = 2	0.8972555577414912	00:01:04.64125	false
242	Constant Removal, Standardization	Test-Budgeted Statistically Equivalent Signature (SES) algorithm	maxK = 2, alpha = 0.01, budget = 3 * nvars	Classification Random Forests with Deviance splitting criterion	ntrees = 100, minimum leaf size = 4	0.8818525180417763	00:00:40.40999	false
243	Constant Removal, Standardization	LASSO Feature Selection	penalty = 1.5	Support Vector Machines (SVM) of type C-SVC	kernel = 'Polynomial Kernel', cost = 1.0, gamma = 0.1, degree = 3	0.5	00:03:39.219719	false
244	Constant Removal, Standardization	LASSO Feature Selection	penalty = 0.5	Support Vector Machines (SVM) of type C-SVC	kernel = 'Polynomial Kernel', cost = 1.0, gamma = 0.01, degree = 2	0.9159140802363309	00:03:40.220180	false
245	Constant Removal, Standardization	Test-Budgeted Statistically Equivalent Signature (SES) algorithm	maxK = 2, alpha = 0.01, budget = 3 * nvars	Support Vector Machines (SVM) of type C-SVC	kernel = 'Polynomial Kernel', cost = 0.1, gamma = 0.01, degree = 3	0.8502306162152712	00:00:40.40978	false
246	Constant Removal,	LASSO Feature	penalty = 1.0	Support Vector	kernel = 'Radial Basis	0.9163231624868453	00:03:40.220412	false

Configuration	Preprocessing	Name	Hyperparams	Name	Hyperparams	Performance (unadjusted)	Time (miliseconds)	Dropped
	Standardization	Selection		Machines (SVM) of type C-SVC	Function Kernel', cost = 10.0, gamma = 1.0			
247	Constant Removal, Standardization	Test-Budgeted Statistically Equivalent Signature (SES) algorithm	maxK = 3, alpha = 0.05, budget = 3 * nvars	Support Vector Machines (SVM) of type C-SVC	kernel = 'Polynomial Kernel', cost = 1.0, gamma = 0.1, degree = 3	0.7800109452922752	00:01:04.64125	false
248	Constant Removal, Standardization	LASSO Feature Selection	penalty = 1.5	Support Vector Machines (SVM) of type C-SVC	kernel = 'Polynomial Kernel', cost = 0.01, gamma = 0.01, degree = 2	0.9253381954788606	00:03:39.219719	false
249	Constant Removal, Standardization	LASSO Feature Selection	penalty = 0.5	Classification Random Forests with Deviance splitting criterion	ntrees = 500, minimum leaf size = 3	0.9310438779748242	00:03:40.220252	false
250	Constant Removal, Standardization	LASSO Feature Selection	penalty = 1.0	Support Vector Machines (SVM) of type C-SVC	kernel = 'Polynomial Kernel', cost = 0.01, gamma = 0.1, degree = 2	0.9259947568771097	00:03:40.220410	false
251	Constant Removal, Standardization	Test-Budgeted Statistically Equivalent Signature (SES) algorithm	maxK = 2, alpha = 0.05, budget = 3 * nvars	Support Vector Machines (SVM) of type C-SVC	kernel = 'Polynomial Kernel', cost = 10.0, gamma = 0.1, degree = 2	0.7732376674192531	00:00:33.33190	false
252	Constant Removal, Standardization	LASSO Feature Selection	penalty = 1.0	Support Vector Machines (SVM) of type C-SVC	kernel = 'Radial Basis Function Kernel', cost = 0.01, gamma = 1.0	0.9204854182475666	00:03:40.220412	false
253	Constant Removal, Standardization	Test-Budgeted Statistically Equivalent Signature (SES) algorithm	maxK = 2, alpha = 0.01, budget = 3 * nvars	Support Vector Machines (SVM) of type C-SVC	kernel = 'Polynomial Kernel', cost = 0.1, gamma = 0.01, degree = 2	0.8721469635791887	00:00:40.40979	false
254	Constant Removal, Standardization	Test-Budgeted Statistically Equivalent Signature (SES) algorithm	maxK = 2, alpha = 0.05, budget = 3 * nvars	Support Vector Machines (SVM) of type C-SVC	kernel = 'Polynomial Kernel', cost = 1.0, gamma = 0.1, degree = 3	0.7463830571247452	00:00:33.33190	false
255	Constant Removal, Standardization	Test-Budgeted Statistically Equivalent Signature (SES) algorithm	maxK = 3, alpha = 0.05, budget = 3 * nvars	Classification Random Forests with Deviance splitting criterion	ntrees = 100, minimum leaf size = 4	0.8803559327856002	00:01:04.64132	false
256	Constant Removal, Standardization	Test-Budgeted Statistically Equivalent Signature (SES) algorithm	maxK = 2, alpha = 0.05, budget = 3 * nvars	Classification Decision Tree with Deviance splitting criterion	minimum leaf size = 3, alpha = 0.01	0.7912470450833622	00:00:33.33193	false
257	Constant Removal, Standardization	LASSO Feature Selection	penalty = 1.0	Classification Random Forests with Deviance splitting	ntrees = 100, minimum leaf size = 2	0.9291807581449525	00:03:40.220417	false

Configuration	Preprocessing	Name	Hyperparams	Name	Hyperparams	Performance (unadjusted)	Time (miliseconds)	Dropped
				criterion				
258	Constant Removal, Standardization	Test-Budgeted Statistically Equivalent Signature (SES) algorithm	maxK = 2, alpha = 0.01, budget = 3 * nvars	Support Vector Machines (SVM) of type C-SVC	kernel = 'Linear Kernel', cost = 0.01	0.889633278060388	00:00:40.40979	false
259	Constant Removal, Standardization	LASSO Feature Selection	penalty = 1.0	Support Vector Machines (SVM) of type C-SVC	kernel = 'Polynomial Kernel', cost = 10.0, gamma = 10.0, degree = 2	0.5255422681893269	00:03:40.220410	false
260	Constant Removal, Standardization	LASSO Feature Selection	penalty = 1.0	Support Vector Machines (SVM) of type C-SVC	kernel = 'Polynomial Kernel', cost = 0.01, gamma = 10.0, degree = 2	0.6828906032870228	00:03:40.220410	false
261	Constant Removal, Standardization	Test-Budgeted Statistically Equivalent Signature (SES) algorithm	maxK = 2, alpha = 0.05, budget = 3 * nvars	Support Vector Machines (SVM) of type C-SVC	kernel = 'Polynomial Kernel', cost = 0.1, gamma = 0.01, degree = 3	0.8502306162152712	00:00:33.33190	false
262	Constant Removal, Standardization	Test-Budgeted Statistically Equivalent Signature (SES) algorithm	maxK = 3, alpha = 0.01, budget = 3 * nvars	Classification Random Forests with Deviance splitting criterion	ntrees = 500, minimum leaf size = 3	0.8859566122993233	00:00:41.41248	false
263	Constant Removal, Standardization	LASSO Feature Selection	penalty = 0.5	Support Vector Machines (SVM) of type C-SVC	kernel = 'Polynomial Kernel', cost = 1.0, gamma = 10.0, degree = 2	0.5255422681893269	00:03:40.220180	false
264	Constant Removal, Standardization	Test-Budgeted Statistically Equivalent Signature (SES) algorithm	maxK = 3, alpha = 0.01, budget = 3 * nvars	Support Vector Machines (SVM) of type C-SVC	kernel = 'Linear Kernel', cost = 10.0	0.8948229235953021	00:00:41.41211	false
265	Constant Removal, Standardization	LASSO Feature Selection	penalty = 1.5	Support Vector Machines (SVM) of type C-SVC	kernel = 'Radial Basis Function Kernel', cost = 0.01, gamma = 0.01	0.922478523181848	00:03:39.219720	false
266	Constant Removal, Standardization	Test-Budgeted Statistically Equivalent Signature (SES) algorithm	maxK = 2, alpha = 0.05, budget = 3 * nvars	Support Vector Machines (SVM) of type C-SVC	kernel = 'Polynomial Kernel', cost = 0.1, gamma = 1.0, degree = 3	0.7661427447233073	00:00:33.33190	false
267	Constant Removal, Standardization	LASSO Feature Selection	penalty = 1.5	Support Vector Machines (SVM) of type C-SVC	kernel = 'Polynomial Kernel', cost = 0.1, gamma = 0.01, degree = 2	0.9114820956253182	00:03:39.219719	false
268	Constant Removal, Standardization	LASSO Feature Selection	penalty = 0.5	Classification Random Forests with Deviance splitting criterion	ntrees = 500, minimum leaf size = 4	0.9328937260522939	00:03:40.220257	false

Configuration	Preprocessing	Name	Hyperparams	Name	Hyperparams	Performance (unadjusted)	Time (miliseconds)	Dropped
269	Constant Removal, Standardization	Test-Budgeted Statistically Equivalent Signature (SES) algorithm	maxK = 3, alpha = 0.01, budget = 3 * nvars	Support Vector Machines (SVM) of type C-SVC	kernel = 'Polynomial Kernel', cost = 0.1, gamma = 10.0, degree = 3	0.808102756152628	00:00:41.41205	false
270	Constant Removal, Standardization	Test-Budgeted Statistically Equivalent Signature (SES) algorithm	maxK = 3, alpha = 0.05, budget = 3 * nvars	Ridge Logistic Regression	lambda = 1.0	0.8822838499948474	00:01:04.64125	false
271	Constant Removal, Standardization	LASSO Feature Selection	penalty = 1.5	Ridge Logistic Regression	lambda = 1.0	0.9226811125788107	00:03:39.219719	false
272	Constant Removal, Standardization	LASSO Feature Selection	penalty = 0.5	Classification Random Forests with Deviance splitting criterion	ntrees = 100, minimum leaf size = 2	0.9337548285758005	00:03:40.220195	false
273	Constant Removal, Standardization	LASSO Feature Selection	penalty = 0.5	Support Vector Machines (SVM) of type C-SVC	kernel = 'Polynomial Kernel', cost = 1.0, gamma = 10.0, degree = 3	0.7986382401344039	00:03:40.220180	false
274	Constant Removal, Standardization	LASSO Feature Selection	penalty = 1.0	Support Vector Machines (SVM) of type C-SVC	kernel = 'Polynomial Kernel', cost = 10.0, gamma = 0.1, degree = 3	0.5	00:03:40.220410	false
275	Constant Removal, Standardization	LASSO Feature Selection	penalty = 0.5	Support Vector Machines (SVM) of type C-SVC	kernel = 'Polynomial Kernel', cost = 0.01, gamma = 10.0, degree = 3	0.855061534089667	00:03:40.220180	false
276	Constant Removal, Standardization	Test-Budgeted Statistically Equivalent Signature (SES) algorithm	maxK = 3, alpha = 0.01, budget = 3 * nvars	Support Vector Machines (SVM) of type C-SVC	kernel = 'Polynomial Kernel', cost = 0.1, gamma = 10.0, degree = 2	0.6303984804624191	00:00:41.41205	false
277	Constant Removal, Standardization	LASSO Feature Selection	penalty = 1.5	Support Vector Machines (SVM) of type C-SVC	kernel = 'Linear Kernel', cost = 0.01	0.9253202395785518	00:03:39.219719	false
278	Constant Removal, Standardization	LASSO Feature Selection	penalty = 1.0	Classification Random Forests with Deviance splitting criterion	ntrees = 100, minimum leaf size = 2	0.9337548285758005	00:03:40.220423	false
279	Constant Removal, Standardization	Test-Budgeted Statistically Equivalent Signature (SES) algorithm	maxK = 3, alpha = 0.01, budget = 3 * nvars	Support Vector Machines (SVM) of type C-SVC	kernel = 'Polynomial Kernel', cost = 0.01, gamma = 0.01, degree = 2	0.8972555577414912	00:00:41.41205	false
280	Constant Removal, Standardization	Test-Budgeted Statistically Equivalent Signature (SES) algorithm	maxK = 2, alpha = 0.05, budget = 3 * nvars	Classification Random Forests with Deviance splitting criterion	ntrees = 100, minimum leaf size = 4	0.8818525180417763	00:00:33.33204	false

Configuration	Preprocessing	Name	Hyperparams	Name	Hyperparams	Performance (unadjusted)	Time (miliseconds)	Dropped
281	Constant Removal, Standardization	LASSO Feature Selection	penalty = 0.5	Classification Random Forests with Deviance splitting criterion	ntrees = 100, minimum leaf size = 3	0.93945504623254	00:03:40.220195	false
282	Constant Removal, Standardization	Test-Budgeted Statistically Equivalent Signature (SES) algorithm	maxK = 2, alpha = 0.05, budget = 3 * nvars	Classification Random Forests with Deviance splitting criterion	ntrees = 500, minimum leaf size = 4	0.8850564752099279	00:00:33.33262	false
283	Constant Removal, Standardization	LASSO Feature Selection	penalty = 1.0	Support Vector Machines (SVM) of type C-SVC	kernel = 'Radial Basis Function Kernel', cost = 10.0, gamma = 10.0	0.8708498449547043	00:03:40.220413	false
284	Constant Removal, Standardization	Test-Budgeted Statistically Equivalent Signature (SES) algorithm	maxK = 3, alpha = 0.01, budget = 3 * nvars	Support Vector Machines (SVM) of type C-SVC	kernel = 'Radial Basis Function Kernel', cost = 0.01, gamma = 0.1	0.8879153668156226	00:00:41.41206	false
285	Constant Removal, Standardization	Test-Budgeted Statistically Equivalent Signature (SES) algorithm	maxK = 2, alpha = 0.01, budget = 3 * nvars	Ridge Logistic Regression	lambda = 1.0	0.8786165525295958	00:00:40.40979	false
286	Constant Removal, Standardization	Test-Budgeted Statistically Equivalent Signature (SES) algorithm	maxK = 2, alpha = 0.05, budget = 3 * nvars	Support Vector Machines (SVM) of type C-SVC	kernel = 'Polynomial Kernel', cost = 10.0, gamma = 0.01, degree = 3	0.8834767463284089	00:00:33.33190	false
287	Constant Removal, Standardization	LASSO Feature Selection	penalty = 0.5	Classification Random Forests with Deviance splitting criterion	ntrees = 500, minimum leaf size = 2	0.9332309847015732	00:03:40.220265	false
288	Constant Removal, Standardization	Test-Budgeted Statistically Equivalent Signature (SES) algorithm	maxK = 2, alpha = 0.05, budget = 3 * nvars	Support Vector Machines (SVM) of type C-SVC	kernel = 'Linear Kernel', cost = 0.1	0.889970536709667	00:00:33.33191	false
289	Constant Removal, Standardization	Test-Budgeted Statistically Equivalent Signature (SES) algorithm	maxK = 3, alpha = 0.05, budget = 3 * nvars	Classification Random Forests with Deviance splitting criterion	ntrees = 100, minimum leaf size = 4	0.8857520711740662	00:01:04.64138	false
290	Constant Removal, Standardization	Test-Budgeted Statistically Equivalent Signature (SES) algorithm	maxK = 3, alpha = 0.01, budget = 3 * nvars	Classification Random Forests with Deviance splitting criterion	ntrees = 500, minimum leaf size = 2	0.8897023692420112	00:00:41.41279	false
291	Constant Removal, Standardization	Test-Budgeted Statistically Equivalent Signature (SES) algorithm	maxK = 2, alpha = 0.01, budget = 3 * nvars	Support Vector Machines (SVM) of type C-SVC	kernel = 'Linear Kernel', cost = 1.0	0.8896153221600791	00:00:40.40980	false

Configuration	Preprocessing	Name	Hyperparams	Name	Hyperparams	Performance (unadjusted)	Time (miliseconds)	Dropped
292	Constant Removal, Standardization	LASSO Feature Selection	penalty = 1.5	Support Vector Machines (SVM) of type C-SVC	kernel = 'Polynomial Kernel', cost = 10.0, gamma = 1.0, degree = 2	0.5	00:03:39.219719	false
293	Constant Removal, Standardization	Test-Budgeted Statistically Equivalent Signature (SES) algorithm	maxK = 2, alpha = 0.01, budget = 3 * nvars	Classification Random Forests with Deviance splitting criterion	ntrees = 100, minimum leaf size = 4	0.8818525180417763	00:00:40.40992	false
294	Constant Removal, Standardization	Test-Budgeted Statistically Equivalent Signature (SES) algorithm	maxK = 2, alpha = 0.01, budget = 3 * nvars	Support Vector Machines (SVM) of type C-SVC	kernel = 'Radial Basis Function Kernel', cost = 0.01, gamma = 10.0	0.8009791430507543	00:00:40.40983	false
295	Constant Removal, Standardization	LASSO Feature Selection	penalty = 1.5	Classification Random Forests with Deviance splitting criterion	ntrees = 500, minimum leaf size = 2	0.934296628350337	00:03:39.219789	false
296	Constant Removal, Standardization	Test-Budgeted Statistically Equivalent Signature (SES) algorithm	maxK = 2, alpha = 0.05, budget = 3 * nvars	Classification Random Forests with Deviance splitting criterion	ntrees = 500, minimum leaf size = 4	0.8767749017109631	00:00:33.33235	false
297	Constant Removal, Standardization	LASSO Feature Selection	penalty = 0.5	Support Vector Machines (SVM) of type C-SVC	kernel = 'Radial Basis Function Kernel', cost = 0.1, gamma = 1.0	0.9203035171705249	00:03:40.220182	false
298	Constant Removal, Standardization	LASSO Feature Selection	penalty = 0.5	Classification Decision Tree with Deviance splitting criterion	minimum leaf size = 2, alpha = 0.05	0.8422263833069459	00:03:40.220183	false
299	Constant Removal, Standardization	LASSO Feature Selection	penalty = 1.0	Support Vector Machines (SVM) of type C-SVC	kernel = 'Linear Kernel', cost = 10.0	0.9249650250289635	00:03:40.220414	false
300	Constant Removal, Standardization	Test-Budgeted Statistically Equivalent Signature (SES) algorithm	maxK = 2, alpha = 0.01, budget = 3 * nvars	Support Vector Machines (SVM) of type C-SVC	kernel = 'Polynomial Kernel', cost = 10.0, gamma = 1.0, degree = 3	0.7255920762954012	00:00:40.40980	false
301	Constant Removal, Standardization	Test-Budgeted Statistically Equivalent Signature (SES) algorithm	maxK = 3, alpha = 0.01, budget = 3 * nvars	Support Vector Machines (SVM) of type C-SVC	kernel = 'Linear Kernel', cost = 0.1	0.8955153967941691	00:00:41.41205	false
302	Constant Removal, Standardization	LASSO Feature Selection	penalty = 1.5	Classification Random Forests with Deviance splitting criterion	ntrees = 500, minimum leaf size = 3	0.9339722511078009	00:03:39.219758	false
303	Constant Removal, Standardization	LASSO Feature Selection	penalty = 0.5	Classification Random Forests with Deviance splitting	ntrees = 100, minimum leaf size = 3	0.93945504623254	00:03:40.220193	false

Configuration	Preprocessing	Name	Hyperparams	Name	Hyperparams	Performance (unadjusted)	Time (miliseconds)	Dropped
				criterion				
304	Constant Removal, Standardization	Test-Budgeted Statistically Equivalent Signature (SES) algorithm	maxK = 3, alpha = 0.05, budget = 3 * nvars	Support Vector Machines (SVM) of type C-SVC	kernel = 'Radial Basis Function Kernel', cost = 0.01, gamma = 0.1	0.8879153668156226	00:01:04.64127	false
305	Constant Removal, Standardization	LASSO Feature Selection	penalty = 0.5	Support Vector Machines (SVM) of type C-SVC	kernel = 'Polynomial Kernel', cost = 0.01, gamma = 10.0, degree = 2	0.6828906032870228	00:03:40.220180	false
306	Constant Removal, Standardization	LASSO Feature Selection	penalty = 0.5	Support Vector Machines (SVM) of type C-SVC	kernel = 'Polynomial Kernel', cost = 0.01, gamma = 1.0, degree = 3	0.8898553847402951	00:03:40.220180	false
307	Constant Removal, Standardization	Test-Budgeted Statistically Equivalent Signature (SES) algorithm	maxK = 2, alpha = 0.05, budget = 3 * nvars	Support Vector Machines (SVM) of type C-SVC	kernel = 'Polynomial Kernel', cost = 10.0, gamma = 10.0, degree = 3	0.778636538227331	00:00:33.33191	false
308	Constant Removal, Standardization	LASSO Feature Selection	penalty = 1.0	Support Vector Machines (SVM) of type C-SVC	kernel = 'Polynomial Kernel', cost = 0.1, gamma = 1.0, degree = 2	0.6924739795583786	00:03:40.220410	false
309	Constant Removal, Standardization	Test-Budgeted Statistically Equivalent Signature (SES) algorithm	maxK = 2, alpha = 0.01, budget = 3 * nvars	Support Vector Machines (SVM) of type C-SVC	kernel = 'Radial Basis Function Kernel', cost = 0.01, gamma = 0.01	0.8753591180061768	00:00:40.40981	false
310	Constant Removal, Standardization	Test-Budgeted Statistically Equivalent Signature (SES) algorithm	maxK = 3, alpha = 0.01, budget = 3 * nvars	Support Vector Machines (SVM) of type C-SVC	kernel = 'Polynomial Kernel', cost = 1.0, gamma = 0.01, degree = 3	0.8657648120563722	00:00:41.41205	false
311	Constant Removal, Standardization	LASSO Feature Selection	penalty = 1.0	Classification Decision Tree with Deviance splitting criterion	minimum leaf size = 3, alpha = 0.01	0.8953471578151886	00:03:40.220413	false
312	Constant Removal, Standardization	Test-Budgeted Statistically Equivalent Signature (SES) algorithm	maxK = 2, alpha = 0.05, budget = 3 * nvars	Support Vector Machines (SVM) of type C-SVC	kernel = 'Polynomial Kernel', cost = 0.1, gamma = 10.0, degree = 2	0.6222168354521296	00:00:33.33190	false
313	Constant Removal, Standardization	LASSO Feature Selection	penalty = 1.0	Support Vector Machines (SVM) of type C-SVC	kernel = 'Polynomial Kernel', cost = 0.01, gamma = 0.1, degree = 3	0.9277528737247406	00:03:40.220410	false
314	Constant Removal, Standardization	Test-Budgeted Statistically Equivalent Signature (SES) algorithm	maxK = 2, alpha = 0.05, budget = 3 * nvars	Classification Random Forests with Deviance splitting criterion	ntrees = 500, minimum leaf size = 2	0.8868376224514332	00:00:33.33287	false
315	Constant Removal, Standardization	LASSO Feature Selection	penalty = 0.5	Support Vector Machines (SVM) of type	kernel = 'Polynomial Kernel', cost = 0.1, gamma =	0.6827196318884299	00:03:40.220180	false

Configuration	Preprocessing	Name	Hyperparams	Name	Hyperparams	Performance (unadjusted)	Time (miliseconds)	Dropped
				C-SVC	0.1, degree = 3			
316	Constant Removal, Standardization	Test-Budgeted Statistically Equivalent Signature (SES) algorithm	maxK = 3, alpha = 0.05, budget = 3 * nvars	Ridge Logistic Regression	lambda = 10.0	0.8888330694596679	00:01:04.64125	false
317	Constant Removal, Standardization	Test-Budgeted Statistically Equivalent Signature (SES) algorithm	maxK = 3, alpha = 0.05, budget = 3 * nvars	Classification Random Forests with Deviance splitting criterion	ntrees = 500, minimum leaf size = 4	0.8905763531722611	00:01:04.64205	false
318	Constant Removal, Standardization	Test-Budgeted Statistically Equivalent Signature (SES) algorithm	maxK = 3, alpha = 0.05, budget = 3 * nvars	Support Vector Machines (SVM) of type C-SVC	kernel = 'Polynomial Kernel', cost = 0.1, gamma = 0.01, degree = 3	0.8179962620499704	00:01:04.64125	false
319	Constant Removal, Standardization	Test-Budgeted Statistically Equivalent Signature (SES) algorithm	maxK = 2, alpha = 0.05, budget = 3 * nvars	Support Vector Machines (SVM) of type C-SVC	kernel = 'Polynomial Kernel', cost = 1.0, gamma = 0.1, degree = 2	0.7732376674192531	00:00:33.33190	false
320	Constant Removal, Standardization	Test-Budgeted Statistically Equivalent Signature (SES) algorithm	maxK = 2, alpha = 0.01, budget = 3 * nvars	Support Vector Machines (SVM) of type C-SVC	kernel = 'Polynomial Kernel', cost = 0.1, gamma = 1.0, degree = 3	0.7661427447233073	00:00:40.40979	false
321	Constant Removal, Standardization	Test-Budgeted Statistically Equivalent Signature (SES) algorithm	maxK = 2, alpha = 0.01, budget = 3 * nvars	Support Vector Machines (SVM) of type C-SVC	kernel = 'Polynomial Kernel', cost = 0.01, gamma = 1.0, degree = 2	0.8874879383191403	00:00:40.40985	false
322	Constant Removal, Standardization	LASSO Feature Selection	penalty = 1.0	Support Vector Machines (SVM) of type C-SVC	kernel = 'Polynomial Kernel', cost = 1.0, gamma = 1.0, degree = 3	0.5	00:03:40.220410	false
323	Constant Removal, Standardization	LASSO Feature Selection	penalty = 1.5	Support Vector Machines (SVM) of type C-SVC	kernel = 'Radial Basis Function Kernel', cost = 10.0, gamma = 0.01	0.9199920213347325	00:03:39.219720	false
324	Constant Removal, Standardization	Test-Budgeted Statistically Equivalent Signature (SES) algorithm	maxK = 3, alpha = 0.05, budget = 3 * nvars	Support Vector Machines (SVM) of type C-SVC	kernel = 'Polynomial Kernel', cost = 10.0, gamma = 10.0, degree = 3	0.808102756152628	00:01:04.64125	false
325	Constant Removal, Standardization	Test-Budgeted Statistically Equivalent Signature (SES) algorithm	maxK = 2, alpha = 0.01, budget = 3 * nvars	Support Vector Machines (SVM) of type C-SVC	kernel = 'Polynomial Kernel', cost = 1.0, gamma = 1.0, degree = 3	0.7255920762954012	00:00:40.40986	false

Configuration	Preprocessing	Name	Hyperparams	Name	Hyperparams	Performance (unadjusted)	Time (miliseconds)	Dropped
326	Constant Removal, Standardization	LASSO Feature Selection	penalty = 1.5	Support Vector Machines (SVM) of type C-SVC	kernel = 'Radial Basis Function Kernel', cost = 1.0, gamma = 10.0	0.8735600148643627	00:03:39.219721	false
327	Constant Removal, Standardization	Test-Budgeted Statistically Equivalent Signature (SES) algorithm	maxK = 2, alpha = 0.05, budget = 3 * nvars	Support Vector Machines (SVM) of type C-SVC	kernel = 'Polynomial Kernel', cost = 1.0, gamma = 10.0, degree = 2	0.6222168354521296	00:00:33.33190	false
328	Constant Removal, Standardization	LASSO Feature Selection	penalty = 0.5	Classification Random Forests with Deviance splitting criterion	ntrees = 100, minimum leaf size = 2	0.9337548285758005	00:03:40.220195	false
329	Constant Removal, Standardization	Test-Budgeted Statistically Equivalent Signature (SES) algorithm	maxK = 2, alpha = 0.01, budget = 3 * nvars	Support Vector Machines (SVM) of type C-SVC	kernel = 'Polynomial Kernel', cost = 10.0, gamma = 10.0, degree = 2	0.6222168354521296	00:00:40.40986	false
330	Constant Removal, Standardization	LASSO Feature Selection	penalty = 0.5	Classification Decision Tree with Deviance splitting criterion	minimum leaf size = 2, alpha = 0.01	0.8853435744420399	00:03:40.220183	false
331	Constant Removal, Standardization	LASSO Feature Selection	penalty = 1.5	Support Vector Machines (SVM) of type C-SVC	kernel = 'Radial Basis Function Kernel', cost = 1.0, gamma = 0.1	0.9194431953383361	00:03:39.219720	false
332	Constant Removal, Standardization	Test-Budgeted Statistically Equivalent Signature (SES) algorithm	maxK = 2, alpha = 0.01, budget = 3 * nvars	Support Vector Machines (SVM) of type C-SVC	kernel = 'Polynomial Kernel', cost = 1.0, gamma = 0.01, degree = 3	0.8834767463284089	00:00:40.40987	false
333	Constant Removal, Standardization	LASSO Feature Selection	penalty = 1.5	Classification Random Forests with Deviance splitting criterion	ntrees = 100, minimum leaf size = 4	0.9384104812493559	00:03:39.219731	false
334	Constant Removal, Standardization	Test-Budgeted Statistically Equivalent Signature (SES) algorithm	maxK = 3, alpha = 0.05, budget = 3 * nvars	Support Vector Machines (SVM) of type C-SVC	kernel = 'Polynomial Kernel', cost = 0.1, gamma = 10.0, degree = 2	0.6303984804624191	00:01:04.64125	false
335	Constant Removal, Standardization	LASSO Feature Selection	penalty = 1.0	Support Vector Machines (SVM) of type C-SVC	kernel = 'Polynomial Kernel', cost = 1.0, gamma = 1.0, degree = 2	0.5	00:03:40.220410	false
336	Constant Removal, Standardization	LASSO Feature Selection	penalty = 1.5	Support Vector Machines (SVM) of type C-SVC	kernel = 'Radial Basis Function Kernel', cost = 0.1, gamma = 0.1	0.925771479160226	00:03:39.219720	false
337	Constant Removal, Standardization	LASSO Feature Selection	penalty = 0.5	Support Vector Machines (SVM) of type C-SVC	kernel = 'Polynomial Kernel', cost = 1.0, gamma = 0.1, degree = 3	0.5	00:03:40.220179	false

Configuration	Preprocessing	Name	Hyperparams	Name	Hyperparams	Performance (unadjusted)	Time (miliseconds)	Dropped
338	Constant Removal, Standardization	Test-Budgeted Statistically Equivalent Signature (SES) algorithm	maxK = 2, alpha = 0.05, budget = 3 * nvars	Classification Random Forests with Deviance splitting criterion	ntrees = 500, minimum leaf size = 4	0.8850564752099279	00:00:33.33283	false
339	Constant Removal, Standardization	Test-Budgeted Statistically Equivalent Signature (SES) algorithm	maxK = 3, alpha = 0.01, budget = 3 * nvars	Support Vector Machines (SVM) of type C-SVC	kernel = 'Polynomial Kernel', cost = 0.1, gamma = 0.01, degree = 3	0.8179962620499704	00:00:41.41205	false
340	Constant Removal, Standardization	LASSO Feature Selection	penalty = 1.0	Support Vector Machines (SVM) of type C-SVC	kernel = 'Radial Basis Function Kernel', cost = 0.1, gamma = 10.0	0.8688774283403441	00:03:40.220413	false
341	Constant Removal, Standardization	LASSO Feature Selection	penalty = 1.0	Classification Decision Tree with Deviance splitting criterion	minimum leaf size = 3, alpha = 0.05	0.870447008234732	00:03:40.220413	false
342	Constant Removal, Standardization	Test-Budgeted Statistically Equivalent Signature (SES) algorithm	maxK = 2, alpha = 0.01, budget = 3 * nvars	Support Vector Machines (SVM) of type C-SVC	kernel = 'Polynomial Kernel', cost = 1.0, gamma = 10.0, degree = 2	0.6222168354521296	00:00:40.40986	false
343	Constant Removal, Standardization	Test-Budgeted Statistically Equivalent Signature (SES) algorithm	maxK = 3, alpha = 0.01, budget = 3 * nvars	Support Vector Machines (SVM) of type C-SVC	kernel = 'Polynomial Kernel', cost = 0.01, gamma = 1.0, degree = 2	0.8786138201099837	00:00:41.41205	false
344	Constant Removal, Standardization	Test-Budgeted Statistically Equivalent Signature (SES) algorithm	maxK = 3, alpha = 0.01, budget = 3 * nvars	Support Vector Machines (SVM) of type C-SVC	kernel = 'Polynomial Kernel', cost = 0.1, gamma = 1.0, degree = 3	0.7821925871797993	00:00:41.41205	false
345	Constant Removal, Standardization	Test-Budgeted Statistically Equivalent Signature (SES) algorithm	maxK = 3, alpha = 0.05, budget = 3 * nvars	Support Vector Machines (SVM) of type C-SVC	kernel = 'Polynomial Kernel', cost = 10.0, gamma = 0.1, degree = 2	0.8263039887080807	00:01:04.64125	false
346	Constant Removal, Standardization	Test-Budgeted Statistically Equivalent Signature (SES) algorithm	maxK = 3, alpha = 0.01, budget = 3 * nvars	Classification Random Forests with Deviance splitting criterion	ntrees = 100, minimum leaf size = 4	0.8857520711740662	00:00:41.41218	false
347	Constant Removal, Standardization	LASSO Feature Selection	penalty = 0.5	Classification Decision Tree with Deviance splitting criterion	minimum leaf size = 4, alpha = 0.01	0.8924982200237955	00:03:40.220182	false
348	Constant Removal, Standardization	Test-Budgeted Statistically Equivalent Signature (SES) algorithm	maxK = 3, alpha = 0.01, budget = 3 * nvars	Classification Random Forests with Deviance splitting criterion	ntrees = 500, minimum leaf size = 4	0.8848140705557586	00:00:41.41246	false

Configuration	Preprocessing	Name	Hyperparams	Name	Hyperparams	Performance (unadjusted)	Time (miliseconds)	Dropped
349	Constant Removal, Standardization	LASSO Feature Selection	penalty = 0.5	Classification Random Forests with Deviance splitting criterion	ntrees = 500, minimum leaf size = 4	0.9328937260522939	00:03:40.220248	false
350	Constant Removal, Standardization	LASSO Feature Selection	penalty = 1.0	Support Vector Machines (SVM) of type C-SVC	kernel = 'Polynomial Kernel', cost = 10.0, gamma = 0.01, degree = 2	0.9159140802363309	00:03:40.220410	false
351	Constant Removal, Standardization	Test-Budgeted Statistically Equivalent Signature (SES) algorithm	maxK = 3, alpha = 0.05, budget = 3 * nvars	Classification Random Forests with Deviance splitting criterion	ntrees = 500, minimum leaf size = 2	0.8866440110046248	00:01:04.64170	false
352	Constant Removal, Standardization	Test-Budgeted Statistically Equivalent Signature (SES) algorithm	maxK = 3, alpha = 0.01, budget = 3 * nvars	Support Vector Machines (SVM) of type C-SVC	kernel = 'Radial Basis Function Kernel', cost = 0.01, gamma = 10.0	0.8158950313681771	00:00:41.41207	false
353	Constant Removal, Standardization	LASSO Feature Selection	penalty = 1.0	Support Vector Machines (SVM) of type C-SVC	kernel = 'Radial Basis Function Kernel', cost = 10.0, gamma = 0.1	0.9212192680862755	00:03:40.220412	false
354	Constant Removal, Standardization	Test-Budgeted Statistically Equivalent Signature (SES) algorithm	maxK = 2, alpha = 0.05, budget = 3 * nvars	Classification Random Forests with Deviance splitting criterion	ntrees = 100, minimum leaf size = 4	0.8818525180417763	00:00:33.33204	false
355	Constant Removal, Standardization	Test-Budgeted Statistically Equivalent Signature (SES) algorithm	maxK = 3, alpha = 0.05, budget = 3 * nvars	Classification Random Forests with Deviance splitting criterion	ntrees = 100, minimum leaf size = 2	0.8914323812022021	00:01:04.64139	false
356	Constant Removal, Standardization	Test-Budgeted Statistically Equivalent Signature (SES) algorithm	maxK = 3, alpha = 0.05, budget = 3 * nvars	Ridge Logistic Regression	lambda = 0.1	0.8808938291035479	00:01:04.64125	false
357	Constant Removal, Standardization	Test-Budgeted Statistically Equivalent Signature (SES) algorithm	maxK = 2, alpha = 0.01, budget = 3 * nvars	Support Vector Machines (SVM) of type C-SVC	kernel = 'Linear Kernel', cost = 10.0	0.8896153221600791	00:00:40.40994	false
358	Constant Removal, Standardization	Test-Budgeted Statistically Equivalent Signature (SES) algorithm	maxK = 3, alpha = 0.01, budget = 3 * nvars	Support Vector Machines (SVM) of type C-SVC	kernel = 'Polynomial Kernel', cost = 10.0, gamma = 0.01, degree = 3	0.8657648120563722	00:00:41.41205	false
359	Constant Removal, Standardization	LASSO Feature Selection	penalty = 0.5	Classification Decision Tree with Deviance splitting criterion	minimum leaf size = 4, alpha = 0.05	0.8577490639511101	00:03:40.220182	false

Configuration	Preprocessing	Name	Hyperparams	Name	Hyperparams	Performance (unadjusted)	Time (miliseconds)	Dropped
360	Constant Removal, Standardization	LASSO Feature Selection	penalty = 1.0	Classification Random Forests with Deviance splitting criterion	ntrees = 500, minimum leaf size = 4	0.9328937260522939	00:03:40.220476	false
361	Constant Removal, Standardization	Test-Budgeted Statistically Equivalent Signature (SES) algorithm	maxK = 3, alpha = 0.05, budget = 3 * nvars	Support Vector Machines (SVM) of type C-SVC	kernel = 'Radial Basis Function Kernel', cost = 1.0, gamma = 1.0	0.8865920950319928	00:01:04.64128	false
362	Constant Removal, Standardization	Test-Budgeted Statistically Equivalent Signature (SES) algorithm	maxK = 3, alpha = 0.01, budget = 3 * nvars	Support Vector Machines (SVM) of type C-SVC	kernel = 'Polynomial Kernel', cost = 10.0, gamma = 1.0, degree = 3	0.7662262786943094	00:00:41.41205	false
363	Constant Removal, Standardization	Test-Budgeted Statistically Equivalent Signature (SES) algorithm	maxK = 3, alpha = 0.01, budget = 3 * nvars	Support Vector Machines (SVM) of type C-SVC	kernel = 'Polynomial Kernel', cost = 1.0, gamma = 0.1, degree = 2	0.8263039887080807	00:00:41.41205	false
364	Constant Removal, Standardization	Test-Budgeted Statistically Equivalent Signature (SES) algorithm	maxK = 3, alpha = 0.05, budget = 3 * nvars	Support Vector Machines (SVM) of type C-SVC	kernel = 'Polynomial Kernel', cost = 1.0, gamma = 0.01, degree = 3	0.8657648120563722	00:01:04.64124	false
365	Constant Removal, Standardization	LASSO Feature Selection	penalty = 1.0	Support Vector Machines (SVM) of type C-SVC	kernel = 'Linear Kernel', cost = 1.0	0.9249650250289635	00:03:40.220411	false
366	Constant Removal, Standardization	Test-Budgeted Statistically Equivalent Signature (SES) algorithm	maxK = 3, alpha = 0.05, budget = 3 * nvars	Classification Decision Tree with Deviance splitting criterion	minimum leaf size = 4, alpha = 0.05	0.8178631541802899	00:01:04.64127	false
367	Constant Removal, Standardization	Test-Budgeted Statistically Equivalent Signature (SES) algorithm	maxK = 2, alpha = 0.05, budget = 3 * nvars	Support Vector Machines (SVM) of type C-SVC	kernel = 'Radial Basis Function Kernel', cost = 0.01, gamma = 0.1	0.8904654950051369	00:00:33.33192	false
368	Constant Removal, Standardization	Test-Budgeted Statistically Equivalent Signature (SES) algorithm	maxK = 2, alpha = 0.01, budget = 3 * nvars	Classification Random Forests with Deviance splitting criterion	ntrees = 500, minimum leaf size = 4	0.8767749017109631	00:00:41.41021	false
369	Constant Removal, Standardization	Test-Budgeted Statistically Equivalent Signature (SES) algorithm	maxK = 3, alpha = 0.05, budget = 3 * nvars	Classification Random Forests with Deviance splitting criterion	ntrees = 500, minimum leaf size = 4	0.8905763531722611	00:01:04.64199	false
370	Constant Removal, Standardization	LASSO Feature Selection	penalty = 0.5	Support Vector Machines (SVM) of type C-SVC	kernel = 'Radial Basis Function Kernel', cost = 0.01, gamma = 0.1	0.9278617801635706	00:03:40.220182	false

Configuration	Preprocessing	Name	Hyperparams	Name	Hyperparams	Performance (unadjusted)	Time (miliseconds)	Dropped
371	Constant Removal, Standardization	Test-Budgeted Statistically Equivalent Signature (SES) algorithm	maxK = 3, alpha = 0.05, budget = 3 * nvars	Support Vector Machines (SVM) of type C-SVC	kernel = 'Polynomial Kernel', cost = 0.01, gamma = 10.0, degree = 2	0.6660860274990709	00:01:04.64125	false
372	Constant Removal, Standardization	LASSO Feature Selection	penalty = 0.5	Classification Random Forests with Deviance splitting criterion	ntrees = 100, minimum leaf size = 3	0.93945504623254	00:03:40.220192	false
373	Constant Removal, Standardization	LASSO Feature Selection	penalty = 0.5	Support Vector Machines (SVM) of type C-SVC	kernel = 'Radial Basis Function Kernel', cost = 1.0, gamma = 1.0	0.9163231624868453	00:03:40.220182	false
374	Constant Removal, Standardization	LASSO Feature Selection	penalty = 1.0	Classification Random Forests with Deviance splitting criterion	ntrees = 500, minimum leaf size = 3	0.9310438779748242	00:03:40.220478	false
375	Constant Removal, Standardization	LASSO Feature Selection	penalty = 1.0	Classification Random Forests with Deviance splitting criterion	ntrees = 100, minimum leaf size = 4	0.9359239794022403	00:03:40.220423	false
376	Constant Removal, Standardization	LASSO Feature Selection	penalty = 0.5	Support Vector Machines (SVM) of type C-SVC	kernel = 'Polynomial Kernel', cost = 0.1, gamma = 10.0, degree = 3	0.7986382401344039	00:03:40.220180	false
377	Constant Removal, Standardization	Test-Budgeted Statistically Equivalent Signature (SES) algorithm	maxK = 2, alpha = 0.05, budget = 3 * nvars	Support Vector Machines (SVM) of type C-SVC	kernel = 'Radial Basis Function Kernel', cost = 0.01, gamma = 0.01	0.8753591180061768	00:00:33.33192	false
378	Constant Removal, Standardization	Test-Budgeted Statistically Equivalent Signature (SES) algorithm	maxK = 3, alpha = 0.05, budget = 3 * nvars	Classification Random Forests with Deviance splitting criterion	ntrees = 500, minimum leaf size = 2	0.8897023692420112	00:01:04.64208	false
379	Constant Removal, Standardization	LASSO Feature Selection	penalty = 1.5	Support Vector Machines (SVM) of type C-SVC	kernel = 'Polynomial Kernel', cost = 10.0, gamma = 0.1, degree = 3	0.5	00:03:39.219719	false
380	Constant Removal, Standardization	Test-Budgeted Statistically Equivalent Signature (SES) algorithm	maxK = 2, alpha = 0.05, budget = 3 * nvars	Support Vector Machines (SVM) of type C-SVC	kernel = 'Polynomial Kernel', cost = 0.1, gamma = 0.1, degree = 2	0.8272181782411963	00:00:33.33190	false
381	Constant Removal, Standardization	Test-Budgeted Statistically Equivalent Signature (SES) algorithm	maxK = 2, alpha = 0.05, budget = 3 * nvars	Ridge Logistic Regression	lambda = 10.0	0.884920634920635	00:00:33.33190	false
382	Constant Removal, Standardization	LASSO Feature Selection	penalty = 0.5	Support Vector Machines	kernel = 'Polynomial Kernel', cost =	0.9256215864272129	00:03:40.220180	false

Configuration	Preprocessing	Name	Hyperparams	Name	Hyperparams	Performance (unadjusted)	Time (miliseconds)	Dropped
				(SVM) of type C-SVC	0.01, gamma = 0.01, degree = 3			
383	Constant Removal, Standardization	Test-Budgeted Statistically Equivalent Signature (SES) algorithm	maxK = 3, alpha = 0.05, budget = 3 * nvars	Classification Random Forests with Deviance splitting criterion	ntrees = 100, minimum leaf size = 3	0.889528665423806	00:01:04.64140	false
384	Constant Removal, Standardization	Test-Budgeted Statistically Equivalent Signature (SES) algorithm	maxK = 3, alpha = 0.01, budget = 3 * nvars	Support Vector Machines (SVM) of type C-SVC	kernel = 'Polynomial Kernel', cost = 0.1, gamma = 0.1, degree = 2	0.842398720915345	00:00:41.41205	false
385	Constant Removal, Standardization	Test-Budgeted Statistically Equivalent Signature (SES) algorithm	maxK = 2, alpha = 0.01, budget = 3 * nvars	Support Vector Machines (SVM) of type C-SVC	kernel = 'Polynomial Kernel', cost = 10.0, gamma = 0.01, degree = 3	0.8834767463284089	00:00:40.40981	false
386	Constant Removal, Standardization	LASSO Feature Selection	penalty = 1.0	Support Vector Machines (SVM) of type C-SVC	kernel = 'Polynomial Kernel', cost = 0.01, gamma = 1.0, degree = 2	0.9046069375353261	00:03:40.220410	false
387	Constant Removal, Standardization	Test-Budgeted Statistically Equivalent Signature (SES) algorithm	maxK = 2, alpha = 0.05, budget = 3 * nvars	Support Vector Machines (SVM) of type C-SVC	kernel = 'Radial Basis Function Kernel', cost = 1.0, gamma = 1.0	0.8849237576859059	00:00:33.33193	false
388	Constant Removal, Standardization	LASSO Feature Selection	penalty = 1.5	Classification Random Forests with Deviance splitting criterion	ntrees = 100, minimum leaf size = 3	0.9334663631338822	00:03:39.219725	false
389	Constant Removal, Standardization	Test-Budgeted Statistically Equivalent Signature (SES) algorithm	maxK = 3, alpha = 0.05, budget = 3 * nvars	Support Vector Machines (SVM) of type C-SVC	kernel = 'Linear Kernel', cost = 0.1	0.8955153967941691	00:01:04.64125	false
390	Constant Removal, Standardization	Test-Budgeted Statistically Equivalent Signature (SES) algorithm	maxK = 3, alpha = 0.05, budget = 3 * nvars	Classification Decision Tree with Deviance splitting criterion	minimum leaf size = 2, alpha = 0.05	0.7892211511137344	00:01:04.64128	false
391	Constant Removal, Standardization	LASSO Feature Selection	penalty = 1.5	Support Vector Machines (SVM) of type C-SVC	kernel = 'Radial Basis Function Kernel', cost = 0.1, gamma = 0.01	0.9228516936317448	00:03:39.219720	false
392	Constant Removal, Standardization	Test-Budgeted Statistically Equivalent Signature (SES) algorithm	maxK = 2, alpha = 0.05, budget = 3 * nvars	Classification Random Forests with Deviance splitting criterion	ntrees = 100, minimum leaf size = 4	0.8818525180417763	00:00:33.33204	false

Configuration	Preprocessing	Name	Hyperparams	Name	Hyperparams	Performance (unadjusted)	Time (miliseconds)	Dropped
393	Constant Removal, Standardization	Test-Budgeted Statistically Equivalent Signature (SES) algorithm	maxK = 3, alpha = 0.05, budget = 3 * nvars	Support Vector Machines (SVM) of type C-SVC	kernel = 'Radial Basis Function Kernel', cost = 10.0, gamma = 1.0	0.8865920950319928	00:01:04.64127	false
394	Constant Removal, Standardization	Test-Budgeted Statistically Equivalent Signature (SES) algorithm	maxK = 3, alpha = 0.05, budget = 3 * nvars	Support Vector Machines (SVM) of type C-SVC	kernel = 'Polynomial Kernel', cost = 1.0, gamma = 10.0, degree = 2	0.6303984804624191	00:01:04.64124	false
395	Constant Removal, Standardization	Test-Budgeted Statistically Equivalent Signature (SES) algorithm	maxK = 3, alpha = 0.05, budget = 3 * nvars	Support Vector Machines (SVM) of type C-SVC	kernel = 'Polynomial Kernel', cost = 10.0, gamma = 0.01, degree = 3	0.8657648120563722	00:01:04.64124	false
396	Constant Removal, Standardization	Test-Budgeted Statistically Equivalent Signature (SES) algorithm	maxK = 3, alpha = 0.01, budget = 3 * nvars	Support Vector Machines (SVM) of type C-SVC	kernel = 'Linear Kernel', cost = 1.0	0.8948229235953021	00:00:41.41206	false
397	Constant Removal, Standardization	Test-Budgeted Statistically Equivalent Signature (SES) algorithm	maxK = 2, alpha = 0.01, budget = 3 * nvars	Classification Random Forests with Deviance splitting criterion	ntrees = 500, minimum leaf size = 2	0.8868376224514332	00:00:41.41057	false
398	Constant Removal, Standardization	Test-Budgeted Statistically Equivalent Signature (SES) algorithm	maxK = 2, alpha = 0.05, budget = 3 * nvars	Support Vector Machines (SVM) of type C-SVC	kernel = 'Radial Basis Function Kernel', cost = 0.01, gamma = 10.0	0.8009791430507543	00:00:33.33193	false
399	Constant Removal, Standardization	Test-Budgeted Statistically Equivalent Signature (SES) algorithm	maxK = 2, alpha = 0.01, budget = 3 * nvars	Classification Random Forests with Deviance splitting criterion	ntrees = 100, minimum leaf size = 2	0.8880449615743733	00:00:40.40993	false
400	Constant Removal, Standardization	Test-Budgeted Statistically Equivalent Signature (SES) algorithm	maxK = 2, alpha = 0.01, budget = 3 * nvars	Support Vector Machines (SVM) of type C-SVC	kernel = 'Radial Basis Function Kernel', cost = 10.0, gamma = 0.1	0.8611532684422709	00:00:40.40981	false
401	Constant Removal, Standardization	Test-Budgeted Statistically Equivalent Signature (SES) algorithm	maxK = 2, alpha = 0.01, budget = 3 * nvars	Classification Random Forests with Deviance splitting criterion	ntrees = 500, minimum leaf size = 4	0.8850564752099279	00:00:41.41052	false
402	Constant Removal, Standardization	Test-Budgeted Statistically Equivalent Signature (SES) algorithm	maxK = 2, alpha = 0.01, budget = 3 * nvars	Support Vector Machines (SVM) of type C-SVC	kernel = 'Polynomial Kernel', cost = 10.0, gamma = 0.01, degree = 2	0.8702748657991624	00:00:40.40979	false
403	Constant Removal, Standardization	Test-Budgeted Statistically Equivalent	maxK = 3, alpha = 0.05, budget = 3 * nvars	Classification Random Forests with Deviance	ntrees = 500, minimum leaf size = 4	0.8848140705557586	00:01:04.64167	false

Configuration	Preprocessing	Name	Hyperparams	Name	Hyperparams	Performance (unadjusted)	Time (miliseconds)	Dropped
		Signature (SES) algorithm		splitting criterion				
404	Constant Removal, Standardization	LASSO Feature Selection	penalty = 1.0	Support Vector Machines (SVM) of type C-SVC	kernel = 'Polynomial Kernel', cost = 10.0, gamma = 1.0, degree = 3	0.5	00:03:40.220410	false
405	Constant Removal, Standardization	LASSO Feature Selection	penalty = 1.5	Classification Random Forests with Deviance splitting criterion	ntrees = 100, minimum leaf size = 2	0.9306016163433044	00:03:39.219726	false
406	Constant Removal, Standardization	LASSO Feature Selection	penalty = 1.5	Support Vector Machines (SVM) of type C-SVC	kernel = 'Polynomial Kernel', cost = 1.0, gamma = 0.01, degree = 2	0.9176901529842706	00:03:39.219719	false
407	Constant Removal, Standardization	Test-Budgeted Statistically Equivalent Signature (SES) algorithm	maxK = 2, alpha = 0.05, budget = 3 * nvars	Support Vector Machines (SVM) of type C-SVC	kernel = 'Polynomial Kernel', cost = 0.01, gamma = 0.1, degree = 3	0.9032469732597611	00:00:33.33190	false
408	Constant Removal, Standardization	Test-Budgeted Statistically Equivalent Signature (SES) algorithm	maxK = 2, alpha = 0.01, budget = 3 * nvars	Support Vector Machines (SVM) of type C-SVC	kernel = 'Linear Kernel', cost = 0.1	0.889970536709667	00:00:40.40980	false
409	Constant Removal, Standardization	Test-Budgeted Statistically Equivalent Signature (SES) algorithm	maxK = 3, alpha = 0.01, budget = 3 * nvars	Support Vector Machines (SVM) of type C-SVC	kernel = 'Polynomial Kernel', cost = 1.0, gamma = 10.0, degree = 2	0.6303984804624191	00:00:41.41205	false
410	Constant Removal, Standardization	LASSO Feature Selection	penalty = 0.5	Support Vector Machines (SVM) of type C-SVC	kernel = 'Linear Kernel', cost = 0.01	0.9253202395785518	00:03:40.220179	false
411	Constant Removal, Standardization	LASSO Feature Selection	penalty = 1.0	Support Vector Machines (SVM) of type C-SVC	kernel = 'Polynomial Kernel', cost = 0.1, gamma = 0.01, degree = 2	0.9129029538236701	00:03:40.220410	false
412	Constant Removal, Standardization	LASSO Feature Selection	penalty = 0.5	Support Vector Machines (SVM) of type C-SVC	kernel = 'Polynomial Kernel', cost = 0.01, gamma = 0.01, degree = 2	0.9253381954788606	00:03:40.220180	false
413	Constant Removal, Standardization	Test-Budgeted Statistically Equivalent Signature (SES) algorithm	maxK = 3, alpha = 0.01, budget = 3 * nvars	Classification Random Forests with Deviance splitting criterion	ntrees = 100, minimum leaf size = 2	0.8914323812022021	00:00:41.41218	false
414	Constant Removal, Standardization	LASSO Feature Selection	penalty = 1.5	Support Vector Machines (SVM) of type C-SVC	kernel = 'Radial Basis Function Kernel', cost = 0.01, gamma = 1.0	0.9199525964231846	00:03:39.219720	false
415	Constant Removal,	Test-Budgeted	maxK = 3, alpha = 0.05,	Support Vector	kernel = 'Radial Basis	0.8158950313681771	00:01:04.64127	false

Configuration	Preprocessing	Name	Hyperparams	Name	Hyperparams	Performance (unadjusted)	Time (miliseconds)	Dropped
	Standardization	Statistically Equivalent Signature (SES) algorithm	budget = 3 * nvars	Machines (SVM) of type C-SVC	Function Kernel', cost = 0.01, gamma = 10.0			
416	Constant Removal, Standardization	Test-Budgeted Statistically Equivalent Signature (SES) algorithm	maxK = 2, alpha = 0.01, budget = 3 * nvars	Classification Random Forests with Deviance splitting criterion	ntrees = 100, minimum leaf size = 4	0.8818525180417763	00:00:40.40992	false
417	Constant Removal, Standardization	Test-Budgeted Statistically Equivalent Signature (SES) algorithm	maxK = 3, alpha = 0.01, budget = 3 * nvars	Classification Random Forests with Deviance splitting criterion	ntrees = 500, minimum leaf size = 4	0.8905763531722611	00:00:41.41295	false
418	Constant Removal, Standardization	LASSO Feature Selection	penalty = 0.5	Ridge Logistic Regression	lambda = 1.0	0.921970683479635	00:03:40.220179	false
419	Constant Removal, Standardization	Test-Budgeted Statistically Equivalent Signature (SES) algorithm	maxK = 2, alpha = 0.05, budget = 3 * nvars	Support Vector Machines (SVM) of type C-SVC	kernel = 'Linear Kernel', cost = 10.0	0.8896153221600791	00:00:33.33196	false
420	Constant Removal, Standardization	Test-Budgeted Statistically Equivalent Signature (SES) algorithm	maxK = 2, alpha = 0.05, budget = 3 * nvars	Classification Random Forests with Deviance splitting criterion	ntrees = 500, minimum leaf size = 2	0.8805924822548862	00:00:33.33257	false
421	Constant Removal, Standardization	Test-Budgeted Statistically Equivalent Signature (SES) algorithm	maxK = 2, alpha = 0.05, budget = 3 * nvars	Ridge Logistic Regression	lambda = 0.1	0.8772366806254274	00:00:33.33190	false
422	Constant Removal, Standardization	Test-Budgeted Statistically Equivalent Signature (SES) algorithm	maxK = 3, alpha = 0.05, budget = 3 * nvars	Support Vector Machines (SVM) of type C-SVC	kernel = 'Polynomial Kernel', cost = 1.0, gamma = 10.0, degree = 3	0.808102756152628	00:01:04.64125	false
423	Constant Removal, Standardization	Test-Budgeted Statistically Equivalent Signature (SES) algorithm	maxK = 2, alpha = 0.01, budget = 3 * nvars	Support Vector Machines (SVM) of type C-SVC	kernel = 'Polynomial Kernel', cost = 0.01, gamma = 0.1, degree = 3	0.9032469732597611	00:00:40.40980	false
424	Constant Removal, Standardization	Test-Budgeted Statistically Equivalent Signature (SES) algorithm	maxK = 2, alpha = 0.05, budget = 3 * nvars	Classification Random Forests with Deviance splitting criterion	ntrees = 100, minimum leaf size = 2	0.8880449615743733	00:00:33.33225	false
425	Constant Removal, Standardization	LASSO Feature Selection	penalty = 1.5	Classification Random Forests with Deviance splitting criterion	ntrees = 500, minimum leaf size = 3	0.9321095216235882	00:03:39.219786	false

Configuration	Preprocessing	Name	Hyperparams	Name	Hyperparams	Performance (unadjusted)	Time (miliseconds)	Dropped
426	Constant Removal, Standardization	Test-Budgeted Statistically Equivalent Signature (SES) algorithm	maxK = 3, alpha = 0.01, budget = 3 * nvars	Support Vector Machines (SVM) of type C-SVC	kernel = 'Polynomial Kernel', cost = 10.0, gamma = 10.0, degree = 3	0.808102756152628	00:00:41.41205	false
427	Constant Removal, Standardization	Test-Budgeted Statistically Equivalent Signature (SES) algorithm	maxK = 3, alpha = 0.05, budget = 3 * nvars	Support Vector Machines (SVM) of type C-SVC	kernel = 'Polynomial Kernel', cost = 0.1, gamma = 0.1, degree = 3	0.8200291822414583	00:01:04.64124	false
428	Constant Removal, Standardization	LASSO Feature Selection	penalty = 1.0	Support Vector Machines (SVM) of type C-SVC	kernel = 'Radial Basis Function Kernel', cost = 0.01, gamma = 0.01	0.922478523181848	00:03:40.220412	false
429	Constant Removal, Standardization	LASSO Feature Selection	penalty = 1.5	Support Vector Machines (SVM) of type C-SVC	kernel = 'Radial Basis Function Kernel', cost = 10.0, gamma = 10.0	0.8751124195497596	00:03:39.219721	false
430	Constant Removal, Standardization	LASSO Feature Selection	penalty = 0.5	Classification Random Forests with Deviance splitting criterion	ntrees = 500, minimum leaf size = 3	0.9310438779748242	00:03:40.220253	false
431	Constant Removal, Standardization	Test-Budgeted Statistically Equivalent Signature (SES) algorithm	maxK = 2, alpha = 0.05, budget = 3 * nvars	Classification Decision Tree with Deviance splitting criterion	minimum leaf size = 4, alpha = 0.05	0.7834143690921186	00:00:33.33193	false
432	Constant Removal, Standardization	Test-Budgeted Statistically Equivalent Signature (SES) algorithm	maxK = 2, alpha = 0.05, budget = 3 * nvars	Support Vector Machines (SVM) of type C-SVC	kernel = 'Polynomial Kernel', cost = 1.0, gamma = 1.0, degree = 3	0.7255920762954012	00:00:33.33190	false
433	Constant Removal, Standardization	LASSO Feature Selection	penalty = 1.5	Classification Random Forests with Deviance splitting criterion	ntrees = 100, minimum leaf size = 2	0.9341100431253884	00:03:39.219731	false
434	Constant Removal, Standardization	Test-Budgeted Statistically Equivalent Signature (SES) algorithm	maxK = 2, alpha = 0.05, budget = 3 * nvars	Classification Random Forests with Deviance splitting criterion	ntrees = 500, minimum leaf size = 4	0.8850564752099279	00:00:33.33318	false
435	Constant Removal, Standardization	Test-Budgeted Statistically Equivalent Signature (SES) algorithm	maxK = 3, alpha = 0.01, budget = 3 * nvars	Support Vector Machines (SVM) of type C-SVC	kernel = 'Polynomial Kernel', cost = 10.0, gamma = 10.0, degree = 2	0.6303984804624191	00:00:41.41205	false
436	Constant Removal, Standardization	Test-Budgeted Statistically Equivalent Signature (SES) algorithm	maxK = 2, alpha = 0.05, budget = 3 * nvars	Support Vector Machines (SVM) of type C-SVC	kernel = 'Polynomial Kernel', cost = 1.0, gamma = 0.01, degree = 3	0.8834767463284089	00:00:33.33211	false

Configuration	Preprocessing	Name	Hyperparams	Name	Hyperparams	Performance (unadjusted)	Time (miliseconds)	Dropped
437	Constant Removal, Standardization	Test-Budgeted Statistically Equivalent Signature (SES) algorithm	maxK = 2, alpha = 0.05, budget = 3 * nvars	Support Vector Machines (SVM) of type C-SVC	kernel = 'Polynomial Kernel', cost = 10.0, gamma = 0.01, degree = 2	0.8702748657991624	00:00:33.33190	false
438	Constant Removal, Standardization	LASSO Feature Selection	penalty = 0.5	Support Vector Machines (SVM) of type C-SVC	kernel = 'Radial Basis Function Kernel', cost = 1.0, gamma = 0.01	0.9146638030909131	00:03:40.220181	false
439	Constant Removal, Standardization	LASSO Feature Selection	penalty = 1.5	Support Vector Machines (SVM) of type C-SVC	kernel = 'Polynomial Kernel', cost = 0.01, gamma = 10.0, degree = 3	0.8600345377838983	00:03:39.219719	false
440	Constant Removal, Standardization	Test-Budgeted Statistically Equivalent Signature (SES) algorithm	maxK = 3, alpha = 0.05, budget = 3 * nvars	Support Vector Machines (SVM) of type C-SVC	kernel = 'Polynomial Kernel', cost = 0.1, gamma = 0.1, degree = 2	0.842398720915345	00:01:04.64124	false
441	Constant Removal, Standardization	Test-Budgeted Statistically Equivalent Signature (SES) algorithm	maxK = 3, alpha = 0.05, budget = 3 * nvars	Classification Random Forests with Deviance splitting criterion	ntrees = 500, minimum leaf size = 2	0.8897023692420112	00:01:04.64205	false
442	Constant Removal, Standardization	Test-Budgeted Statistically Equivalent Signature (SES) algorithm	maxK = 3, alpha = 0.01, budget = 3 * nvars	Support Vector Machines (SVM) of type C-SVC	kernel = 'Radial Basis Function Kernel', cost = 10.0, gamma = 1.0	0.8865920950319928	00:00:41.41207	false
443	Constant Removal, Standardization	Test-Budgeted Statistically Equivalent Signature (SES) algorithm	maxK = 3, alpha = 0.01, budget = 3 * nvars	Support Vector Machines (SVM) of type C-SVC	kernel = 'Polynomial Kernel', cost = 10.0, gamma = 0.1, degree = 2	0.8263039887080807	00:00:41.41205	false
444	Constant Removal, Standardization	Test-Budgeted Statistically Equivalent Signature (SES) algorithm	maxK = 2, alpha = 0.01, budget = 3 * nvars	Support Vector Machines (SVM) of type C-SVC	kernel = 'Polynomial Kernel', cost = 0.1, gamma = 0.1, degree = 2	0.8272181782411963	00:00:40.40981	false
445	Constant Removal, Standardization	LASSO Feature Selection	penalty = 1.5	Support Vector Machines (SVM) of type C-SVC	kernel = 'Polynomial Kernel', cost = 0.01, gamma = 0.1, degree = 2	0.9252843277779339	00:03:39.219719	false
446	Constant Removal, Standardization	LASSO Feature Selection	penalty = 0.5	Classification Random Forests with Deviance splitting criterion	ntrees = 100, minimum leaf size = 4	0.9307144262387228	00:03:40.220186	false
447	Constant Removal, Standardization	LASSO Feature Selection	penalty = 1.0	Support Vector Machines (SVM) of type C-SVC	kernel = 'Polynomial Kernel', cost = 10.0, gamma = 1.0, degree = 2	0.5	00:03:40.220410	false
448	Constant Removal, Standardization	Test-Budgeted Statistically	maxK = 2, alpha = 0.05, budget = 3 *	Classification Decision Tree with Deviance	minimum leaf size = 2, alpha = 0.05	0.7743702553485162	00:00:33.33194	false

Configuration	Preprocessing	Name	Hyperparams	Name	Hyperparams	Performance (unadjusted)	Time (miliseconds)	Dropped
		Equivalent Signature (SES) algorithm	nvars	splitting criterion				
449	Constant Removal, Standardization	Test-Budgeted Statistically Equivalent Signature (SES) algorithm	maxK = 3, alpha = 0.01, budget = 3 * nvars	Support Vector Machines (SVM) of type C-SVC	kernel = 'Radial Basis Function Kernel', cost = 0.1, gamma = 1.0	0.8742224314474953	00:00:41.41206	false
450	Constant Removal, Standardization	LASSO Feature Selection	penalty = 1.0	Classification Random Forests with Deviance splitting criterion	ntrees = 500, minimum leaf size = 2	0.9332309847015732	00:03:40.220480	false
451	Constant Removal, Standardization	LASSO Feature Selection	penalty = 1.0	Support Vector Machines (SVM) of type C-SVC	kernel = 'Polynomial Kernel', cost = 10.0, gamma = 10.0, degree = 3	0.7986382401344039	00:03:40.220410	false
452	Constant Removal, Standardization	Test-Budgeted Statistically Equivalent Signature (SES) algorithm	maxK = 3, alpha = 0.05, budget = 3 * nvars	Classification Random Forests with Deviance splitting criterion	ntrees = 500, minimum leaf size = 3	0.8890407333501961	00:01:04.64200	false
453	Constant Removal, Standardization	LASSO Feature Selection	penalty = 1.0	Support Vector Machines (SVM) of type C-SVC	kernel = 'Polynomial Kernel', cost = 0.1, gamma = 10.0, degree = 2	0.5637766254773928	00:03:40.220410	false
454	Constant Removal, Standardization	LASSO Feature Selection	penalty = 1.5	Support Vector Machines (SVM) of type C-SVC	kernel = 'Radial Basis Function Kernel', cost = 1.0, gamma = 0.01	0.9199920213347325	00:03:39.219720	false
455	Constant Removal, Standardization	LASSO Feature Selection	penalty = 0.5	Support Vector Machines (SVM) of type C-SVC	kernel = 'Radial Basis Function Kernel', cost = 10.0, gamma = 10.0	0.8708498449547043	00:03:40.220183	false
456	Constant Removal, Standardization	LASSO Feature Selection	penalty = 1.5	Support Vector Machines (SVM) of type C-SVC	kernel = 'Polynomial Kernel', cost = 0.1, gamma = 0.1, degree = 3	0.6475533914792228	00:03:39.219719	false
457	Constant Removal, Standardization	LASSO Feature Selection	penalty = 1.5	Classification Random Forests with Deviance splitting criterion	ntrees = 100, minimum leaf size = 2	0.9341100431253884	00:03:39.219731	false
458	Constant Removal, Standardization	LASSO Feature Selection	penalty = 1.5	Support Vector Machines (SVM) of type C-SVC	kernel = 'Polynomial Kernel', cost = 0.01, gamma = 1.0, degree = 3	0.8823958791989481	00:03:39.219719	false
459	Constant Removal, Standardization	LASSO Feature Selection	penalty = 1.5	Support Vector Machines (SVM) of type C-SVC	kernel = 'Polynomial Kernel', cost = 1.0, gamma = 10.0, degree = 3	0.8004143128823435	00:03:39.219719	false

Configuration	Preprocessing	Name	Hyperparams	Name	Hyperparams	Performance (unadjusted)	Time (miliseconds)	Dropped
460	Constant Removal, Standardization	LASSO Feature Selection	penalty = 1.0	Classification Random Forests with Deviance splitting criterion	ntrees = 500, minimum leaf size = 4	0.9328937260522939	00:03:40.220476	false
461	Constant Removal, Standardization	Test-Budgeted Statistically Equivalent Signature (SES) algorithm	maxK = 2, alpha = 0.01, budget = 3 * nvars	Classification Random Forests with Deviance splitting criterion	ntrees = 500, minimum leaf size = 3	0.8864234657073533	00:00:41.41050	false
462	Constant Removal, Standardization	LASSO Feature Selection	penalty = 0.5	Support Vector Machines (SVM) of type C-SVC	kernel = 'Polynomial Kernel', cost = 10.0, gamma = 0.01, degree = 3	0.9176979598974483	00:03:40.220179	false
463	Constant Removal, Standardization	Test-Budgeted Statistically Equivalent Signature (SES) algorithm	maxK = 2, alpha = 0.05, budget = 3 * nvars	Classification Random Forests with Deviance splitting criterion	ntrees = 500, minimum leaf size = 2	0.8868376224514332	00:00:33.33301	false
464	Constant Removal, Standardization	Test-Budgeted Statistically Equivalent Signature (SES) algorithm	maxK = 3, alpha = 0.01, budget = 3 * nvars	Support Vector Machines (SVM) of type C-SVC	kernel = 'Polynomial Kernel', cost = 0.01, gamma = 10.0, degree = 2	0.6660860274990709	00:00:41.41205	false
465	Constant Removal, Standardization	LASSO Feature Selection	penalty = 1.0	Support Vector Machines (SVM) of type C-SVC	kernel = 'Linear Kernel', cost = 0.1	0.9253202395785518	00:03:40.220410	false
466	Constant Removal, Standardization	LASSO Feature Selection	penalty = 1.0	Classification Random Forests with Deviance splitting criterion	ntrees = 500, minimum leaf size = 4	0.9320045186413474	00:03:40.220448	false
467	Constant Removal, Standardization	Test-Budgeted Statistically Equivalent Signature (SES) algorithm	maxK = 3, alpha = 0.01, budget = 3 * nvars	Classification Decision Tree with Deviance splitting criterion	minimum leaf size = 3, alpha = 0.05	0.8026699252722271	00:00:41.41208	false
468	Constant Removal, Standardization	Test-Budgeted Statistically Equivalent Signature (SES) algorithm	maxK = 2, alpha = 0.05, budget = 3 * nvars	Classification Decision Tree with Deviance splitting criterion	minimum leaf size = 3, alpha = 0.05	0.7725496831954634	00:00:33.33194	false
469	Constant Removal, Standardization	Test-Budgeted Statistically Equivalent Signature (SES) algorithm	maxK = 3, alpha = 0.05, budget = 3 * nvars	Classification Decision Tree with Deviance splitting criterion	minimum leaf size = 2, alpha = 0.01	0.8003145795664977	00:01:04.64128	false
470	Constant Removal, Standardization	Test-Budgeted Statistically Equivalent Signature (SES) algorithm	maxK = 3, alpha = 0.05, budget = 3 * nvars	Support Vector Machines (SVM) of type C-SVC	kernel = 'Polynomial Kernel', cost = 0.01, gamma = 0.1, degree = 3	0.8989059391872692	00:01:04.64125	false

Configuration	Preprocessing	Name	Hyperparams	Name	Hyperparams	Performance (unadjusted)	Time (miliseconds)	Dropped
471	Constant Removal, Standardization	LASSO Feature Selection	penalty = 1.5	Classification Decision Tree with Deviance splitting criterion	minimum leaf size = 2, alpha = 0.01	0.8880076835639494	00:03:39.219722	false
472	Constant Removal, Standardization	LASSO Feature Selection	penalty = 0.5	Ridge Logistic Regression	lambda = 0.1	0.9216513807306647	00:03:40.220179	false
473	Constant Removal, Standardization	Test-Budgeted Statistically Equivalent Signature (SES) algorithm	maxK = 3, alpha = 0.01, budget = 3 * nvars	Support Vector Machines (SVM) of type C-SVC	kernel = 'Radial Basis Function Kernel', cost = 1.0, gamma = 0.01	0.7477242848086837	00:00:41.41206	false
474	Constant Removal, Standardization	Test-Budgeted Statistically Equivalent Signature (SES) algorithm	maxK = 3, alpha = 0.01, budget = 3 * nvars	Classification Random Forests with Deviance splitting criterion	ntrees = 100, minimum leaf size = 2	0.8914323812022021	00:00:41.41218	false
475	Constant Removal, Standardization	LASSO Feature Selection	penalty = 1.0	Classification Random Forests with Deviance splitting criterion	ntrees = 500, minimum leaf size = 3	0.9314857492606854	00:03:40.220456	false
476	Constant Removal, Standardization	LASSO Feature Selection	penalty = 1.5	Support Vector Machines (SVM) of type C-SVC	kernel = 'Polynomial Kernel', cost = 0.01, gamma = 0.1, degree = 3	0.9259768009768008	00:03:39.219719	false
477	Constant Removal, Standardization	LASSO Feature Selection	penalty = 1.0	Classification Random Forests with Deviance splitting criterion	ntrees = 500, minimum leaf size = 2	0.9332309847015732	00:03:40.220481	false
478	Constant Removal, Standardization	Test-Budgeted Statistically Equivalent Signature (SES) algorithm	maxK = 3, alpha = 0.05, budget = 3 * nvars	Support Vector Machines (SVM) of type C-SVC	kernel = 'Polynomial Kernel', cost = 0.1, gamma = 1.0, degree = 2	0.7889127780432128	00:01:04.64125	false
479	Constant Removal, Standardization	LASSO Feature Selection	penalty = 1.5	Support Vector Machines (SVM) of type C-SVC	kernel = 'Polynomial Kernel', cost = 0.1, gamma = 1.0, degree = 2	0.6649448519653124	00:03:39.219719	false
480	Constant Removal, Standardization	Test-Budgeted Statistically Equivalent Signature (SES) algorithm	maxK = 3, alpha = 0.01, budget = 3 * nvars	Classification Decision Tree with Deviance splitting criterion	minimum leaf size = 4, alpha = 0.05	0.8178631541802899	00:00:41.41207	false
481	Constant Removal, Standardization	Test-Budgeted Statistically Equivalent Signature (SES) algorithm	maxK = 2, alpha = 0.05, budget = 3 * nvars	Classification Random Forests with Deviance splitting criterion	ntrees = 100, minimum leaf size = 3	0.8794159804389983	00:00:33.33204	false
482	Constant Removal, Standardization	Test-Budgeted Statistically Equivalent Signature (SES) algorithm	maxK = 2, alpha = 0.01, budget = 3 * nvars	Support Vector Machines (SVM) of type C-SVC	kernel = 'Radial Basis Function Kernel', cost = 0.1, gamma = 10.0	0.8019858445050262	00:00:40.40984	false

Configuration	Preprocessing	Name	Hyperparams	Name	Hyperparams	Performance (unadjusted)	Time (miliseconds)	Dropped
483	Constant Removal, Standardization	LASSO Feature Selection	penalty = 1.5	Classification Random Forests with Deviance splitting criterion	ntrees = 100, minimum leaf size = 3	0.9408759044308916	00:03:39.219731	false
484	Constant Removal, Standardization	Test-Budgeted Statistically Equivalent Signature (SES) algorithm	maxK = 2, alpha = 0.01, budget = 3 * nvars	Support Vector Machines (SVM) of type C-SVC	kernel = 'Polynomial Kernel', cost = 1.0, gamma = 1.0, degree = 2	0.7271331609566903	00:00:40.40980	false
485	Constant Removal, Standardization	LASSO Feature Selection	penalty = 1.0	Support Vector Machines (SVM) of type C-SVC	kernel = 'Polynomial Kernel', cost = 1.0, gamma = 0.1, degree = 2	0.7087129444866018	00:03:40.220410	false
486	Constant Removal, Standardization	Test-Budgeted Statistically Equivalent Signature (SES) algorithm	maxK = 3, alpha = 0.05, budget = 3 * nvars	Support Vector Machines (SVM) of type C-SVC	kernel = 'Radial Basis Function Kernel', cost = 0.1, gamma = 0.01	0.8493289177432398	00:01:04.64127	false
487	Constant Removal, Standardization	LASSO Feature Selection	penalty = 1.5	Classification Decision Tree with Deviance splitting criterion	minimum leaf size = 4, alpha = 0.05	0.8646757476680752	00:03:39.219721	false
488	Constant Removal, Standardization	LASSO Feature Selection	penalty = 0.5	Classification Random Forests with Deviance splitting criterion	ntrees = 500, minimum leaf size = 2	0.9325334370091403	00:03:40.220220	false
489	Constant Removal, Standardization	Test-Budgeted Statistically Equivalent Signature (SES) algorithm	maxK = 2, alpha = 0.01, budget = 3 * nvars	Classification Random Forests with Deviance splitting criterion	ntrees = 100, minimum leaf size = 2	0.8880449615743733	00:00:40.40992	false
490	Constant Removal, Standardization	Test-Budgeted Statistically Equivalent Signature (SES) algorithm	maxK = 3, alpha = 0.05, budget = 3 * nvars	Support Vector Machines (SVM) of type C-SVC	kernel = 'Polynomial Kernel', cost = 0.01, gamma = 1.0, degree = 3	0.861526438892168	00:01:04.64124	false
491	Constant Removal, Standardization	LASSO Feature Selection	penalty = 0.5	Support Vector Machines (SVM) of type C-SVC	kernel = 'Polynomial Kernel', cost = 0.1, gamma = 0.01, degree = 3	0.893186984938903	00:03:40.220180	false
492	Constant Removal, Standardization	Test-Budgeted Statistically Equivalent Signature (SES) algorithm	maxK = 3, alpha = 0.01, budget = 3 * nvars	Support Vector Machines (SVM) of type C-SVC	kernel = 'Polynomial Kernel', cost = 0.1, gamma = 1.0, degree = 2	0.7889127780432128	00:00:41.41205	false
493	Constant Removal, Standardization	LASSO Feature Selection	penalty = 1.0	Support Vector Machines (SVM) of type C-SVC	kernel = 'Polynomial Kernel', cost = 1.0, gamma = 0.01, degree = 3	0.9176979598974483	00:03:40.220410	false
494	Constant Removal, Standardization	LASSO Feature Selection	penalty = 0.5	Support Vector Machines (SVM) of type	kernel = 'Polynomial Kernel', cost = 0.1, gamma =	0.6952345040580334	00:03:40.220179	false

Configuration	Preprocessing	Name	Hyperparams	Name	Hyperparams	Performance (unadjusted)	Time (miliseconds)	Dropped
				C-SVC	1.0, degree = 3			
495	Constant Removal, Standardization	Test-Budgeted Statistically Equivalent Signature (SES) algorithm	maxK = 2, alpha = 0.01, budget = 3 * nvars	Support Vector Machines (SVM) of type C-SVC	kernel = 'Radial Basis Function Kernel', cost = 1.0, gamma = 10.0	0.8009561126568799	00:00:40.40982	false
496	Constant Removal, Standardization	LASSO Feature Selection	penalty = 1.5	Classification Decision Tree with Deviance splitting criterion	minimum leaf size = 3, alpha = 0.05	0.8782617283256671	00:03:39.219722	false
497	Constant Removal, Standardization	Test-Budgeted Statistically Equivalent Signature (SES) algorithm	maxK = 2, alpha = 0.01, budget = 3 * nvars	Classification Decision Tree with Deviance splitting criterion	minimum leaf size = 4, alpha = 0.05	0.7834143690921186	00:00:40.40983	false
498	Constant Removal, Standardization	Test-Budgeted Statistically Equivalent Signature (SES) algorithm	maxK = 3, alpha = 0.01, budget = 3 * nvars	Support Vector Machines (SVM) of type C-SVC	kernel = 'Polynomial Kernel', cost = 1.0, gamma = 1.0, degree = 2	0.7585610609907285	00:00:41.41205	false
499	Constant Removal, Standardization	LASSO Feature Selection	penalty = 0.5	Classification Random Forests with Deviance splitting criterion	ntrees = 100, minimum leaf size = 4	0.9359239794022403	00:03:40.220192	false
500	Constant Removal, Standardization	Test-Budgeted Statistically Equivalent Signature (SES) algorithm	maxK = 3, alpha = 0.05, budget = 3 * nvars	Support Vector Machines (SVM) of type C-SVC	kernel = 'Linear Kernel', cost = 1.0	0.8948229235953021	00:01:04.64126	false
501	Constant Removal, Standardization	LASSO Feature Selection	penalty = 1.0	Support Vector Machines (SVM) of type C-SVC	kernel = 'Radial Basis Function Kernel', cost = 10.0, gamma = 0.01	0.9146638030909131	00:03:40.220412	false
502	Constant Removal, Standardization	LASSO Feature Selection	penalty = 1.5	Support Vector Machines (SVM) of type C-SVC	kernel = 'Polynomial Kernel', cost = 1.0, gamma = 1.0, degree = 2	0.5	00:03:39.219719	false
503	Constant Removal, Standardization	Test-Budgeted Statistically Equivalent Signature (SES) algorithm	maxK = 2, alpha = 0.01, budget = 3 * nvars	Classification Random Forests with Deviance splitting criterion	ntrees = 100, minimum leaf size = 3	0.8794159804389983	00:00:40.40992	false
504	Constant Removal, Standardization	LASSO Feature Selection	penalty = 1.0	Support Vector Machines (SVM) of type C-SVC	kernel = 'Radial Basis Function Kernel', cost = 0.1, gamma = 0.1	0.92506105006105	00:03:40.220412	false
505	Constant Removal, Standardization	Test-Budgeted Statistically Equivalent Signature (SES) algorithm	maxK = 3, alpha = 0.01, budget = 3 * nvars	Support Vector Machines (SVM) of type C-SVC	kernel = 'Radial Basis Function Kernel', cost = 0.1, gamma = 10.0	0.8160367268423534	00:00:41.41207	false
506	Constant Removal,	LASSO Feature	penalty = 1.5	Support Vector	kernel = 'Radial Basis	0.8731400029353994	00:03:39.219721	false

Configuration	Preprocessing	Name	Hyperparams	Name	Hyperparams	Performance (unadjusted)	Time (miliseconds)	Dropped
	Standardization	Selection		Machines (SVM) of type C-SVC	Function Kernel', cost = 0.1, gamma = 10.0			
507	Constant Removal, Standardization	Test-Budgeted Statistically Equivalent Signature (SES) algorithm	maxK = 2, alpha = 0.01, budget = 3 * nvars	Support Vector Machines (SVM) of type C-SVC	kernel = 'Radial Basis Function Kernel', cost = 0.1, gamma = 1.0	0.8744812306193381	00:00:40.40980	false
508	Constant Removal, Standardization	LASSO Feature Selection	penalty = 0.5	Classification Random Forests with Deviance splitting criterion	ntrees = 100, minimum leaf size = 4	0.9359239794022403	00:03:40.220193	false
509	Constant Removal, Standardization	Test-Budgeted Statistically Equivalent Signature (SES) algorithm	maxK = 2, alpha = 0.05, budget = 3 * nvars	Support Vector Machines (SVM) of type C-SVC	kernel = 'Radial Basis Function Kernel', cost = 1.0, gamma = 0.01	0.7631484031739786	00:00:33.33192	false
510	Constant Removal, Standardization	Test-Budgeted Statistically Equivalent Signature (SES) algorithm	maxK = 2, alpha = 0.05, budget = 3 * nvars	Classification Random Forests with Deviance splitting criterion	ntrees = 500, minimum leaf size = 3	0.8864234657073533	00:00:33.33300	false
511	Constant Removal, Standardization	Test-Budgeted Statistically Equivalent Signature (SES) algorithm	maxK = 2, alpha = 0.01, budget = 3 * nvars	Ridge Logistic Regression	lambda = 0.1	0.8772366806254274	00:00:40.40979	false
512	Constant Removal, Standardization	LASSO Feature Selection	penalty = 0.5	Support Vector Machines (SVM) of type C-SVC	kernel = 'Radial Basis Function Kernel', cost = 1.0, gamma = 10.0	0.8695160338382846	00:03:40.220182	false
513	Constant Removal, Standardization	LASSO Feature Selection	penalty = 1.5	Classification Random Forests with Deviance splitting criterion	ntrees = 100, minimum leaf size = 2	0.9341100431253884	00:03:39.219731	false
514	Constant Removal, Standardization	Test-Budgeted Statistically Equivalent Signature (SES) algorithm	maxK = 2, alpha = 0.05, budget = 3 * nvars	Classification Random Forests with Deviance splitting criterion	ntrees = 100, minimum leaf size = 2	0.8880449615743733	00:00:33.33204	false
515	Constant Removal, Standardization	LASSO Feature Selection	penalty = 1.0	Support Vector Machines (SVM) of type C-SVC	kernel = 'Radial Basis Function Kernel', cost = 1.0, gamma = 0.1	0.9212192680862755	00:03:40.220412	false
516	Constant Removal, Standardization	Test-Budgeted Statistically Equivalent Signature (SES) algorithm	maxK = 2, alpha = 0.05, budget = 3 * nvars	Classification Random Forests with Deviance splitting criterion	ntrees = 100, minimum leaf size = 3	0.8794159804389983	00:00:33.33206	false

Configuration	Preprocessing	Name	Hyperparams	Name	Hyperparams	Performance (unadjusted)	Time (miliseconds)	Dropped
517	Constant Removal, Standardization	Test-Budgeted Statistically Equivalent Signature (SES) algorithm	maxK = 3, alpha = 0.01, budget = 3 * nvars	Support Vector Machines (SVM) of type C-SVC	kernel = 'Polynomial Kernel', cost = 0.01, gamma = 0.1, degree = 3	0.8989059391872692	00:00:41.41205	false
518	Constant Removal, Standardization	Test-Budgeted Statistically Equivalent Signature (SES) algorithm	maxK = 3, alpha = 0.01, budget = 3 * nvars	Classification Decision Tree with Deviance splitting criterion	minimum leaf size = 2, alpha = 0.01	0.8003145795664977	00:00:41.41208	false
519	Constant Removal, Standardization	Test-Budgeted Statistically Equivalent Signature (SES) algorithm	maxK = 2, alpha = 0.05, budget = 3 * nvars	Support Vector Machines (SVM) of type C-SVC	kernel = 'Polynomial Kernel', cost = 1.0, gamma = 10.0, degree = 3	0.778636538227331	00:00:33.33191	false
520	Constant Removal, Standardization	Test-Budgeted Statistically Equivalent Signature (SES) algorithm	maxK = 2, alpha = 0.01, budget = 3 * nvars	Classification Random Forests with Deviance splitting criterion	ntrees = 500, minimum leaf size = 2	0.8805924822548862	00:00:41.41022	false
521	Constant Removal, Standardization	Test-Budgeted Statistically Equivalent Signature (SES) algorithm	maxK = 2, alpha = 0.01, budget = 3 * nvars	Support Vector Machines (SVM) of type C-SVC	kernel = 'Radial Basis Function Kernel', cost = 1.0, gamma = 1.0	0.8849237576859059	00:00:40.40983	false
522	Constant Removal, Standardization	Test-Budgeted Statistically Equivalent Signature (SES) algorithm	maxK = 3, alpha = 0.01, budget = 3 * nvars	Support Vector Machines (SVM) of type C-SVC	kernel = 'Polynomial Kernel', cost = 0.1, gamma = 0.1, degree = 3	0.8200291822414583	00:00:41.41205	false
523	Constant Removal, Standardization	Test-Budgeted Statistically Equivalent Signature (SES) algorithm	maxK = 3, alpha = 0.05, budget = 3 * nvars	Support Vector Machines (SVM) of type C-SVC	kernel = 'Polynomial Kernel', cost = 0.01, gamma = 0.01, degree = 3	0.8996522800870627	00:01:04.64125	false
524	Constant Removal, Standardization	Test-Budgeted Statistically Equivalent Signature (SES) algorithm	maxK = 2, alpha = 0.01, budget = 3 * nvars	Support Vector Machines (SVM) of type C-SVC	kernel = 'Polynomial Kernel', cost = 10.0, gamma = 10.0, degree = 3	0.778636538227331	00:00:40.40982	false
525	Constant Removal, Standardization	LASSO Feature Selection	penalty = 1.5	Support Vector Machines (SVM) of type C-SVC	kernel = 'Radial Basis Function Kernel', cost = 0.1, gamma = 1.0	0.9203035171705249	00:03:39.219721	false
526	Constant Removal, Standardization	LASSO Feature Selection	penalty = 1.5	Classification Random Forests with Deviance splitting criterion	ntrees = 500, minimum leaf size = 3	0.9321095216235882	00:03:39.219785	false
527	Constant Removal, Standardization	LASSO Feature Selection	penalty = 1.5	Classification Random Forests with Deviance splitting criterion	ntrees = 100, minimum leaf size = 4	0.9384104812493559	00:03:39.219731	false

Configuration	Preprocessing	Name	Hyperparams	Name	Hyperparams	Performance (unadjusted)	Time (miliseconds)	Dropped
528	Constant Removal, Standardization	Test-Budgeted Statistically Equivalent Signature (SES) algorithm	maxK = 2, alpha = 0.01, budget = 3 * nvars	Classification Random Forests with Deviance splitting criterion	ntrees = 500, minimum leaf size = 2	0.8868376224514332	00:00:41.41124	false
529	Constant Removal, Standardization	Test-Budgeted Statistically Equivalent Signature (SES) algorithm	maxK = 2, alpha = 0.01, budget = 3 * nvars	Support Vector Machines (SVM) of type C-SVC	kernel = 'Radial Basis Function Kernel', cost = 1.0, gamma = 0.01	0.7631484031739786	00:00:40.40982	false
530	Constant Removal, Standardization	LASSO Feature Selection	penalty = 1.5	Support Vector Machines (SVM) of type C-SVC	kernel = 'Linear Kernel', cost = 1.0	0.9249650250289635	00:03:39.219719	false
531	Constant Removal, Standardization	Test-Budgeted Statistically Equivalent Signature (SES) algorithm	maxK = 2, alpha = 0.01, budget = 3 * nvars	Support Vector Machines (SVM) of type C-SVC	kernel = 'Polynomial Kernel', cost = 10.0, gamma = 1.0, degree = 2	0.7271331609566903	00:00:40.40982	false
532	Constant Removal, Standardization	LASSO Feature Selection	penalty = 1.5	Support Vector Machines (SVM) of type C-SVC	kernel = 'Polynomial Kernel', cost = 0.01, gamma = 0.01, degree = 3	0.9256215864272129	00:03:39.219719	false
533	Constant Removal, Standardization	Test-Budgeted Statistically Equivalent Signature (SES) algorithm	maxK = 2, alpha = 0.01, budget = 3 * nvars	Support Vector Machines (SVM) of type C-SVC	kernel = 'Polynomial Kernel', cost = 1.0, gamma = 0.1, degree = 2	0.7732376674192531	00:00:40.40982	false
534	Constant Removal, Standardization	LASSO Feature Selection	penalty = 1.5	Support Vector Machines (SVM) of type C-SVC	kernel = 'Polynomial Kernel', cost = 0.01, gamma = 1.0, degree = 2	0.9030084720621804	00:03:39.219719	false
535	Constant Removal, Standardization	LASSO Feature Selection	penalty = 0.5	Support Vector Machines (SVM) of type C-SVC	kernel = 'Radial Basis Function Kernel', cost = 0.01, gamma = 10.0	0.868540169691065	00:03:40.220182	false
536	Constant Removal, Standardization	Test-Budgeted Statistically Equivalent Signature (SES) algorithm	maxK = 2, alpha = 0.05, budget = 3 * nvars	Classification Random Forests with Deviance splitting criterion	ntrees = 100, minimum leaf size = 3	0.8794159804389983	00:00:33.33204	false
537	Constant Removal, Standardization	Test-Budgeted Statistically Equivalent Signature (SES) algorithm	maxK = 3, alpha = 0.05, budget = 3 * nvars	Support Vector Machines (SVM) of type C-SVC	kernel = 'Radial Basis Function Kernel', cost = 0.1, gamma = 1.0	0.8742224314474953	00:01:04.64126	false
538	Constant Removal, Standardization	Test-Budgeted Statistically Equivalent Signature (SES) algorithm	maxK = 3, alpha = 0.05, budget = 3 * nvars	Classification Random Forests with Deviance splitting criterion	ntrees = 100, minimum leaf size = 3	0.889528665423806	00:01:04.64138	false
539	Constant Removal,	LASSO Feature	penalty = 1.5	Classification Random	ntrees = 100, minimum leaf	0.9408759044308916	00:03:39.219732	false

Configuration	Preprocessing	Name	Hyperparams	Name	Hyperparams	Performance (unadjusted)	Time (miliseconds)	Dropped
	Standardization	Selection		Forests with Deviance splitting criterion	size = 3			
540	Constant Removal, Standardization	LASSO Feature Selection	penalty = 1.0	Support Vector Machines (SVM) of type C-SVC	kernel = 'Polynomial Kernel', cost = 0.1, gamma = 0.1, degree = 2	0.7169107888417352	00:03:40.220410	false
541	Constant Removal, Standardization	LASSO Feature Selection	penalty = 1.5	Support Vector Machines (SVM) of type C-SVC	kernel = 'Radial Basis Function Kernel', cost = 1.0, gamma = 1.0	0.9156127333876695	00:03:39.219721	false
542	IdentityFactory	NoSelector	-	Trivial model	-	0.5	00:00:00.000	false
543	Constant Removal, Standardization	Test-Budgeted Statistically Equivalent Signature (SES) algorithm	maxK = 3, alpha = 0.01, budget = 3 * nvars	Classification Random Forests with Deviance splitting criterion	ntrees = 100, minimum leaf size = 3	0.889528665423806	00:00:41.41218	false
544	Constant Removal, Standardization	LASSO Feature Selection	penalty = 1.0	Support Vector Machines (SVM) of type C-SVC	kernel = 'Radial Basis Function Kernel', cost = 0.1, gamma = 0.01	0.9228516936317448	00:03:40.220412	false
545	Constant Removal, Standardization	Test-Budgeted Statistically Equivalent Signature (SES) algorithm	maxK = 3, alpha = 0.05, budget = 3 * nvars	Support Vector Machines (SVM) of type C-SVC	kernel = 'Radial Basis Function Kernel', cost = 1.0, gamma = 0.1	0.862127571206855	00:01:04.64127	false
546	Constant Removal, Standardization	Test-Budgeted Statistically Equivalent Signature (SES) algorithm	maxK = 3, alpha = 0.01, budget = 3 * nvars	Support Vector Machines (SVM) of type C-SVC	kernel = 'Radial Basis Function Kernel', cost = 1.0, gamma = 1.0	0.8865920950319928	00:00:41.41207	false
547	Constant Removal, Standardization	LASSO Feature Selection	penalty = 0.5	Classification Random Forests with Deviance splitting criterion	ntrees = 100, minimum leaf size = 2	0.9337548285758005	00:03:40.220194	false
548	Constant Removal, Standardization	Test-Budgeted Statistically Equivalent Signature (SES) algorithm	maxK = 2, alpha = 0.05, budget = 3 * nvars	Support Vector Machines (SVM) of type C-SVC	kernel = 'Radial Basis Function Kernel', cost = 0.1, gamma = 0.01	0.8425298770567313	00:00:33.33192	false
549	Constant Removal, Standardization	Test-Budgeted Statistically Equivalent Signature (SES) algorithm	maxK = 3, alpha = 0.01, budget = 3 * nvars	Support Vector Machines (SVM) of type C-SVC	kernel = 'Polynomial Kernel', cost = 0.01, gamma = 0.01, degree = 3	0.8996522800870627	00:00:41.41205	false
550	Constant Removal, Standardization	Test-Budgeted Statistically Equivalent Signature (SES) algorithm	maxK = 2, alpha = 0.01, budget = 3 * nvars	Support Vector Machines (SVM) of type C-SVC	kernel = 'Polynomial Kernel', cost = 0.1, gamma = 1.0, degree = 2	0.7913824950269963	00:00:40.40981	false

Configuration	Preprocessing	Name	Hyperparams	Name	Hyperparams	Performance (unadjusted)	Time (miliseconds)	Dropped
551	Constant Removal, Standardization	LASSO Feature Selection	penalty = 1.0	Support Vector Machines (SVM) of type C-SVC	kernel = 'Polynomial Kernel', cost = 1.0, gamma = 10.0, degree = 2	0.5255422681893269	00:03:40.220410	false
552	Constant Removal, Standardization	LASSO Feature Selection	penalty = 1.0	Ridge Logistic Regression	lambda = 10.0	0.9245738986787582	00:03:40.220410	false
553	Constant Removal, Standardization	Test-Budgeted Statistically Equivalent Signature (SES) algorithm	maxK = 3, alpha = 0.01, budget = 3 * nvars	Classification Random Forests with Deviance splitting criterion	ntrees = 100, minimum leaf size = 4	0.8857520711740662	00:00:41.41218	false
554	Constant Removal, Standardization	Test-Budgeted Statistically Equivalent Signature (SES) algorithm	maxK = 3, alpha = 0.05, budget = 3 * nvars	Classification Decision Tree with Deviance splitting criterion	minimum leaf size = 3, alpha = 0.05	0.8026699252722271	00:01:04.64128	false
555	Constant Removal, Standardization	Test-Budgeted Statistically Equivalent Signature (SES) algorithm	maxK = 3, alpha = 0.01, budget = 3 * nvars	Support Vector Machines (SVM) of type C-SVC	kernel = 'Radial Basis Function Kernel', cost = 1.0, gamma = 10.0	0.8150069949942071	00:00:41.41208	false
556	Constant Removal, Standardization	Test-Budgeted Statistically Equivalent Signature (SES) algorithm	maxK = 3, alpha = 0.05, budget = 3 * nvars	Support Vector Machines (SVM) of type C-SVC	kernel = 'Polynomial Kernel', cost = 0.01, gamma = 1.0, degree = 2	0.8786138201099837	00:01:04.64125	false
557	Constant Removal, Standardization	Test-Budgeted Statistically Equivalent Signature (SES) algorithm	maxK = 3, alpha = 0.05, budget = 3 * nvars	Support Vector Machines (SVM) of type C-SVC	kernel = 'Radial Basis Function Kernel', cost = 0.1, gamma = 10.0	0.8160367268423534	00:01:04.64127	false
558	Constant Removal, Standardization	Test-Budgeted Statistically Equivalent Signature (SES) algorithm	maxK = 3, alpha = 0.01, budget = 3 * nvars	Classification Random Forests with Deviance splitting criterion	ntrees = 500, minimum leaf size = 3	0.8890407333501961	00:00:41.41296	false
559	Constant Removal, Standardization	LASSO Feature Selection	penalty = 1.5	Support Vector Machines (SVM) of type C-SVC	kernel = 'Polynomial Kernel', cost = 10.0, gamma = 1.0, degree = 3	0.5	00:03:39.219719	false
560	Constant Removal, Standardization	Test-Budgeted Statistically Equivalent Signature (SES) algorithm	maxK = 2, alpha = 0.05, budget = 3 * nvars	Support Vector Machines (SVM) of type C-SVC	kernel = 'Polynomial Kernel', cost = 10.0, gamma = 1.0, degree = 3	0.7255920762954012	00:00:33.33190	false
561	Constant Removal, Standardization	Test-Budgeted Statistically Equivalent Signature (SES) algorithm	maxK = 2, alpha = 0.01, budget = 3 * nvars	Classification Decision Tree with Deviance splitting criterion	minimum leaf size = 3, alpha = 0.05	0.7725496831954634	00:00:40.40983	false
562	Constant Removal, Standardization	LASSO Feature Selection	penalty = 1.0	Support Vector Machines	kernel = 'Polynomial Kernel', cost =	0.9159140802363309	00:03:40.220410	false

Configuration	Preprocessing	Name	Hyperparams	Name	Hyperparams	Performance (unadjusted)	Time (miliseconds)	Dropped
				(SVM) of type C-SVC	1.0, gamma = 0.01, degree = 2			
563	Constant Removal, Standardization	Test-Budgeted Statistically Equivalent Signature (SES) algorithm	maxK = 3, alpha = 0.05, budget = 3 * nvars	Classification Random Forests with Deviance splitting criterion	ntrees = 100, minimum leaf size = 4	0.8857520711740662	00:01:04.64138	false
564	Constant Removal, Standardization	Test-Budgeted Statistically Equivalent Signature (SES) algorithm	maxK = 3, alpha = 0.05, budget = 3 * nvars	Classification Random Forests with Deviance splitting criterion	ntrees = 500, minimum leaf size = 2	0.8897023692420112	00:01:04.64198	false
565	Constant Removal, Standardization	LASSO Feature Selection	penalty = 0.5	Support Vector Machines (SVM) of type C-SVC	kernel = 'Polynomial Kernel', cost = 10.0, gamma = 10.0, degree = 2	0.5255422681893269	00:03:40.220179	false
566	Constant Removal, Standardization	LASSO Feature Selection	penalty = 0.5	Support Vector Machines (SVM) of type C-SVC	kernel = 'Radial Basis Function Kernel', cost = 10.0, gamma = 1.0	0.9163231624868453	00:03:40.220182	false
567	Constant Removal, Standardization	Test-Budgeted Statistically Equivalent Signature (SES) algorithm	maxK = 3, alpha = 0.05, budget = 3 * nvars	Support Vector Machines (SVM) of type C-SVC	kernel = 'Radial Basis Function Kernel', cost = 1.0, gamma = 0.01	0.7477242848086837	00:01:04.64126	false
568	Constant Removal, Standardization	LASSO Feature Selection	penalty = 0.5	Support Vector Machines (SVM) of type C-SVC	kernel = 'Polynomial Kernel', cost = 10.0, gamma = 0.1, degree = 2	0.7087129444866018	00:03:40.220179	false
569	Constant Removal, Standardization	Test-Budgeted Statistically Equivalent Signature (SES) algorithm	maxK = 3, alpha = 0.01, budget = 3 * nvars	Ridge Logistic Regression	lambda = 0.1	0.8808938291035479	00:00:41.41205	false
570	Constant Removal, Standardization	Test-Budgeted Statistically Equivalent Signature (SES) algorithm	maxK = 3, alpha = 0.05, budget = 3 * nvars	Support Vector Machines (SVM) of type C-SVC	kernel = 'Polynomial Kernel', cost = 10.0, gamma = 1.0, degree = 2	0.7585610609907285	00:01:04.64124	false
571	Constant Removal, Standardization	Test-Budgeted Statistically Equivalent Signature (SES) algorithm	maxK = 2, alpha = 0.05, budget = 3 * nvars	Support Vector Machines (SVM) of type C-SVC	kernel = 'Polynomial Kernel', cost = 0.1, gamma = 1.0, degree = 2	0.7913824950269963	00:00:33.33190	false
572	Constant Removal, Standardization	LASSO Feature Selection	penalty = 1.0	Classification Random Forests with Deviance splitting criterion	ntrees = 500, minimum leaf size = 3	0.9310438779748242	00:03:40.220477	false
573	Constant Removal, Standardization	LASSO Feature Selection	penalty = 0.5	Support Vector Machines (SVM) of type C-SVC	kernel = 'Polynomial Kernel', cost = 0.01, gamma = 0.1, degree = 2	0.9259947568771097	00:03:40.220179	false

Configuration	Preprocessing	Name	Hyperparams	Name	Hyperparams	Performance (unadjusted)	Time (miliseconds)	Dropped
574	Constant Removal, Standardization	LASSO Feature Selection	penalty = 0.5	Support Vector Machines (SVM) of type C-SVC	kernel = 'Radial Basis Function Kernel', cost = 0.01, gamma = 1.0	0.9204854182475666	00:03:40.220181	false
575	Constant Removal, Standardization	Test-Budgeted Statistically Equivalent Signature (SES) algorithm	maxK = 2, alpha = 0.05, budget = 3 * nvars	Support Vector Machines (SVM) of type C-SVC	kernel = 'Polynomial Kernel', cost = 0.01, gamma = 1.0, degree = 2	0.8874879383191403	00:00:33.33190	false
576	Constant Removal, Standardization	Test-Budgeted Statistically Equivalent Signature (SES) algorithm	maxK = 2, alpha = 0.05, budget = 3 * nvars	Support Vector Machines (SVM) of type C-SVC	kernel = 'Radial Basis Function Kernel', cost = 0.1, gamma = 10.0	0.8019858445050262	00:00:33.33193	false
577	Constant Removal, Standardization	Test-Budgeted Statistically Equivalent Signature (SES) algorithm	maxK = 2, alpha = 0.01, budget = 3 * nvars	Classification Random Forests with Deviance splitting criterion	ntrees = 500, minimum leaf size = 3	0.8864234657073533	00:00:41.41121	false
578	Constant Removal, Standardization	Test-Budgeted Statistically Equivalent Signature (SES) algorithm	maxK = 3, alpha = 0.01, budget = 3 * nvars	Classification Random Forests with Deviance splitting criterion	ntrees = 500, minimum leaf size = 2	0.8866440110046248	00:00:41.41269	false
579	Constant Removal, Standardization	Test-Budgeted Statistically Equivalent Signature (SES) algorithm	maxK = 3, alpha = 0.05, budget = 3 * nvars	Support Vector Machines (SVM) of type C-SVC	kernel = 'Polynomial Kernel', cost = 10.0, gamma = 1.0, degree = 3	0.7662262786943094	00:01:04.64125	false
580	Constant Removal, Standardization	Test-Budgeted Statistically Equivalent Signature (SES) algorithm	maxK = 3, alpha = 0.05, budget = 3 * nvars	Support Vector Machines (SVM) of type C-SVC	kernel = 'Polynomial Kernel', cost = 1.0, gamma = 0.1, degree = 2	0.8263039887080807	00:01:04.64124	false
581	Constant Removal, Standardization	LASSO Feature Selection	penalty = 0.5	Classification Random Forests with Deviance splitting criterion	ntrees = 100, minimum leaf size = 2	0.9291807581449525	00:03:40.220187	false
582	Constant Removal, Standardization	Test-Budgeted Statistically Equivalent Signature (SES) algorithm	maxK = 2, alpha = 0.05, budget = 3 * nvars	Support Vector Machines (SVM) of type C-SVC	kernel = 'Polynomial Kernel', cost = 0.01, gamma = 1.0, degree = 3	0.874481620964997	00:00:33.33190	false
583	Constant Removal, Standardization	LASSO Feature Selection	penalty = 0.5	Classification Random Forests with Deviance splitting criterion	ntrees = 100, minimum leaf size = 3	0.9316902903859425	00:03:40.220187	false
584	Constant Removal, Standardization	LASSO Feature Selection	penalty = 1.0	Classification Random Forests with Deviance splitting criterion	ntrees = 100, minimum leaf size = 3	0.93945504623254	00:03:40.220422	false

Configuration	Preprocessing	Name	Hyperparams	Name	Hyperparams	Performance (unadjusted)	Time (miliseconds)	Dropped
585	Constant Removal, Standardization	Test-Budgeted Statistically Equivalent Signature (SES) algorithm	maxK = 3, alpha = 0.05, budget = 3 * nvars	Classification Random Forests with Deviance splitting criterion	ntrees = 100, minimum leaf size = 3	0.889528665423806	00:01:04.64138	false
586	Constant Removal, Standardization	Test-Budgeted Statistically Equivalent Signature (SES) algorithm	maxK = 3, alpha = 0.01, budget = 3 * nvars	Classification Random Forests with Deviance splitting criterion	ntrees = 500, minimum leaf size = 3	0.8890407333501961	00:00:41.41296	false
587	Constant Removal, Standardization	Test-Budgeted Statistically Equivalent Signature (SES) algorithm	maxK = 2, alpha = 0.05, budget = 3 * nvars	Support Vector Machines (SVM) of type C-SVC	kernel = 'Radial Basis Function Kernel', cost = 0.1, gamma = 1.0	0.8744812306193381	00:00:33.33192	false
588	Constant Removal, Standardization	LASSO Feature Selection	penalty = 1.0	Classification Random Forests with Deviance splitting criterion	ntrees = 100, minimum leaf size = 2	0.9337548285758005	00:03:40.220424	false
589	Constant Removal, Standardization	LASSO Feature Selection	penalty = 1.0	Support Vector Machines (SVM) of type C-SVC	kernel = 'Polynomial Kernel', cost = 1.0, gamma = 10.0, degree = 3	0.7986382401344039	00:03:40.220410	false
590	Constant Removal, Standardization	LASSO Feature Selection	penalty = 0.5	Support Vector Machines (SVM) of type C-SVC	kernel = 'Polynomial Kernel', cost = 10.0, gamma = 0.1, degree = 3	0.5	00:03:40.220179	false
591	Constant Removal, Standardization	Test-Budgeted Statistically Equivalent Signature (SES) algorithm	maxK = 2, alpha = 0.05, budget = 3 * nvars	Support Vector Machines (SVM) of type C-SVC	kernel = 'Radial Basis Function Kernel', cost = 1.0, gamma = 0.1	0.8611532684422709	00:00:33.33192	false
592	Constant Removal, Standardization	Test-Budgeted Statistically Equivalent Signature (SES) algorithm	maxK = 3, alpha = 0.01, budget = 3 * nvars	Classification Random Forests with Deviance splitting criterion	ntrees = 100, minimum leaf size = 3	0.889528665423806	00:00:41.41218	false
593	Constant Removal, Standardization	LASSO Feature Selection	penalty = 1.0	Support Vector Machines (SVM) of type C-SVC	kernel = 'Polynomial Kernel', cost = 0.01, gamma = 10.0, degree = 3	0.855061534089667	00:03:40.220410	false
594	Constant Removal, Standardization	LASSO Feature Selection	penalty = 1.5	Classification Random Forests with Deviance splitting criterion	ntrees = 500, minimum leaf size = 3	0.9321095216235882	00:03:39.219785	false
595	Constant Removal, Standardization	Test-Budgeted Statistically Equivalent Signature (SES) algorithm	maxK = 2, alpha = 0.05, budget = 3 * nvars	Support Vector Machines (SVM) of type C-SVC	kernel = 'Linear Kernel', cost = 1.0	0.8896153221600791	00:00:33.33191	false
596	Constant Removal,	Test-Budgeted	maxK = 2, alpha = 0.01,	Classification Random	ntrees = 100, minimum leaf	0.8794159804389983	00:00:40.40992	false

Configuration	Preprocessing	Name	Hyperparams	Name	Hyperparams	Performance (unadjusted)	Time (miliseconds)	Dropped
	Standardization	Statistically Equivalent Signature (SES) algorithm	budget = 3 * nvars	Forests with Deviance splitting criterion	size = 3			
